# Protonation‐Induced Electron Density Redistribution Facilitates Aggregation in PNNP Dicopper Hydride Complexes

**DOI:** 10.1002/chem.202502507

**Published:** 2025-12-16

**Authors:** Roel Laurentius Maria Bienenmann, Chattawat Thangsrikeattigun, Alexandra Julia Schanz, Martin Lutz, Mu‐Hyun Baik, Daniël Laurens Johannes Broere

**Affiliations:** ^1^ Organic Chemistry and Catalysis Institute For Sustainable and Circular Chemistry Utrecht University Utrecht Netherlands; ^2^ Department of Chemistry Korea Advanced Institute of Science and Technology (KAIST) Daejeon Republic of Korea; ^3^ Center For Catalytic Hydrocarbon Functionalizations Institute For Basic Science (IBS) Daejeon Republic of Korea; ^4^ Structural Biochemistry Bijvoet Centre for Biomolecular Research Faculty of Science Utrecht University Utrecht Netherlands

**Keywords:** Copper, Aggregation, Bimetallic, Interaction energies, Hydride

## Abstract

Copper(I) hydride complexes often exhibit diverse aggregation behavior that can profoundly influence their structure and reactivity. In this study, we investigate a series of dicopper hydride complexes supported by PNNP expanded pincer ligands that vary in both aggregation and ligand protonation state. Using a combined experimental and computational approach, we examine how these properties correlate and identify key structural and electronic factors that govern aggregation. Our analysis shows that electrostatic stabilization is reduced in charged monomers relative to the neutral system, making dimerization less favorable. At the same time, protonation of the ligands redistributes electron density at the hydride‐bridged dicopper core, lowering interfragment overlap of occupied orbitals, which facilitates dimer formation in more protonated species. These results highlight the importance of ligand‐controlled electronic structure in dictating the aggregation behavior of copper hydride complexes.

## Graphical abstract

Copper(I) hydride complexes often exhibit diverse aggregation behavior that can profoundly influence their structure and reactivity. In this study we use a combined experimental and computational approach to identify key structural an electronic factors that govern aggregation in PNNP dicopperhydride complexes.

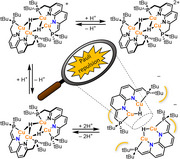

## 1 INTRODUCTION

Copper(I) complexes are widely used in both stoichiometric reactions and homogeneous catalysis, and they have become key tools in modern synthetic chemistry [1, 2, 3, 4, 5, 6]. These complexes mediate a wide range of transformations, including reductions, alkylations, and borylations, demonstrating their remarkable versatility. A distinctive feature of copper(I) chemistry is its tendency to form multi‐copper aggregates [7, 8, 9, 10]. Among these, copper(I) hydride clusters are particularly notable for their ability to reduce unsaturated substrates, functioning effectively in both stoichiometric and catalytic contexts [3, 11, 12]. Importantly, the reactivity of these hydride clusters depends strongly on their nuclearity [13]: some reactions proceed only when mononuclear species dissociate from larger aggregates [14, 15].

**SCHEME 1:**
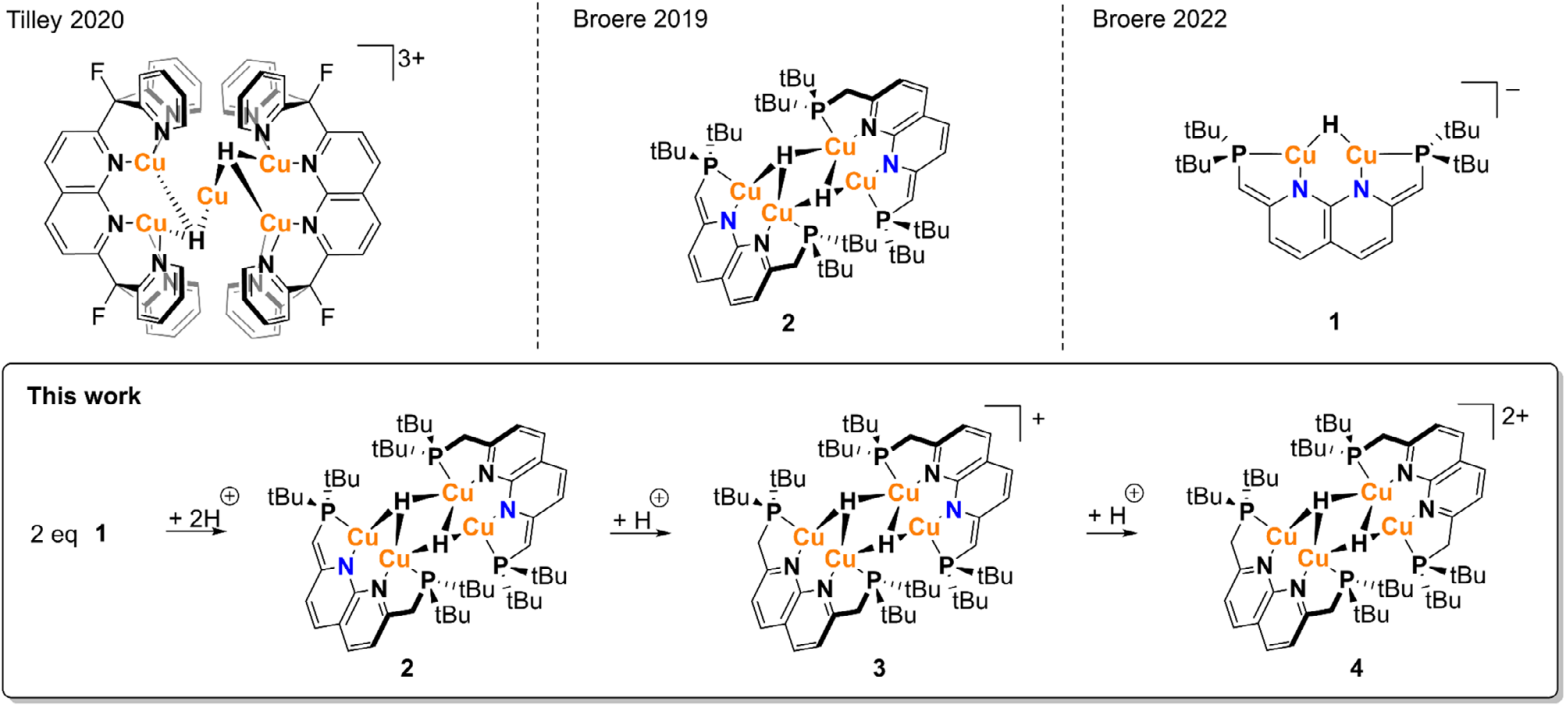
Previously reported DPFN pentacopper(I) hydride complex from Tilley et al. [32]. and the previously reported PNNP copper(I) hydride complexes from our group [29, 33].

Recent years have seen growing interest in understanding how nuclearity affects the reactivity of organometallic complexes, motivated by the potential benefits of metal‐metal cooperativity in catalysis, which can enhance both catalytic efficiency and selectivity [16, 17, 18, 19, 20, 21, 22]. To harness such effects, ligands have been designed that can bind multiple metal centers in close proximity, stabilizing multinuclear frameworks and enabling cooperative interactions. Such ligand designs have greatly advanced our understanding of how nuclearity modulates reactivity [23, 24, 25, 26, 27, 28, 29, 30]. Notably, Tilley and co‐workers pioneered the use of the dinucleating DPFN ligand to access unique copper hydride clusters, including a pentacopper complex (Scheme 1) [31, 32]. Formation of this complex involves partial disproportionation of a dicopper precursor, underscoring the strong thermodynamic preference for higher nuclearity in such systems.

**SCHEME 2:**
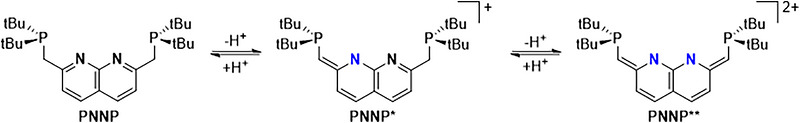
The single and double deprotonation of the PNNP ligand concomitant with the partial or full dearomatization of the ligand, respectively [33].

Another example of a dinucleating ligand is the expanded PNNP pincer that we developed recently [33]. This ligand can be reversibly deprotonated once or twice at its methylene linkers, yielding partially (PNNP*) or fully (PNNP**) dearomatized species (Scheme 2) [33, 34]. These deprotonated states not only enable metal‐ligand cooperative bond activation, but also alter the rigidity and electronic structure of the ligand by converting the nitrogen donors from neutral to anionic [33, 34].

Previously, we showed that the neutral [PNNP*Cu_2_H]_2_ complex (**2**, Scheme 1) exists as a dimer in both solid state and solution [33], while its dianionic counterpart ([PNNP**Cu_2_H]K18‐crown‐6, **1**) is monomeric [29]. We initially attributed this monomerization to increased Coulombic repulsion (i.e., electrostatic repulsion between like‐charged entities) in the anionic state [29]. However, the full dearomatization of the ligand also modifies other factors, such as the geometry, rigidity, and π‐conjugation. Hernández Sánchez and Shee recently showed that in the absence of bridging ligands, various noncovalent interactions together can also provide a driving force for aggregation in Cu(I) complexes [35]. Since nuclearity strongly influences the reactivity of copper(I) hydride complexes, it is crucial to elucidate which factors determine dimerization in this family of compounds. Such conceptual insights will aid in designing copper(I) complexes with tailored aggregation behavior.

Herein, we present a systematic investigation of the aggregation behavior of PNNP‐supported copper hydrides in various protonation states (Scheme 2). By synthesizing and characterizing cationic and dicationic analogues of complex **2**, we expand the series to include neutral, monoanionic, monocationic, and dicationic species (**1**–**4,** Scheme 1, bottom). Combining solid‐state and solution studies with computational analyses, including energy decomposition analysis, we identify the key structural and electronic factors that dictate the aggregation behavior of these copper hydride complexes.

## 2 RESULTS AND DISCUSSION

### 2.1 Synthesis of Monocationic Complex 3 and Dicationic Complex 4

**SCHEME 3:**
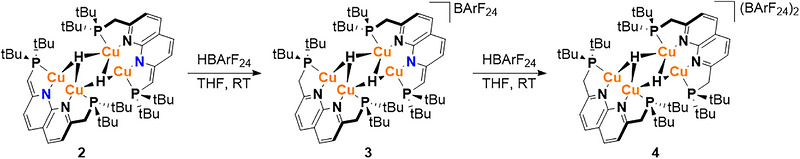
The synthesis of complex **3** and complex **4**.

To synthesize the cationic analogues of complexes **1** and **2**, complex **2** was protonated with Brookhart's acid (HBArF_24_, where BArF_24_ = tetrakis(3,5‐bis(trifluoromethyl)phenyl)borate). Upon adding 1 eq of HBArF_24_ to a THF solution of **2** (Scheme 3), the color of the mixture changed from red to brown/orange. From this mixture, complex **3** was isolated in 92% yield. The ^1^H NMR spectrum of **3** in THF‐d_8_ features six doublets (at 8.56, 7.86, 7,04, 6.54, 6.44, and 6.32 ppm) in the aromatic region, which correspond to the naphthyridine backbones with the two most downfield signals integrating to 2H each and the other four to 1H each. The first two signals indicate a neutral **PNNP** ligand, whereas the other four are consistent with a partially dearomatized PNNP* ligand. These signals are indicative of a mixed protonation state Cu_4_H_2_ dimer in which one of the ligands is neutral, and one is monoanionic. The signals for the methine linker (3.93 ppm) that integrate to 1H and those of the methylene linkers (3.88, 3.71, and 3.32 ppm) that integrate to 2H each align with such a mixed protonation state complex. Geminal coupling (^1^ *J_H‐H_* = 17.6 Hz) is observed between the methylene resonances at 3.88 and 3.71 ppm, which is indicative of the dimeric nature of this complex in solution [33]. It is interesting to note that of the signals for the tert‐butyl (tBu) groups in **3**, the two signals that correspond to these groups on the aromatic sides of the ligand, are much broader than those observed in other PNNP complexes, including **2** and **4** (see below). The reason for this broadening is unclear, but we hypothesize that it is caused by a fluxional process on a similar timescale as the ^1^H NMR measurement.

The ^31^P NMR spectrum of complex **3** features one broad resonance at 40.1 ppm and a sharp resonance at 13.7 ppm, which is also in line with the assignment of **3** as a mixed protonation state Cu_4_H_2_ dimer. To observe the hydride, we measured the ^2^H NMR spectrum of the deuterated analogue of **3**, which was synthesized using the previously reported deuterated analogue of **2** as starting material [33]. Here, one resonance at 1.27 ppm was found for both hydrides in **3**, which overlaps with the tBu signals of **3** in the ^1^H NMR spectrum. We reason that, although the two hydrides are in slightly different chemical environments, their resonances overlap, and hence, only one peak is observed.

**FIGURE 1:**
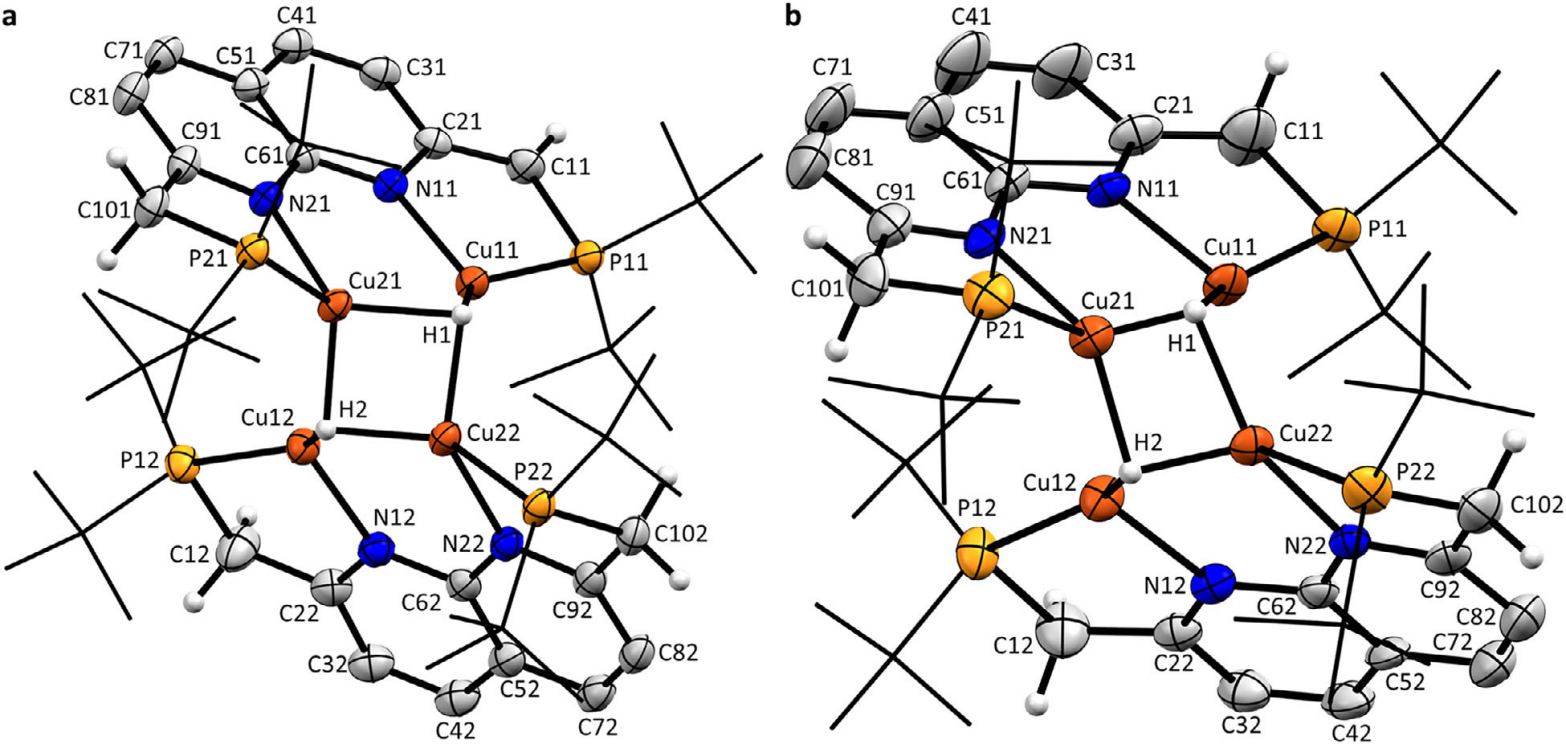
The solid‐state structures of complex **3** (a) and **4** (b). Ellipsoids are drawn at 50% probability, all hydrogen atoms except those on the methylene/methine linkers and the hydrides are omitted, and the tBu groups are drawn in wireframe for clarity. In (a), one disordered BArF_24_ anion, and in (b), two disordered BArF_24_ anions were omitted for clarity.

Crystals of **3** were grown from a saturated benzene solution of the complex with pentane as an anti‐solvent, and these were used for single‐crystal X‐ray structure determination. The solid‐state structure of **3** (Figure 1a) shows a butterfly‐shaped Cu_4_H_2_ core, analogous to the core of **2** [33]. The C12–C22 and C92–C102 distances, of 1.488(3) and 1.505(3) Å, respectively, are consistent with C–C single bonds which show that the **PNNP** ligand on this side of **3** is neutral (Table 1). The C11–C21 and C92–C102 distances of 1.383(3) and 1.512(3) Å, respectively, indicate the presence of a C═C double bond and a C–C single bond, consistent with a partially dearomatized PNNP* ligand on the other side of **3**. The Cu11–Cu21 distance of 2.5115(3) Å on the PNNP* side of **3** is similar to the Cu–Cu distances observed in **2** (2.5106(6) and 2.5122(6) Å) [33]. This is, however, significantly shorter than the Cu12–Cu22 distance of 2.5491(3) Å on the **PNNP** side of **3**.

**TABLE 1:** Selected distances (Å) and torsion angles (°) of complexes **1** [29], **2** [33], **3** and **4**.

Atoms	1 [a,[29]]	2 [33]	3	4
Cu11–Cu21	2.4229(7)	2.5106(6)	2.5115(3)	2.5332(11)
Cu12–Cu22	—	2.4778(5)	2.5491(3)	2.5227(11)
Cu11–P11	2.2386(7)	2.2588(9)	2.2548(5)	2.2508(19)
Cu21–P21	2.2397(7)	2.2846(9)	2.2655(5)	2.284(2)
Cu12–P12	—	2.260(1)	2.2524(6)	2.2493(19)
Cu22–P22	—	2.271(1)	2.2794(5)	2.2670(18)
Cu11–N11	2.047(2)	2.051(3)	2.0387(16)	2.103(5)
Cu21–N21	2.042(2)	2.118(3)	2.0747(16)	2.129(5)
Cu12–N12	—	2.046(2)	2.1109(17)	2.127(5)
Cu22–N22	—	2.124(2)	2.1438(17)	2.146(5)
C11–C21	1.387(3)	1.364(5)	1.383(3)	1.490(10)
C91–C101	1.386(3)	1.509(5)	1.512(3)	1.502(9)
C12–C22	—	1.365(5)	1.488(3)	1.499(9)
C92–C102	—	1.510(5)	1.504(3)	1.509(9)
P11–C11–C21–N11	2.7(3)	−2.1(5)	4.8(3)	−7.4(10)
P21–C101–C91–N21	3.3(3)	17.6(4)	27.3(2)	−29.4(8)
P12–C12–C22–N12	—	−1.4(5)	−10.9(3)	−1.7(9)
P22–C102–C92–N22	—	23.5	38.7(2)	−26.4(8)

^a^ The numbering of **1** [29] is different from that of the other hydride complexes since it is a monomer. The atom numbers in **1** are obtained by omitting the last number (e.g. Cu21 in **2**, **3**, and **4** corresponds to Cu2 in **1**).

To assess the extent to which Coulombic repulsion contributes to the monomerization of copper hydride complexes, we sought to generate a complex featuring two cationic [**PNNP**Cu_2_H]^+^ units. The monomeric form of such a complex would have the same charge as complex **1** but with an opposite sign (i.e., +1 instead of –1), thereby enabling a direct comparison in the influence of charge polarity on aggregation behavior. To this end, a THF solution of complex **2** was treated with 2 eq of HBArF_24_ resulting in a distinct color change of the mixture from red to dark brown/black. From this solution, complex **4** was isolated as a purple powder with a 97% yield (Scheme 3). The ^1^H NMR spectrum of **4** in THF‐d_8_ features two doublets at 8.67 and 7.95 ppm corresponding to the aromatic naphthyridine backbone and one doublet at 3.75 ppm corresponding to the methylene linker. The ^31^P NMR spectrum of **4** features one broad signal at 35.3 ppm. These NMR spectra indicate that **4** contains a neutral **PNNP** ligand and is C_*2v*_ symmetrical in solution. It is important to note that the observed C_*2v*_ symmetry in the NMR spectra makes it ambiguous whether **4** is a monomer or dimer in solution (see below). Analogous to complex **3**, the chemical shift of the hydride in **4** (1.47 ppm) was determined by ^2^H NMR on the deuteride analogue.

Crystals of **4**, which were used for single crystal X‐ray structure determination, were grown from a THF solution with pentane as an anti‐solvent. The crystal structure of **4** (Figure 1b and Table 1) revealed a dimeric complex analogous to the structures observed for complexes **2** and **3** [33]. However, complex **1** exhibits a contrasting behavior, being monomeric in the solid state [29]. The C91–C101, C12–C22, C92–C102, and C11–C21 bond lengths in **4** are between 1.490(10) and 1.509(9) Å [36], which indicates that both ligands in **4** are neutral **PNNP** moieties, in line with the observations from the NMR analysis. The Cu–Cu distances within each **PNNP** ligand in **4** are slightly longer (2.5227(11) and 2.5232(11) Å) than those found in **2** and on the partially dearomatized side of **3** (between 2.5106(6) and 2.5122(6) Å) [33]. However, they are marginally shorter than the Cu12–Cu22 distance (2.5491(3)) in **3**. The Cu–Cu distances in this family of PNNP copper hydride complexes (**1**–**4**) follow the general trend of dicopper PNNP complexes, in which the Cu–Cu distance decreases upon deprotonation of the ligand [34]. It is interesting to note that one of the two methylene linkers on each ligand in **4** has a significantly larger torsion angle than the other, as can be seen comparing the P12–C21–C22–N12 (–1.7(9)°) and P11–C11–C21–N11 (–7.4(10)°) torsion angles with the P22–C102–C92–N12 (–26.4(8)°) and P21–C101–C91–N21 (–29.4(8)°) torsion angles. This suggests that, at least in the solid state, the increased flexibility in **4** compared to **2** or **3** does not lead to a different or more stable dimer geometry since similar torsion angles are observed in these structures.

### 2.2 Aggregation state of the Copper Hydrides in Solution

The symmetry of the molecular structure of **4** in the crystal is approximately C_*2*_, which contrasts with the observed C_*2v*_ symmetry of **4** in solution. To probe whether the observed C_*2v*_ symmetry in solution indicates that **4** is a monomer in solution, or whether it is caused by a fluxional process, we performed diffusion‐oriented spectroscopy (DOSY) NMR on a mixture of **3** and **4**. The DOSY NMR spectrum shows that **4** has a smaller diffusion coefficient than **3** due to the co‐diffusion of the counterions. Since the symmetry in the ^1^H NMR spectrum of **3** shows that it is a dimer in solution, this indicates that **4** is also dimeric in solution (see Figure S17 for details and additional discussion). Interestingly, the ^2^H–^31^P coupling constant also indicates the aggregation state of these copper hydrides in solution due to its sensitivity to the local geometry around the bridging hydride/deuteride ligand. Complexes **2**, **3,** and **4** show no significant ^2^H–^31^P coupling for the deuteride resonance in the ^2^H NMR spectra of their deuterated analogues [33]. In contrast, the ^2^H NMR spectrum of the deuterated analogue of complex **1** features a clear triplet (^2^ *J*_D‐p_ = 3.5 Hz) for its deuteride ligand [29]. These observations are, therefore, also in line with **4** being a dimer in solution (see Supporting information Section 2.2).

### 2.3 Absence of a Mixed Protonation state Anionic Complex

**FIGURE 2:**
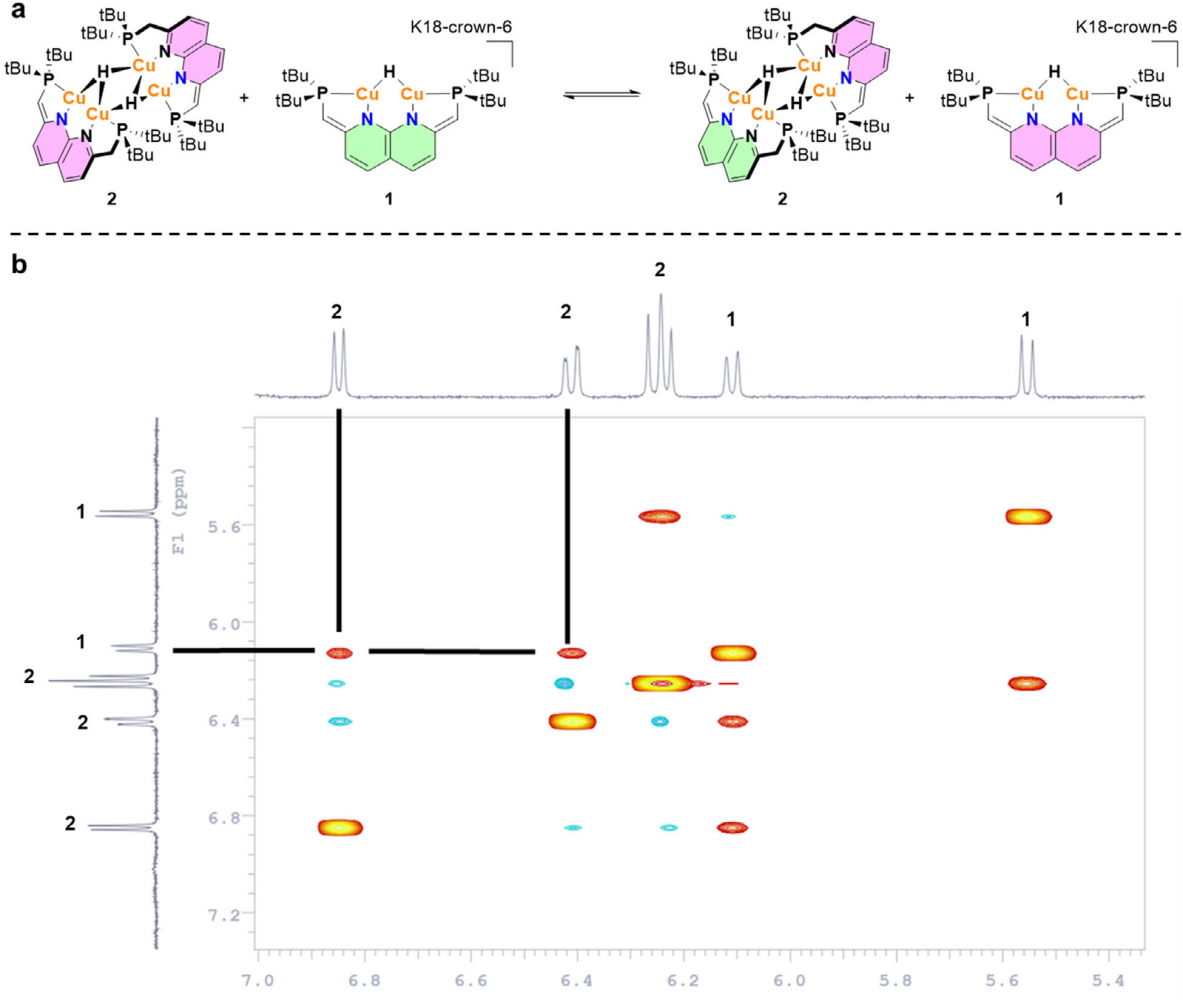
(a) The proposed protonation/deprotonation equilibrium between **1** and **2**. (b) The EXSY NMR spectrum of the mixture of **1** and **2** in THF‐d_8_ at 60°C.

The existence of mixed protonation state complex **3** suggests that potentially an anionic mixed protonation state complex might also be attainable, although trace impurities of **2** in the reported spectra of **1** already imply that such a species may not exist [29]. To verify this, complexes **1** and **2** were mixed, and ^1^H NMR analysis shows that both exist together in solution without forming a third mixed protonation species. To verify that this mixture is not due to the slow kinetics of protonation/deprotonation or the aggregation state change, we used exchange spectroscopy (EXSY) NMR. At room temperature, no exchange between the resonances of **1** and **2** was observed; however, at 60°C, there are EXSY cross‐peaks between the signals of the two species (Figure 2). The increase in temperature is likely required to increase the rate of this exchange sufficiently to observe it on the EXSY time scale. The presence of the EXSY cross‐peaks confirms our hypothesis that **1** and **2** are in equilibrium with each other in solution, proving that an anionic mixed protonation state complex does not exist in the THF solution.

### 2.4 Theoretical Studies

**FIGURE 3:**
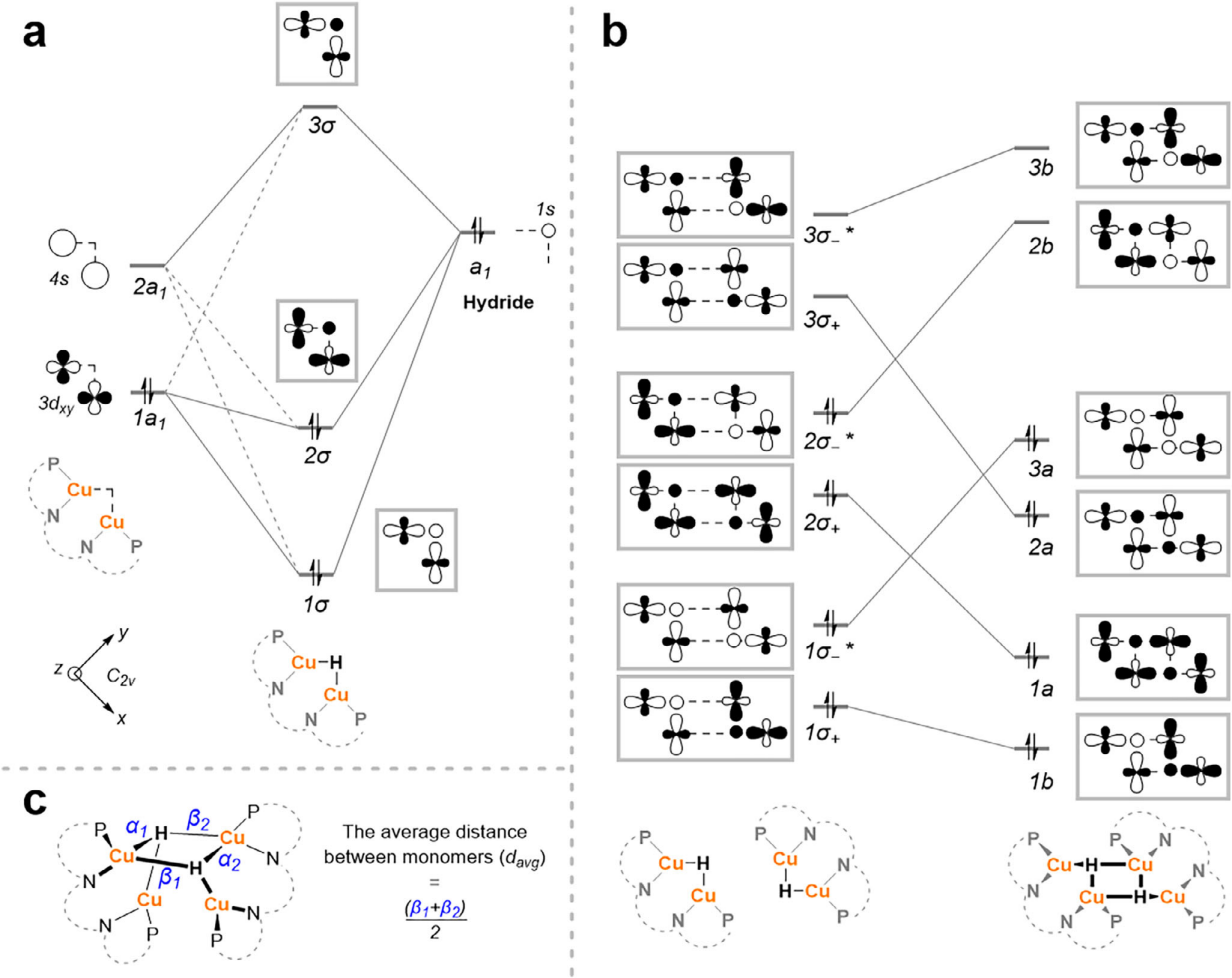
(a) Simplified orbital interaction diagram of the Cu_2_H core. (b) Conceptual orbital correlation diagram illustrating the electronic evolution during dimerization of a Cu_2_H monomer. (c) Conceptual 3D representation of the dimer of Cu_2_H(PNNP), including the definition of the average intermonomeric distance.

The experimental findings on our series of copper hydride complexes (**1**–**4**) show that only **1** exists as a monomer, while **2**, **3** [37], and **4** form dimers. For simplicity of further analysis, counterions were omitted and the structures are denoted as “**‐mono**” or “**‐dimer**” according to their aggregation state. In the Cu_2_H cores of **1‐mono**, **2‐mono**, and **4‐mono**, the copper centers are in the +1 oxidation state, corresponding to a *d^10^* electronic configuration. The bonding within each Cu_2_H unit can be described in terms of orbital interactions between the Cu_2_ fragment and the bridging hydride ligand (Figure 3a). The occupied *1a_1_* orbital from the in‐phase combination of Cu *3d_xy_* orbitals interacts with the occupied *a_1_* orbital of the hydride and gives rise to *1σ* and *2σ* molecular orbitals (MOs). While this interaction is formally antibonding, the presence of an unoccupied *2a_1_* orbital, composed of Cu *4s* orbitals and possessing the same symmetry, enables mixing that stabilizes the resulting *1σ* and *2σ* molecular orbitals. This interaction results in slight net bonding character to the Cu–H interactions in the monomeric Cu_2_H framework.

Upon dimerization through an edge‐to‐edge Cu–H interaction, two Cu_2_H monomers combine to form a Cu_4_H_2_ dimer (Figure 3b). The associated bonding changes can be understood through an orbital correlation diagram constructed from the *σ*‐type orbitals of the monomer units. Linear combinations of these orbitals yield in‐phase (*σ_+_*) and out‐of‐phase (*σ_−_*) molecular orbitals in the dimer. As the intermonomer Cu–Cu distance shortens (Figures 3b and 3c), the *1σ_+_*, *2σ_+_*, and *3σ_+_* orbitals are significantly stabilized through constructive overlap and give rise to *1b*, *1a*, and *2a* molecular orbitals of the dimer, respectively. In contrast, 1σ_−_*, 2σ_−_*, and 3σ_−_* orbitals are destabilized due to enhanced antibonding interactions and correspond to *3a*, *2b*, and *3b* molecular orbitals, respectively. Among these, the occupied *3a* molecular orbital exhibits slight antibonding character between monomeric units due to *4s3d_xy_* mixing of each Cu center, as previously described in the bonding structure of the monomer. These delocalized interactions collectively contribute to the overall stabilization of the Cu_4_H_2_ dimer, facilitating dimerization. Experimentally, dimers such as **2**, **3** and **4** adopt a butterfly‐like structure in THF (Figure 3c), in contrast to the ladder‐type structures commonly observed in lithium amide dimers [38]. This structural preference reflects the distinct electronic structure of the Cu_2_H motif.

**FIGURE 4:**
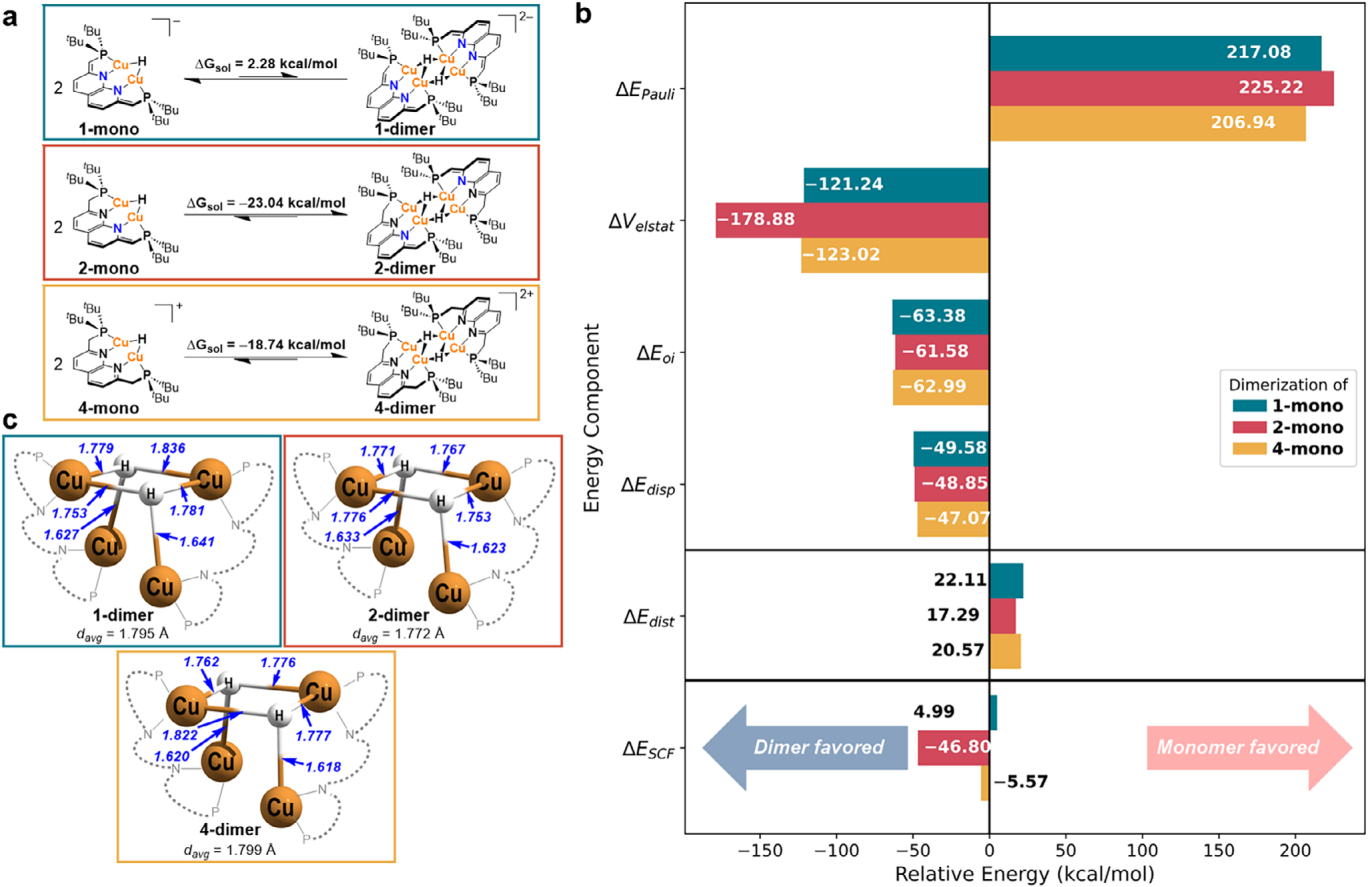
(a) Dimerization free energies of complexes **1**, **2**, and **4** calculated at the B3LYP‐D3/def2‐TZVP//B3LYP‐D3/def2‐SVP, def2‐TZVP(Cu) level with CPCM(THF) implicit solvation at 298.15 K. (b) Distortion‐interaction and energy decomposition analyses associated with the dimerization of **1‐mono**, **2‐mono**, and **4‐mono** into their respective dimers. (c) Optimized structures and key intermonomer distances (in Å) for **1‐dimer**, **2‐dimer**, and **4‐dimer** (only the Cu_4_H_2_ core is shown).

To elucidate the origin of protonation‐dependent aggregation [29, 33], we calculated the dimerization free energies (ΔG_sol_) for **1**, **2,** and **4**. The computed dimerization energies align well with the experimental observations: dimerization of **1‐mono** is energetically disfavored, whereas it is favorable for **2‐mono** and **4‐mono** (Figure 4a). At room temperature, the entropic penalty (–T∆S) of dimerization is 18.0, 18.5, and 17.6 kcal/mol for **1‐mono**, **2‐mono**, and **4‐mono**, respectively. In all three systems, this penalty remains consistently around 18 kcal/mol, primarily reflecting the loss of translational entropy as two similarly sized monomers associate to form a dimer.

Because dimerization is inherently entropically disfavored, electronic and solvation effects play a critical role in facilitating aggregation. In a polar solvent such as THF, solvation significantly stabilizes positively charged species [39], favoring cations over anionic and neutral counterparts (see Molecular Electrostatic Potential analysis in Supporting Information section 2.3), and thereby contributes more substantially to the aggregation of **4‐mono** compared to **1‐mono** and **2‐mono**. As a result, solvation significantly favors the dimerization of **4‐mono** (ΔΔG^solv^ = –10.1 and –36.5 kcal/mol relative to **1‐mono** and **2‐mono**, respectively), while a modest electronic driving force (ΔE_SCF_ = –5.6 kcal/mol) also supports the process. For **2‐mono**, although the solvation is considerably weaker than for **4‐mono**, the electronic contribution to dimerization (ΔE_SCF_ = –46.8 kcal/mol) is sufficiently large to result in a favorable dimerization with ΔG_sol_ of –23.0 kcal/mol. In contrast, the anionic complex **1‐mono** remains monomeric (ΔG_sol_ = 2.3 kcal/mol), as both electronic and entropic factors strongly disfavor dimerization.

To better understand the electronic factors governing dimerization, we performed distortion‐interaction [40] and energy decomposition analyses [41]. Within the framework of the energy decomposition, structural distortion (∆E_dist_) and Pauli repulsion (∆E_Pauli_) disfavor dimerization, whereas electrostatic (∆V_elstat_), orbital interaction (∆E_oi_), and dispersion (∆E_disp_) interactions favor it (Figure 4b). The dimerization of **1‐mono** and **2‐mono** exhibits similar energy partitioning patterns, consistent with their comparable Cu_4_H_2_ core structures. However, the dianionic **1‐dimer** and dicationic **4‐dimer** display less favorable ∆V_elstat_ values compared to the neutral **2‐dimer**. Although the electrostatic term remains attractive in all cases, its magnitude decreases when the interacting fragments bear like charges, reflecting the reduced net electrostatic stabilization due to repulsive interactions between regions of similar charge density. This reduced electrostatic attraction contributes to their higher overall dimerization energies. Notably, while **4‐dimer** also exhibits a similar attenuation in ∆V_elstat_ as **1‐dimer**, the effect is not sufficiently destabilizing to disfavor dimerization.

The intermonomer distances of optimized dimer structures reveal key features of distinct dimers, where **2‐dimer** exhibits the shortest distance of 1.772 Å, followed by **1‐dimer** at 1.795 Å, and **4‐dimer** at 1.799 Å (Figure 4c). These geometrical differences align with the electrostatic interactions: the stronger electrostatic attraction in **2‐dimer** enables a closer approach of the fragments, whereas the weaker attraction in **1‐dimer** and **4‐dimer** restricts further contraction at the Cu_2_H interfaces.

Direct comparison of **1‐mono** and **4‐mono** shows that these exhibit nearly identical electrostatic interactions as expected from their equal but opposite charges. However, **4‐dimer** displays 10.1 kcal/mol less Pauli repulsion, reversing the electronic energy trend (∆E_SCF_) and favoring aggregation. This reduction in Pauli repulsion originates from decreased orbital overlap between occupied orbitals of interacting fragments. Protonation shifts electron density from the Cu centers toward the methylene linkers of the PNNP ligands (see Supporting Information section 2.4), reducing orbital crowding at the Cu_2_H interface and thereby diminishing interfragment orbital overlap. This is consistent with the slightly increased intermonomer distance observed in **4‐dimer** (Figure 4c), which further attenuates Pauli repulsion and facilitates aggregation.

These findings corroborate our previous hypothesis that like‐charge repulsion, reflecting the expected Coulombic repulsion between charged fragments, significantly influences the aggregation behavior of this family of PNNP copper(I) hydride complexes (**1**–**4**) [29]. However, the present analysis refines this understanding by demonstrating that, in cases involving oppositely charged species (i.e., **1‐mono** and **4‐mono**), electrostatic interactions alone do not dictate the aggregation outcome. Instead, Pauli repulsion emerges as the decisive factor. In particular, the lower Pauli repulsion observed in **4‐dimer** stems from protonation‐induced electronic reorganization, which localizes electron density within each monomer and reduces orbital overlap across the Cu_4_H_2_ core, enabling aggregation. This attenuation of electronic repulsion facilitates dimerization, despite the inherent electrostatic cost associated with assembling charged units.

## 3 CONCLUSIONS

In conclusion, we investigated a series of dicopper hydride complexes supported by PNNP expanded pincer ligands in different protonation states. In its doubly deprotonated form, the PNNP ligand features a dianionic, fully dearomatized naphthyridine core that stabilizes a discrete anionic dicopper hydride complex. In contrast, complexes containing neutral or monocationic PNNP ligands (or both) dimerize to form neutral, monocationic, or dicationic tetracopper dihydride species. The observed aggregation difference between the cationic and anionic species therefore indicates that the aggregation is driven by factors beyond simple like‐charged Coulombic repulsion.

Computational analysis shows that the stabilization contribution from electrostatic interactions is significantly reduced for both cationic and anionic dicopper hydride fragments compared to the neutral system, thereby disfavoring dimerization relative to the neutral case. However, in cationic systems, protonation redistributes electron density, reducing crowding at the hydride‐bridged dicopper core and thereby facilitating aggregation. The positive charge on each copper center is increased, and the electron density is contracted, allowing cationic monomers to approach without significant penetration of their electron clouds. In the energy decomposition analysis, this manifests as reduced Pauli repulsion. Additionally, bridging hydrides, bearing partial negative charge, provide attractive interactions with the copper centers of the opposite monomer, further mitigating electrostatic repulsion. In the anionic system, deprotonation expands the electron density around the copper hydride fragment, effectively increasing its ionic radius and enhancing overlap of occupied orbitals at close approach. This expanded distribution significantly increases Pauli repulsion, which ultimately prevents aggregation, despite the potential for hydride bridging. While solvation in THF also provides additional stabilization of the cationic complex relative to the anionic one, our analysis shows that this does not fully account for the observed aggregation trends which are also present in the solid state and in gas‐phase calculations. Overall, these results show that aggregation in our PNNP dicopper hydride systems is decisively impacted by protonation‐dependent modulation of electron density.

## 4 Methodology

**General experimental considerations**: All manipulations were performed under N_2_ atmosphere inside a glovebox or on a Schlenk line using dried and degassed chemicals and solvents. Complex **1**, complex **2** and HBArF were prepared according to literature procedure [30, 33, 42]. Further experimental details are described in the Supporting Information.

**Synthesis of Complex 3**: Complex **2** (18.7 mg, 16.3 µmol, 1 eq) was dissolved in THF (3 mL) and to this, freshly dissolved HBArF (16.5 mg, 16.3 µmol, 1 eq) in THF (2 mL) was added dropwise during which the color changed from red to brown/orange. After stirring for 1 h, the solvent was removed under dynamic vacuum yielding an orange/brown film. Addition of pentane (1.5 mL) yielded a suspension which was filtered, and the residue was washed with additional pentane (0.5 mL). The solid was extracted with THF (1 mL) and the extract was dried under dynamic vacuum, yielding a film again. The film was stripped with pentane (2 mL) twice, yielding complex **3** (30.3 mg) in 90% yield as an orange powder. Crystals suitable for single crystal X‐ray determination were grown from a saturated benzene solution of **3** (0.5 mL) and pentane (2.5 mL) as anti‐solvent. ^1^H NMR (400 MHz, THF‐*d*_8_, 298 K): *δ* 8.56 (d, ^3^ *J_H,H_* = 8.2 Hz, 2H), 7.86 (d, ^3^ *J_H,H_* = 8.3 Hz, 2H), 7.78 (s, 9H), 7.56 (s, 4H), 7.04 (d, ^3^ *J_H,H_* = 7.2 Hz, 1H), 6.54 (dd, ^3^ *J_H,H_* = 9.0, ^4^ *J_H,p_* = 1.8 Hz, 1H), 6.43 (d, ^3^ *J_H,H_* = 7.2 Hz, 1H), 6.32 (d, ^3^ *J_H,H_* = 7.0 Hz, 1H), 3.93 (d, ^2^ *J_H,p_* = 2.3 Hz, 1H), 3.80 (dd, ^1^ *J_H,H_* = 17.6 Hz, ^4^ *J_H,p_* = 8.2 Hz, 2H), 3.71 (bd, ^1^ *J_H,H_* = 17.6 Hz, 2H), 3.32 (d, ^2^ *J_H,p_* = 5.0 Hz, 2H), 1.44 – 1.27 (m, 36H), 1.11 (d, ^3^ *J_H,p_* = 12.8 Hz, 18H), 0.66 (bs, 18H) ppm. ^31^P{^1^H} NMR (162 MHz, THF‐*d*_8_, 298 K): *δ* 41.0 (s), 14.7 (s) ppm. ^19^F NMR (376 MHz, THF‐*d*_8_, 298 K): *δ* ‐63.76 (s) ppm. ^13^C{^1^H} APT (101 MHz, THF‐*d*_8,_ 298 K): *δ* 167.9 (d, *J* = 5.1 Hz), 166.0 (d, *J* = 16.7 Hz), 163.0 (q, *J* = 49.7 Hz), 158.2(d, *J* = 3.3 Hz), 152.1, 141.2, 135.8, 134.8, 130.2 (m), 129.8, 129.7 (d, *J* = 2.6 Hz), 126.6 (d, *J* = 9.0 Hz), 125.7 (d, *J* = 272.8 Hz), 125.3 (d, *J* = 3.2 Hz), 123.5, 121.6, 119.5 (d, *J* = 2.24 Hz), 118.4 (p, *J* = 4.4 Hz), 111.6, 81.8 (d, ^1^ *J_C,p_* = 37.3 Hz), 34.2, 34.1, 33.1(d, ^1^ *J_C,p_* = 5.4 Hz), 30.9 (d, ^2^ *J_C,p_* = 7.9 Hz), 30.6 (d, ^2^ *J_C,p_* = 8.2 Hz), 29.5 (d, ^2^ *J_C,p_* = 9.2 Hz), IR‐ATR (cm^−1^): 2946 (m), 1604 (w), 1542 (w), 1501 (w), 1468 (w), 1418 (m), 1353 (m), 1276 (s), 1126 (s), 887 (w), 860 (w), 838 (w), 822 (w), 713 (w), 682 (m). Anal. Calc. for C_84_H_101_BCu_4_F_24_N_4_P_4_ • (C_5_H_12_)_0.8_: C, 51.08: H, 5.39; N, 2.71. Found C, 51.15; H, 5.34; N, 2.78. The inclusion of 0.8 eq of pentane provides a satisfactory elemental analysis, which is in line with the presence of small amounts of pentane observed in the **^1^** H NMR spectrum.

**Synthesis of Complex 4**: Complex **2** (40.0 mg, 34.9 µmol, 1 eq) was dissolved in THF (4 mL) and to this, a freshly prepared solution of HBArF_24_ (71.7 mg, 70.8 µmol, 2 eq) in THF (4 mL) was added dropwise. The reaction mixture was let to stir for one hour, after which the solvent was evaporated yielding a black solid. This solid was washed with pentane (2 mL) yielding complex **4** as a black solid (104.5 mg, 33.7 µmol, 97%). Crystals suitable for single crystal X‐ray determination were grown from a THF solution of **3** (0.5 mL) and pentane (2.5 mL) as anti‐solvent. ^1^H NMR (400 MHz, THF‐*d*_8_, 298 K): *δ* 8.67 (d, ^3^ *J_H, H_* = 8.3 Hz, 4H), 7.95 (d, ^3^ *J_H, H_* = 8.3 Hz, 4H), 7.78 (s, 16H), 7.56 (s, 8H), 3.77 (d, ^2^ *J_H, p_* = 6.5 Hz, 4H), 1.06 (d, ^3^ *J_H, p_* = 13.3 Hz, 72H) ppm. ^31^P{^1^H} NMR (162 MHz, THF‐*d*_8_, 298 K): *δ* 37.43 (bs) ppm. ^19^F NMR (376 MHz, THF‐*d*_8_, 298 K): *δ* ‐63.76 (s) ppm. ^13^C{^1^H} APT (101 MHz, THF‐*d*_8,_ 298 K): *δ* 168.4 (d, *J* = 4.6 Hz), 162.9 (q, *J* = 49.5 Hz), 151.8, 142.1, 135.7, 130.1 (m), 125.7 (d, *J* = 2.3 Hz), 125.6 (d, *J* = 272.5 Hz), 118.3 (quint, *J* = 3.8 Hz), 33.9 (d, ^1^ *J_C, p_* = 12.9 Hz), 33.5 (d, ^1^ *J_C, p_* = 5.5 Hz), 29.8 (d, ^2^ *J_C, p_* = 7.7 Hz) ppm. IR‐ATR (cm^−1^): 2959 (m), 1604 (m), 1509, 1353 (s), 1275 (s), 1123 (S), 887 (w), 862 (w), 839 (m), 713 (m), 682 (m), 670 (m). Anal. Calc. for C_116_H_114_B_2_Cu_4_F_48_N_4_P_4_: C, 48.45; H, 4.00; N, 1.95. Found C, 49.70; H, 4.39; N, 2.05. We were unable to obtain a satisfactory elemental analysis for **4**, likely due to the inclusion of solvent (THF and pentane) in the final product. However, due to the broad peaks from traces of **3** around 0.8‐1.5 ppm in ^1^H NMR, we cannot accurately assess if and how much pentane is present.

**Preparation of 3D *in situ***: The deuterated analogue of complex **2** (**2D**) was prepared according to literature procedure [33] and without isolating the product, HBArF (24.7 mg, 24.4 µmol, 1.05 eq) freshly dissolved in THF (0.25 mL) was added to the solution of **2D** (23.2 µmol, 1 eq) in THF (1 mL). This mixture was analyzed without purification using ^2^H NMR. ^2^H NMR (61 MHz, THF‐*h*_8_): *δ* 1.27 (s, 5.2 Hz full width half maximum) ppm.

**Preparation of 4D *in situ***: To a solution of **3D** (11.1 µmol) in THF (0.6 mL) prepared as described above, HBArF (11.6 mg, 11.5 µmol, 1.03 eq) freshly dissolved in THF (0.1 mL) was added. This mixture was analyzed without purification using ^2^H NMR. ^2^H NMR (61 MHz, THF‐*h*_8_): *δ* 1.47 (s, 4.4 Hz full width half maximum) ppm.

**Computational details**: Geometry optimizations were carried out at the B3LYP level of theory including Grimme's D3 dispersion correction, employing the def2‐SVP basis set for all atoms except copper, for which def2‐TZVP was used [43, 44, 45, 46, 47, 48, 49]. The def2/J auxiliary basis set and the RIJCOSX approximation were applied throughout to accelerate hybrid functional calculations [50, 51]. Single‐point energy refinements were subsequently performed at the B3LYP‐D3/def2‐TZVP level on the optimized geometries [52]. Analytical vibrational frequency calculations within the harmonic approximation were obtained using the def2‐SVP/def2‐TZVP(Cu) mixed basis set. Solvation energies were calculated using the CPCM model [53] and were performed with the def2‐SVP/def2‐TZVP(Cu) mixed basis at the optimized gas phase geometry with tetrahydrofuran (THF) as the solvent.

## Conflicts of Interest

The authors declare no conflicts of interest.

## Author Contributions

R.L.M.B., C.T., D.L.J.B and M.‐H.B. devised the project. A.J.S. and R.L.M.B. performed the synthesis and characterization of complexes **3** and **4**. M.L. performed the X‐ray diffraction measurements and structure elucidation. C.T. performed the computational studies. R.L.M.B. and C.T. wrote the manuscript with input and editing from D.L.J.B. and M.‐H.B, and input from all authors.

## Supporting information



Experimental procedures, characterization data, spectra for all new compounds, crystallographic data, and Cartesian coordinates of all computed structures.
The NMR datafiles for complexes **3** and **4** can be obtained free of charge from https://doi.org/10.24416/UU01‐WCFVPA. CCDC 2337836 (compound **3**) and 2337837 (compound **4**) contain the supplementary crystallographic data for this paper. These data can be obtained free of charge from The Cambridge Crystallographic Data Centre via www.ccdc.cam.ac.uk/data_request/cif.

**Supporting File 1**: chem70574‐sup‐0002‐DataFile.zip
