# Supplementary material for: Protonation‐Induced Electron Density Redistribution Facilitates Aggregation in PNNP Dicopper Hydride Complexes
Source: Chemistry. 2025 Dec 16;32(30):e02507. doi: 10.1002/chem.202502507 (PMC13472354; doi:10.1002/chem.202502507)
Supplement: Supplementary file 1 [file CHEM-32-e02507-s002.pdf]

Supporting Information

Protonation-Induced Electron Density Redistribution Facilitates Aggregation in PNNP Dicopper Hydride Complexes

Roel L. M. Bienenmann,[1] Chattawat Thangsrikeattigun,[2,3] Alexandra J. Schanz,[1] Martin Lutz,[4] Mu-Hyun Baik,*[,3,2] and Daniël L. J. Broere*[,1]

[1]Organic Chemistry and Catalysis, Institute for Sustainable and Circular Chemistry, Utrecht University, Universiteitsweg 99, 3584 CG, Utrecht, The Netherlands

[2]Department of Chemistry, Korea Advanced Institute of Science and Technology (KAIST), Daejeon, 34141, Republic of Korea

[3]Center for Catalytic Hydrocarbon Functionalizations, Institute for Basic Science (IBS), Daejeon, 34141, Republic of Korea

[4]Structural Biochemistry, Bijvoet Centre for Biomolecular Research, Faculty of Science, Utrecht University, Universiteitsweg 99, 3584 CG Utrecht, The Netherlands

Table of Contents

1. Experimental Methods 3
1.1 General considerations 3
1.2 Synthesis of complex 3 3
1.3 Synthesis of complex 4 8
1.4 NMR spectrum of the deuterated analogue of complex 3 (3D) in situ 12
1.5 NMR spectrum of the deuterated analogue of complex 4 (4D) in situ 12
1.6 DOSY NMR 13
1.7 X-ray crystal structure determination of 3 15
1.8 X-ray crystal structure determination of 4 16
2. Computational Details 17
2.1 Computational methods 17
2.2 Calculation of 2J Coupling Constants 18
2.3 Molecular Electrostatic Potential (MEP) Analysis 18
2.4 Hirshfeld Analysis 20
2.5 DFT-energy components of all optimized geometries 21
2.6 Cartesian coordinates for DFT-optimized structures 22
2.7 Vibrational frequencies for DFT-optimized structures 28
3. References 31

1. Experimental Methods

1.1 General considerations

All manipulations were performed under N_2 atmosphere inside a glovebox or on a Schlenk line unless mentioned otherwise. The ambient temperature in our glovebox is on average ~27°C. Glassware was dried at 130°C in an oven overnight or flame dried prior to using it. Pentane and benzene were dried over 4Å molecular sieves and degassed by bubbling N_2 through the liquid for at least 30 min. THF was distilled over Na/benzophenone ketyl (purple) and degassed by bubbling N_2 through the liquid for at least 30 min. Water content in the solvents was tested using Karl-Fisher titration and tested by titration with a Na/benzophenone ketyl solution. Deuterated THF was degassed using three freeze pump thaw cycles and dried over molecular sieves (4 Å). The water content was tested by titration with a Na/benzophenone ketyl solution. Complex **1** ([PNNP**Cu_2H](K18-crown-6)[1], complex **2** ([PNNP*Cu_2H]$_2$)[2] and HBArF$_{24}$[3] were synthesized according to literature procedures. Other chemicals were obtained from commercial sources. Liquids were degassed using 3 freeze pump thaw cycles before use and solids were degassed under vacuum before use. NMR spectra were recorded on a Varian MRF 400 equipped with a OneNMR probe and Optima Tune system or a Varian VNMR-S-400 equipped with a PFG probe or a 400 MHz Jeol EZCL G system with an HFX probe and 90 G/cm gradient amplifier. All resonances in ^{1}H NMR were referenced to residual solvent peaks. IR-data was recorded on a PerkinElmer SpectrumTwo Infrared Spectrophotometer equipped with an ATR-probe. Elemental analysis was performed by MEDAC ltd. in the United Kingdom.

1.2 Synthesis of complex 3

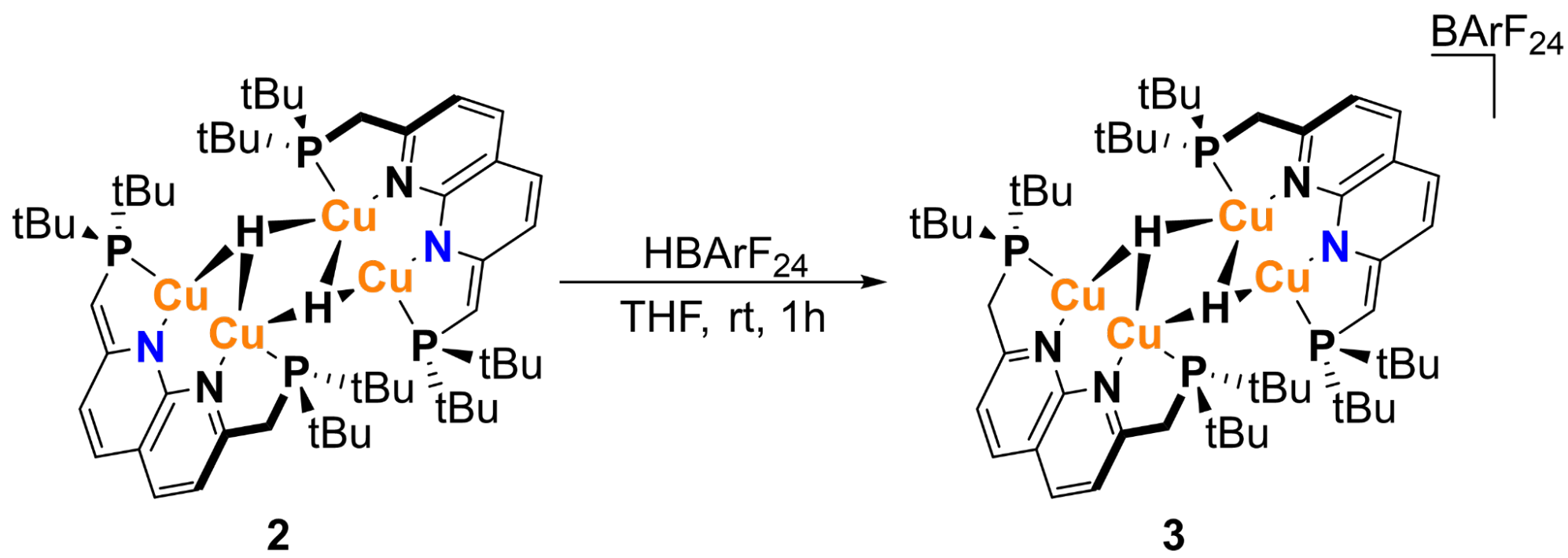

Complex **2** (18.7 mg, 16.3 µmol, 1 eq) was dissolved in THF (3 mL) and to this, freshly dissolved HBArF (16.5 mg, 16.3 µmol, 1 eq) in THF (2 mL) was added dropwise during which the color changed from red to brown/orange. After stirring for 1h, the solvent was removed under dynamic vacuum yielding an orange/brown film. Addition of pentane (1.5 mL) yielded a suspension which was filtered, and the residue was washed with additional pentane (0.5 mL). The solid was extracted with THF (1 mL) and the extract was dried under dynamic vacuum, yielding a film again. The film was stripped with pentane (2 mL) twice, yielding complex **3** (30.3 mg) in 90% yield as an orange powder.

Crystals suitable for single crystal X-ray determination were grown from a saturated benzene solution of **3** (0.5 ml) and pentane (2.5 ml) as anti-solvent.

^{1}H NMR (400 MHz, THF-*d*$_8$, 298 K): δ 8.56 (d, $^{3}J_{H,H}$ = 8.2 Hz, 2H), 7.86 (d, $^{3}J_{H,H}$ = 8.3 Hz, 2H), 7.78 (s, 9H), 7.56 (s, 4H), 7.04 (d, $^{3}J_{H,H}$ = 7.2 Hz, 1H), 6.54 (dd, $^{3}J_{H,H}$ = 9.0, $^{4}J_{H,P}$ = 1.8 Hz, 1H), 6.43 (d, $^{3}J_{H,H}$ = 7.2 Hz, 1H), 6.32 (d, $^{3}J_{H,H}$ = 7.0 Hz, 1H), 3.93 (d, $^{2}J_{H,P}$ = 2.3 Hz, 1H), 3.80 (dd, $^{1}J_{H,H}$ = 17.6 Hz, $^{4}J_{H,P}$ = 8.2 Hz, 2H), 3.71 (bd, $^{1}J_{H,H}$ = 17.6 Hz, 2H), 3.32 (d, $^{2}J_{H,P}$ = 5.0 Hz, 2H), 1.44 – 1.27 (m, 36H), 1.11 (d, $^{3}J_{H,P}$ = 12.8 Hz, 18H), 0.66 (bs, 18H) ppm.

^{31}P{^{1}H} NMR (162 MHz, THF-*d*$_8$, 298 K): δ 41.0 (s), 14.7 (s) ppm.

^{19}F NMR (376 MHz , THF-*d*$_8$, 298 K): δ -63.76 (s) ppm.

^{13}C{^{1}H} APT (101 MHz, THF-*d*$_8$, 298 K): δ 167.9 (d, J = 5.1 Hz), 166.0 (d, J = 16.7 Hz), 163.0 (q, J = 49.7 Hz), 158.2(d, J = 3.3 Hz), 152.1, 141.2, 135.8, 134.8, 130.2 (m), 129.8, 129.7 (d, J = 2.6 Hz), 126.6 (d, J = 9.0 Hz), 125.7 (d, J = 272.8 Hz), 125.3 (d, J = 3.2 Hz), 123.5, 121.6, 119.5 (d, J = 2.24Hz), 118.4 (p, J = 4.4 Hz), 111.6, 81.8 (d, $^{1}J_{C,P}$ = 37.3 Hz), 34.2, 34.1, 33.1(d, $^{1}J_{C,P}$ = 5.4 Hz), 30.9 (d, $^{2}J_{C,P}$ = 7.9 Hz), 30.6 (d, $^{2}J_{C,P}$ = 8.2 Hz), 29.5 (d, $^{2}J_{C,P}$ = 9.2 Hz),

IR-ATR (cm^{-1}): 2946 (m), 1604 (w), 1542 (w), 1501 (w), 1468 (w), 1418 (m), 1353 (m), 1276 (s), 1126 (s), 887 (w), 860 (w), 838 (w), 822 (w), 713 (w), 682 (m).

Anal. Calc. for $C_{84}H_{101}BCu_4F_{24}N_4P_4$ • $(C_5H_{12})_{0.8}$: C, 51.08: H, 5.39; N, 2.71. Found C, 51.15; H, 5.34; N, 2.78. The inclusion of 0.8 eq of pentane provides a satisfactory elemental analysis, which is in line with the presence of small amounts of pentane observed in the ^{1}H NMR spectrum.

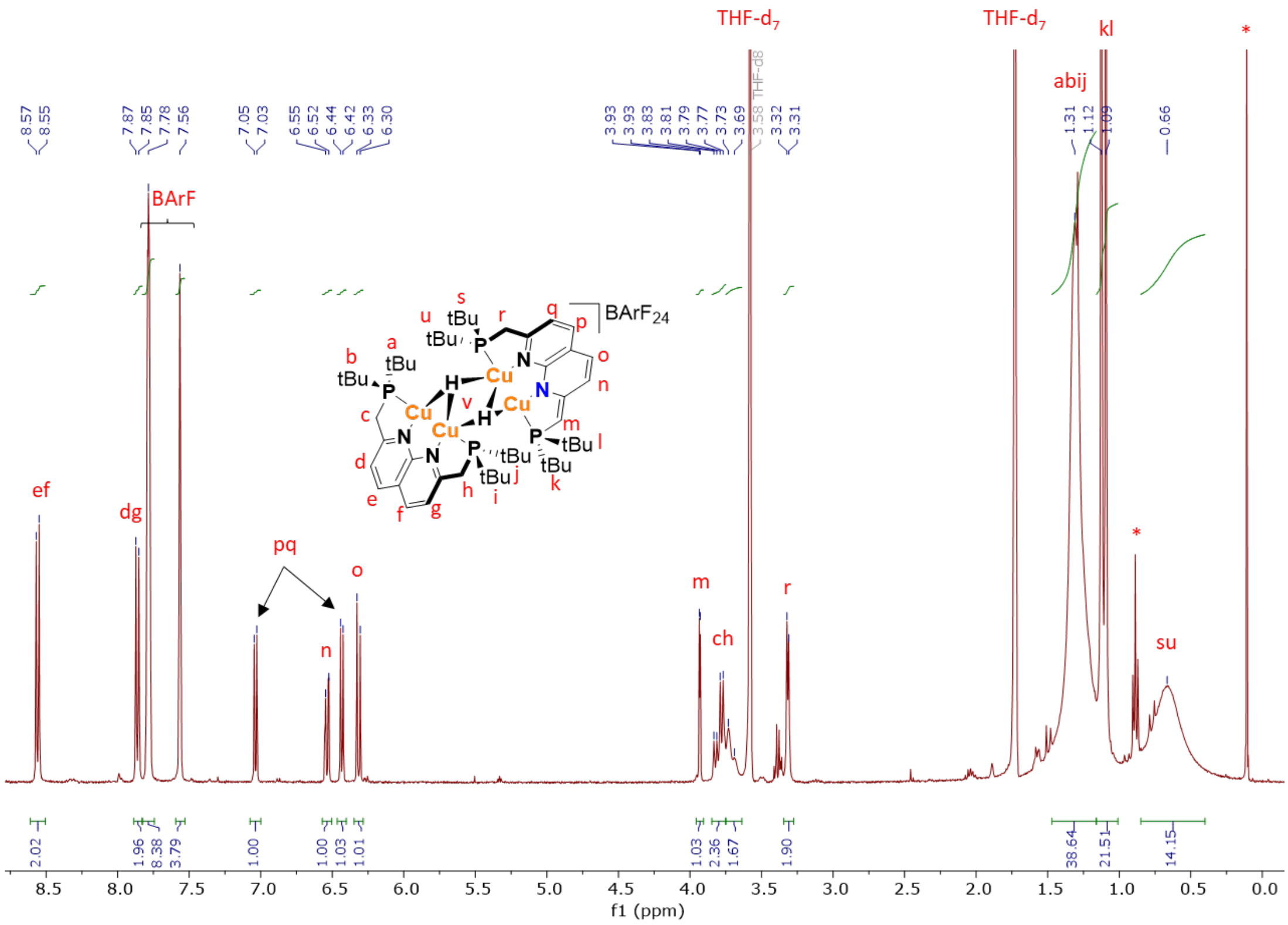

Figure S1. ^{1}H NMR spectrum (THF-d_8, 298 K) of complex **3.** (* denotes grease and traces of pentane)

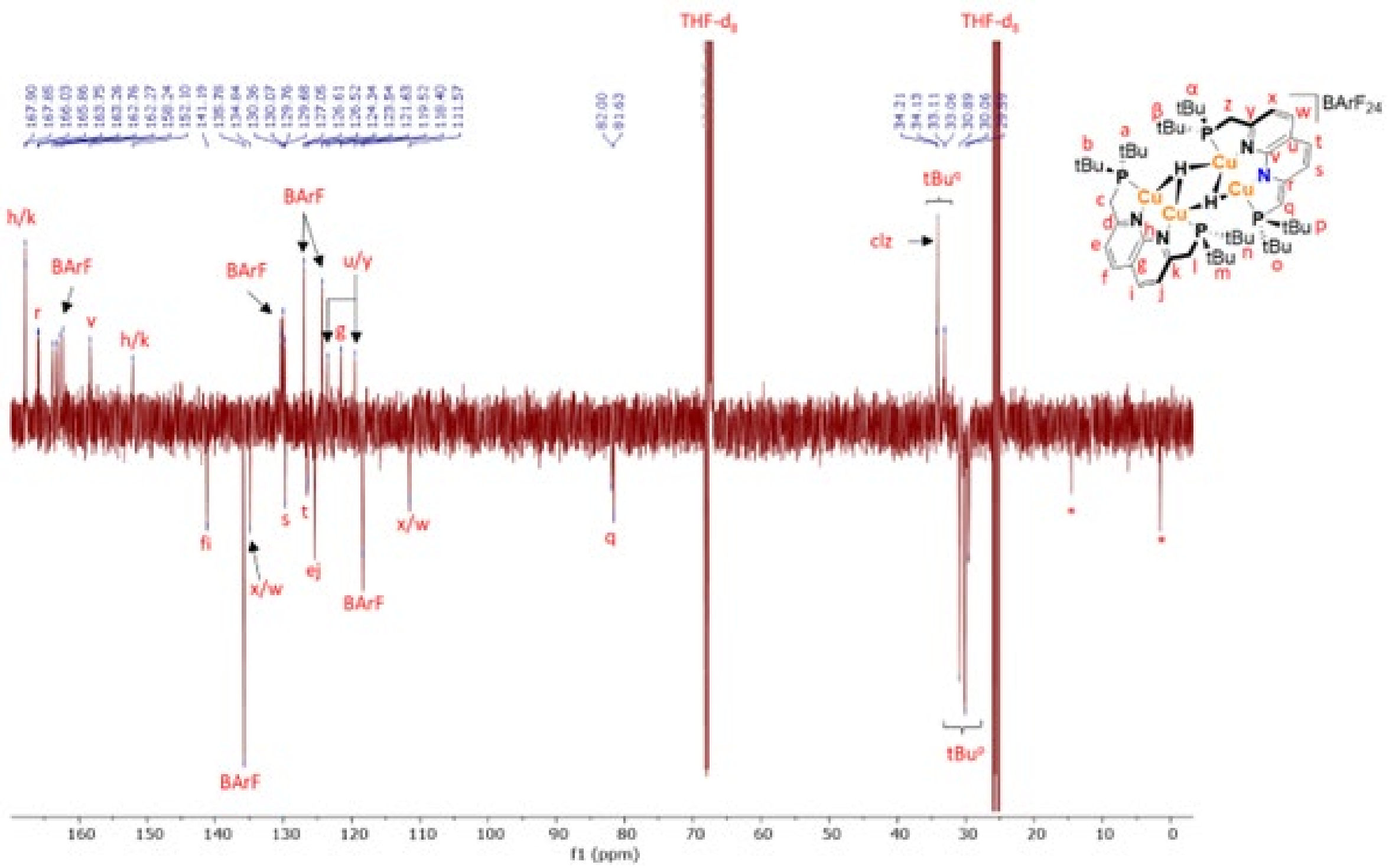

Figure S2. $^{13}C\{^{1}H\}$ APT NMR spectrum (THF-d_8, 298 K) of complex **3.** (* denotes grease and traces of pentane). tBu^p are the primary tBu carbons and tBu^q are the quartenary tBu carbons.

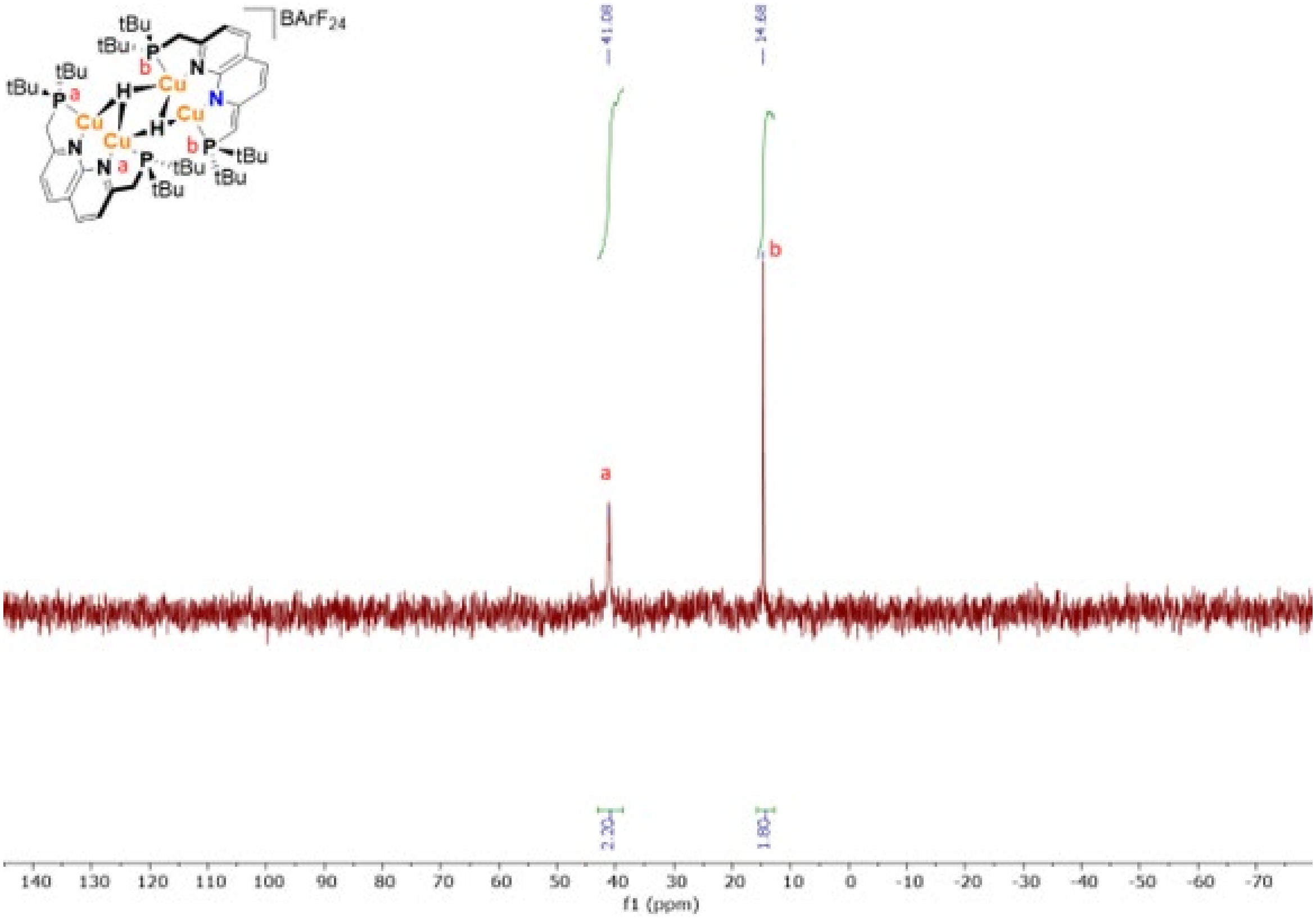

Figure S3. $^{31}P\{^{1}H\}$ NMR spectrum (THF-d$_8$, 298 K) of complex **3.**

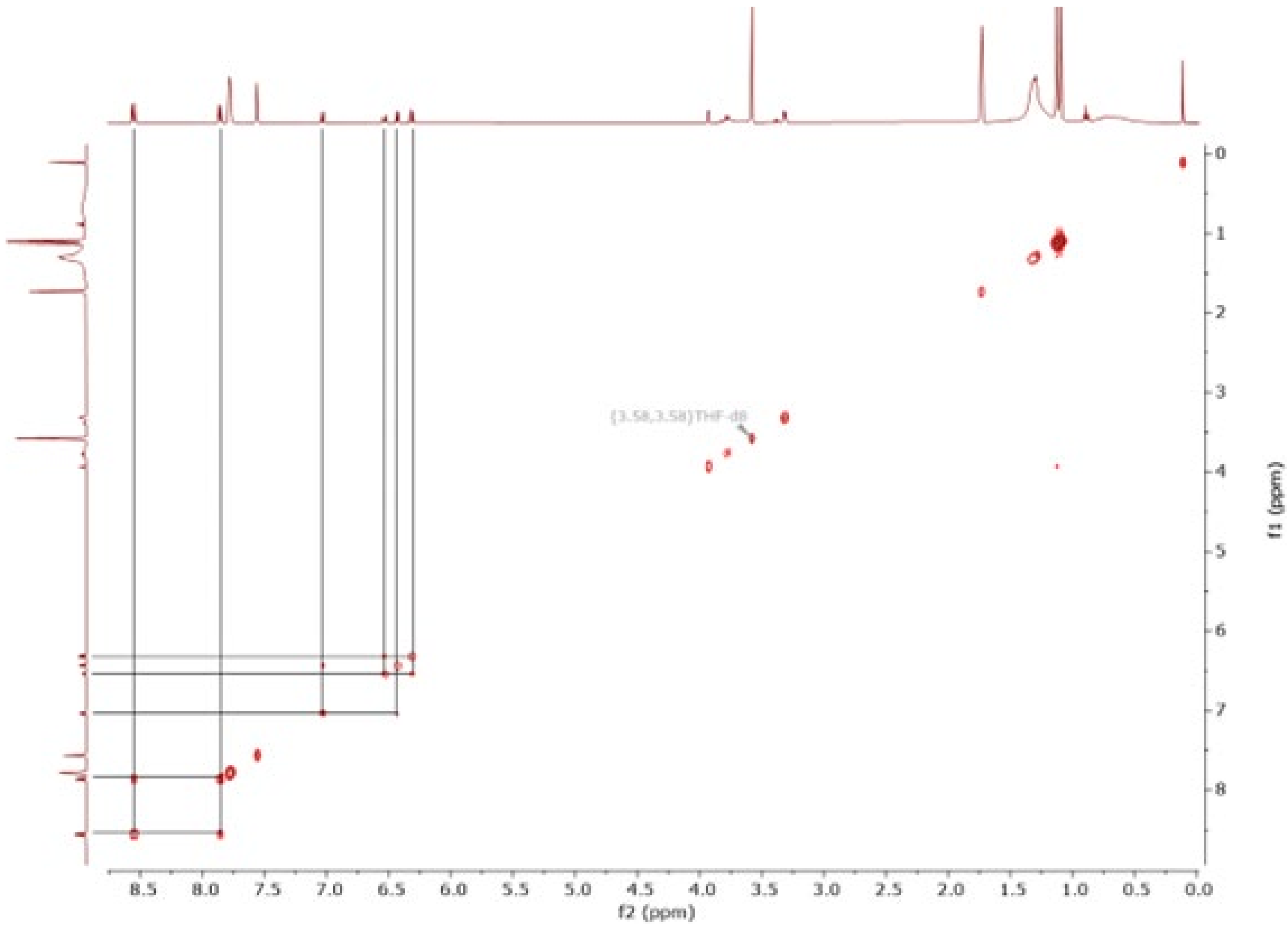

Figure S4. ^{1}H-^{1}H COSY NMR spectrum (THF-d$_8$, 298 K) of complex **3.**

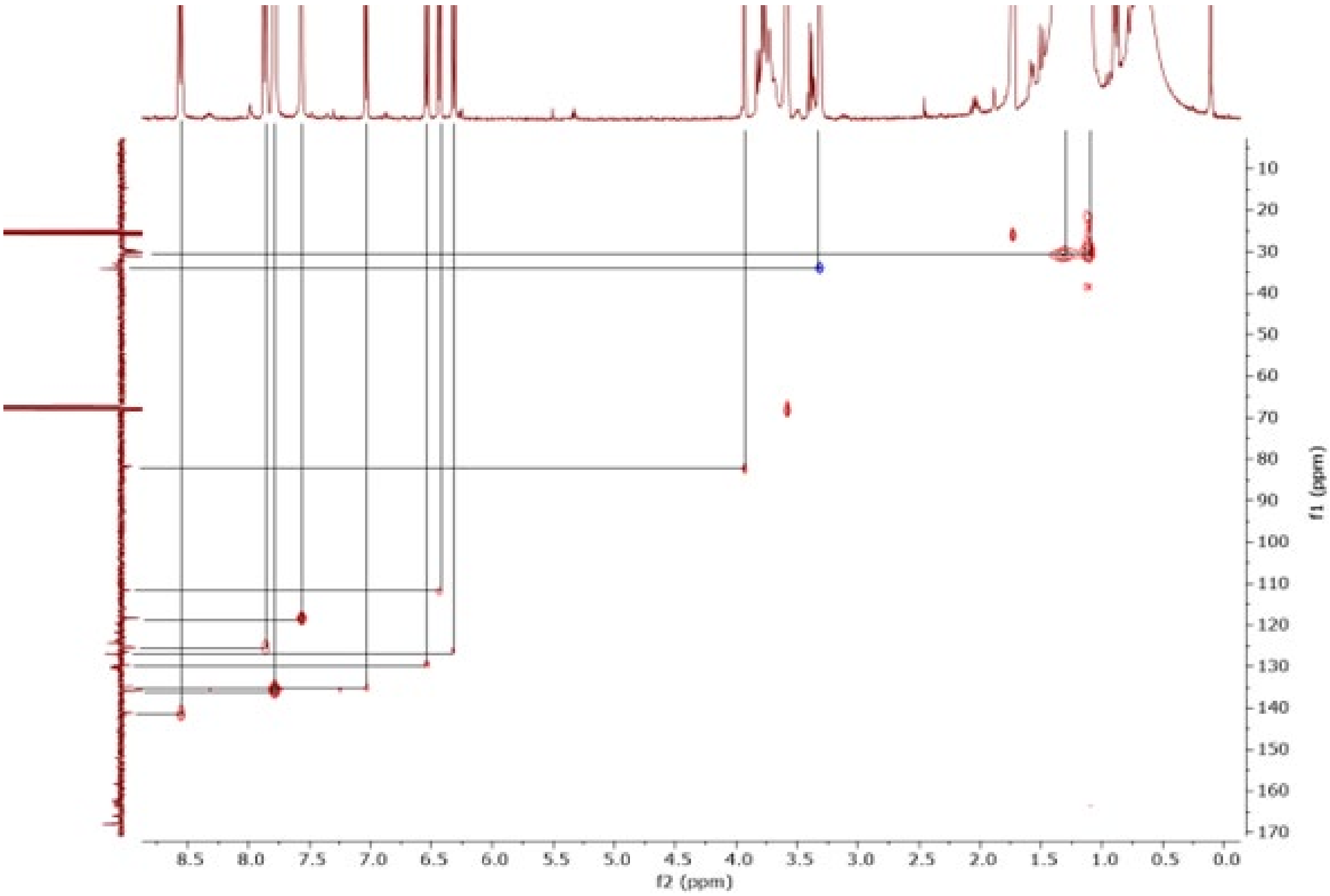

Figure S5. ^{1}H-^{13}C HSQC NMR spectrum (THF-d_8, 298 K) of complex **3.**

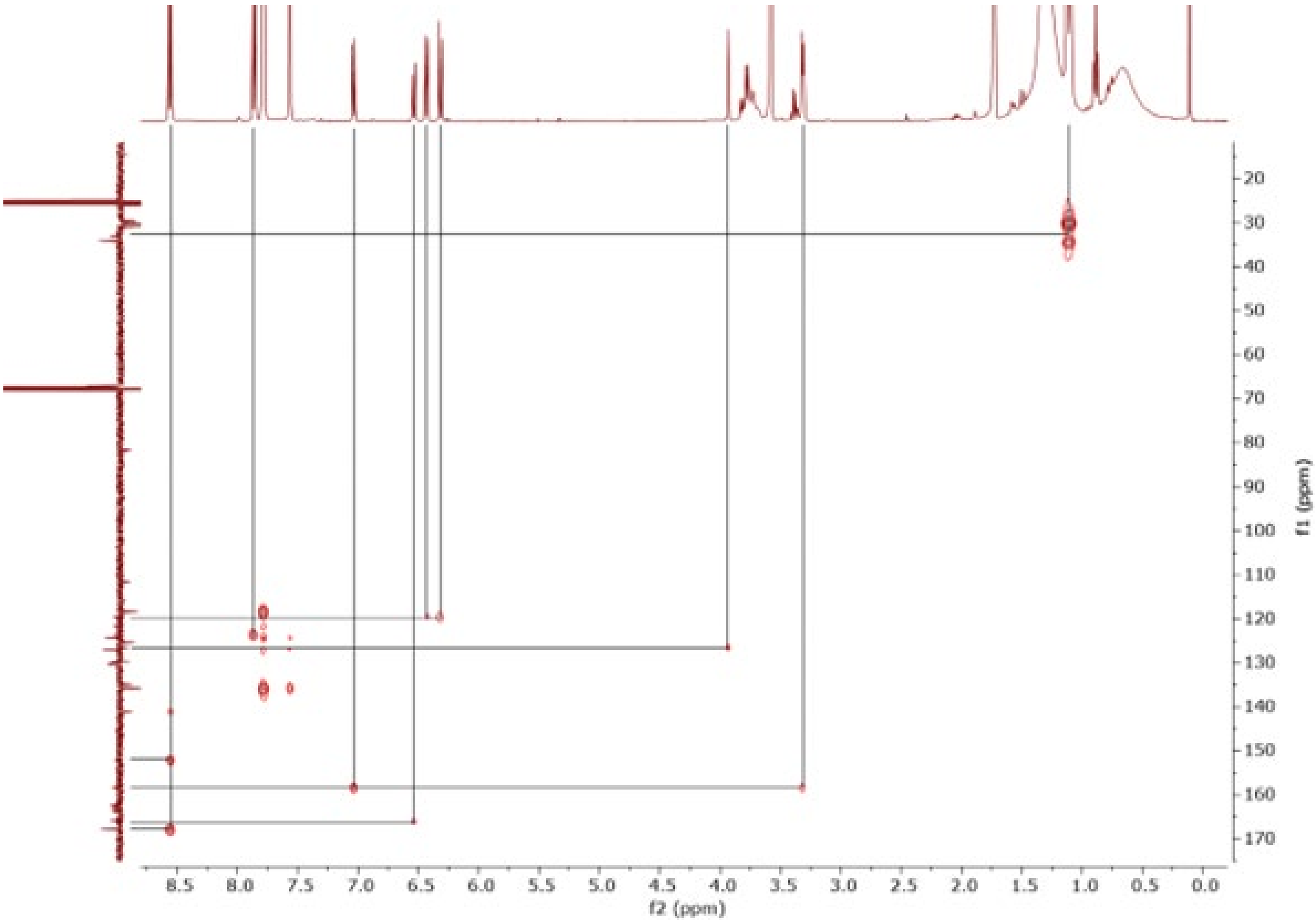

Figure S6. ^{1}H-^{13}C HMBC NMR spectrum (THF-d_8, 298 K) of complex **3.**

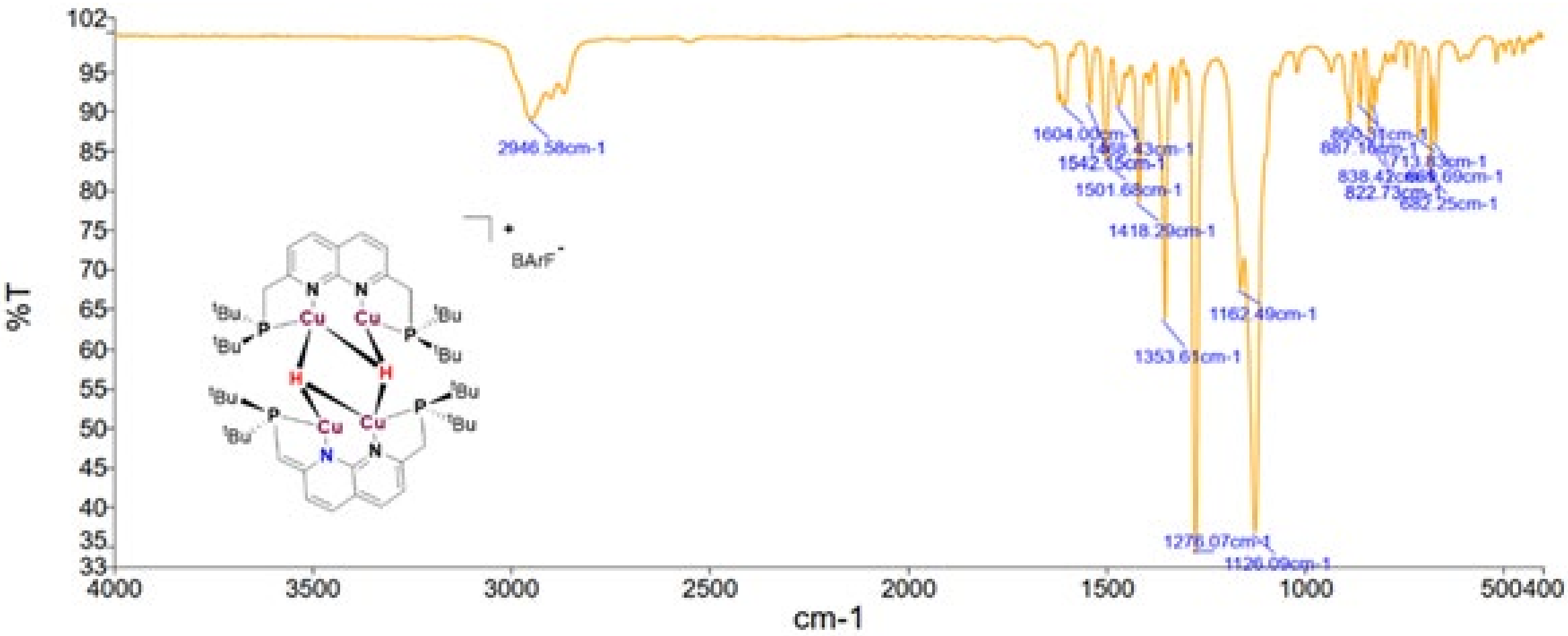

Figure S7. The ATR-IR spectrum of complex **3**.

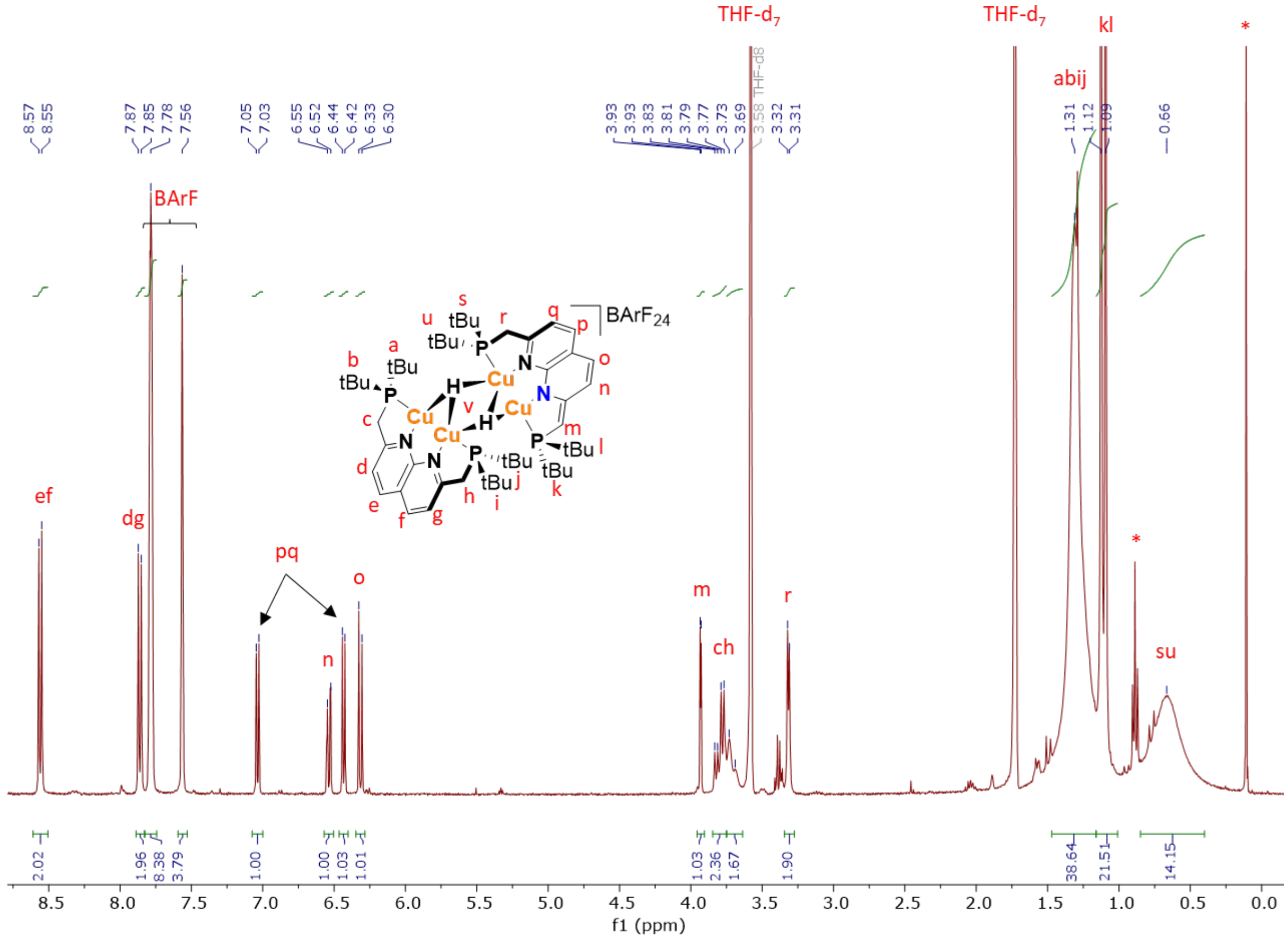

Figure S1. ^{1}H NMR spectrum (THF-d_8, 298 K) of complex **3.** (* denotes grease and traces of pentane)

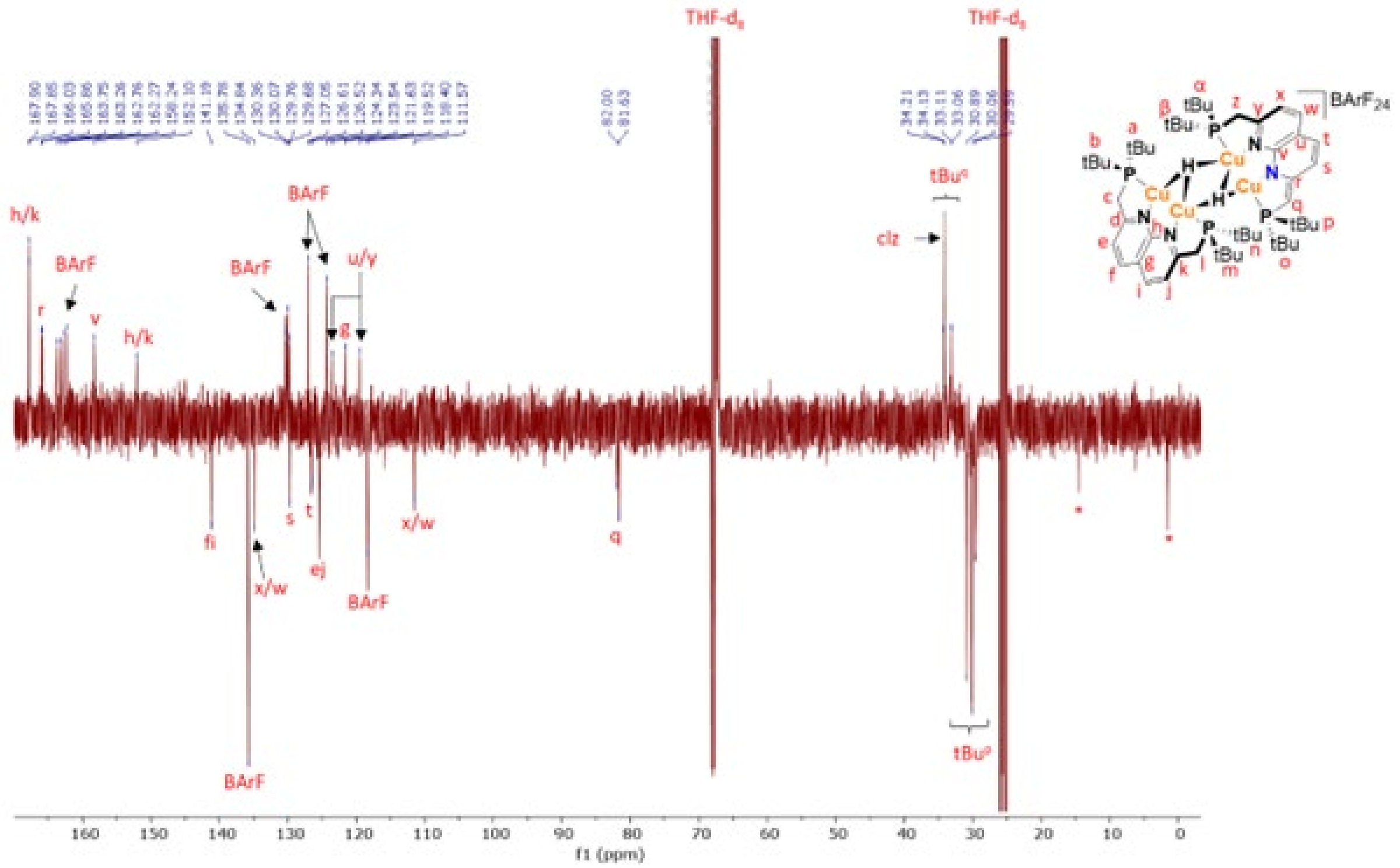

Figure S2. $^{13}C\{^{1}H\}$ APT NMR spectrum (THF-d8, 298 K) of complex **3.** (* denotes grease and traces of pentane). tBu^{p} are the primary tBu carbons and tBu^{q} are the quartenary tBu carbons.

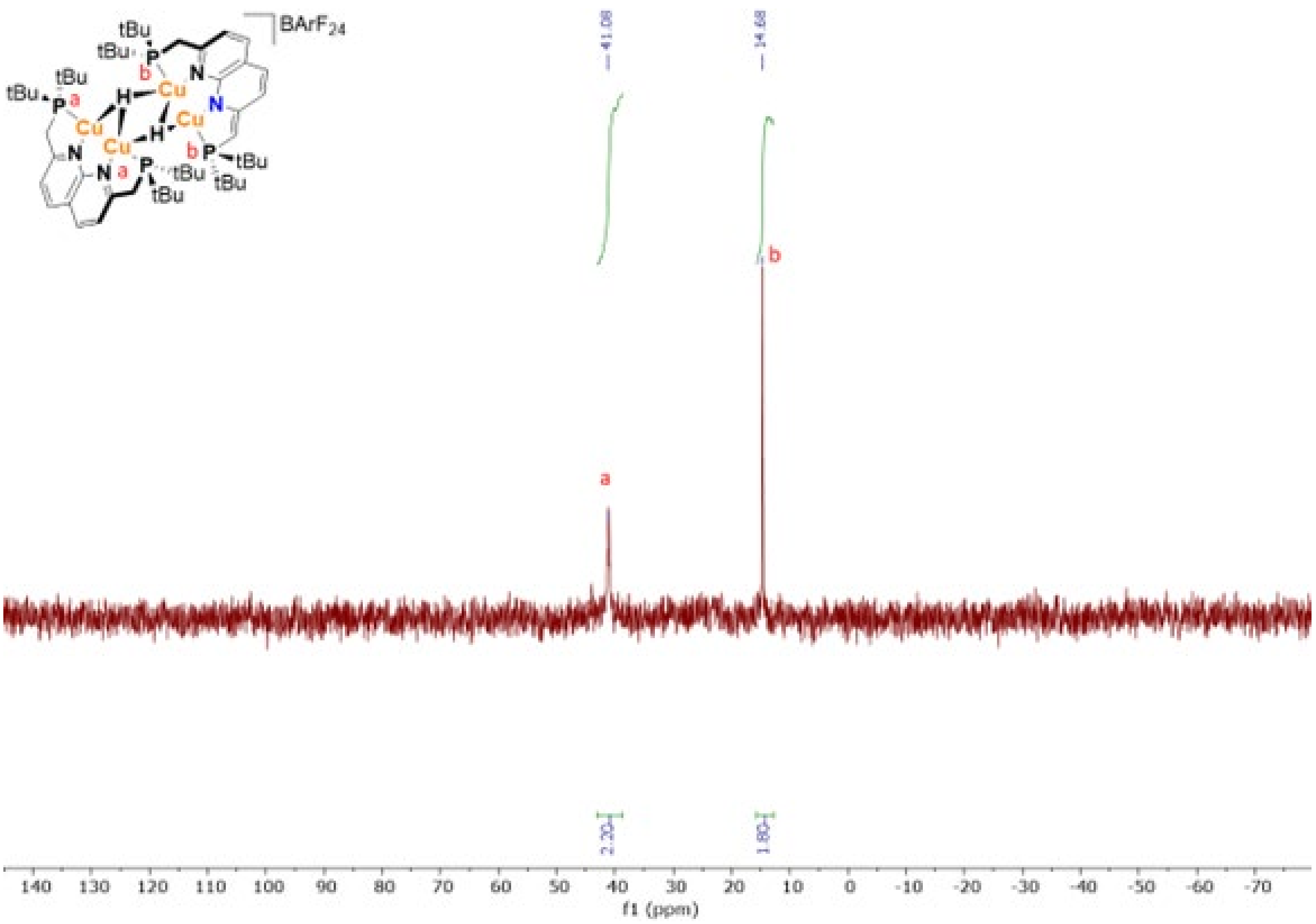

Figure S3. $^{31}P\{^{1}H\}$ NMR spectrum (THF-d8, 298 K) of complex **3.**

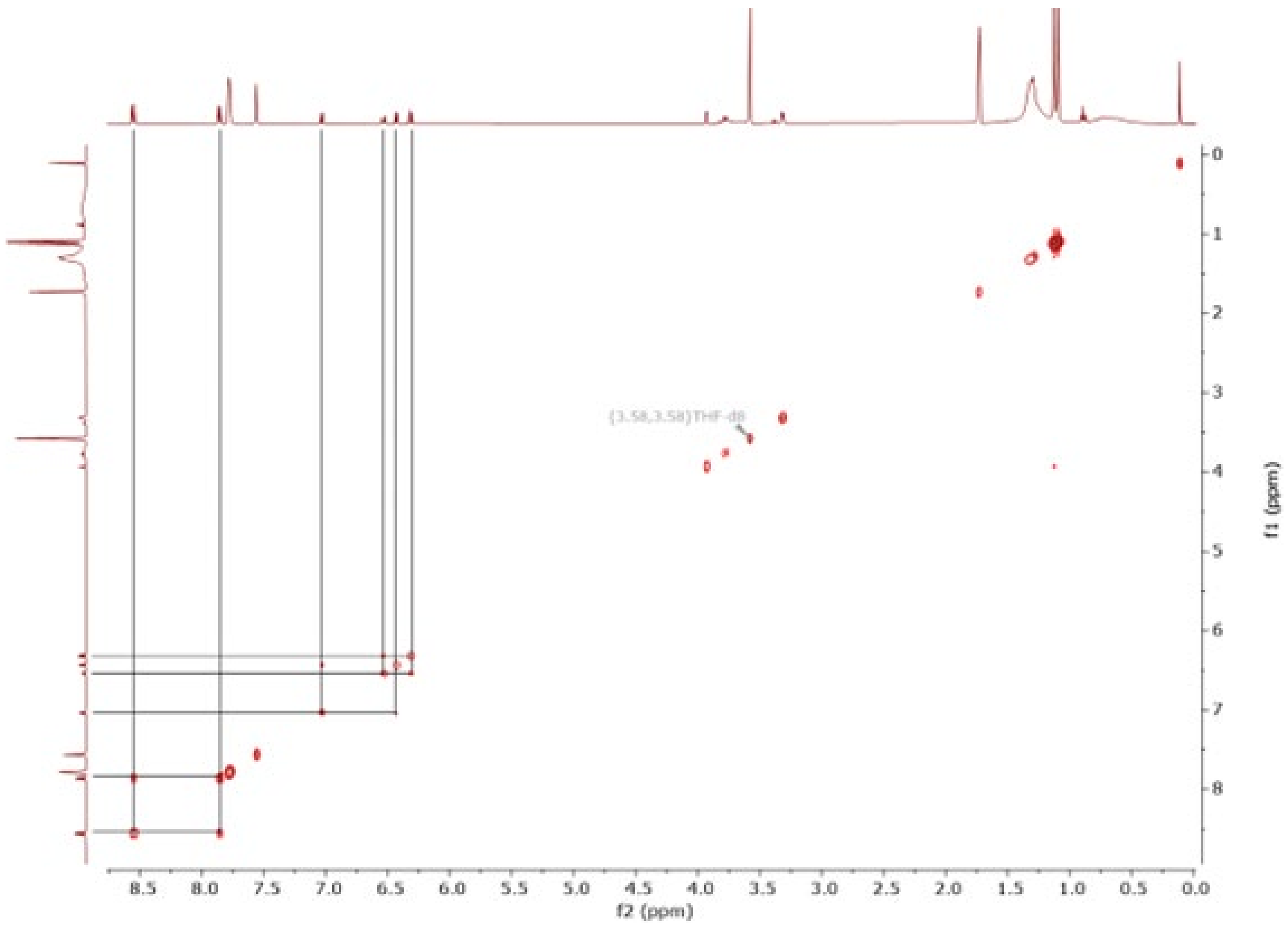

Figure S4. ^{1}H-^{1}H COSY NMR spectrum (THF-d_8, 298 K) of complex **3.**

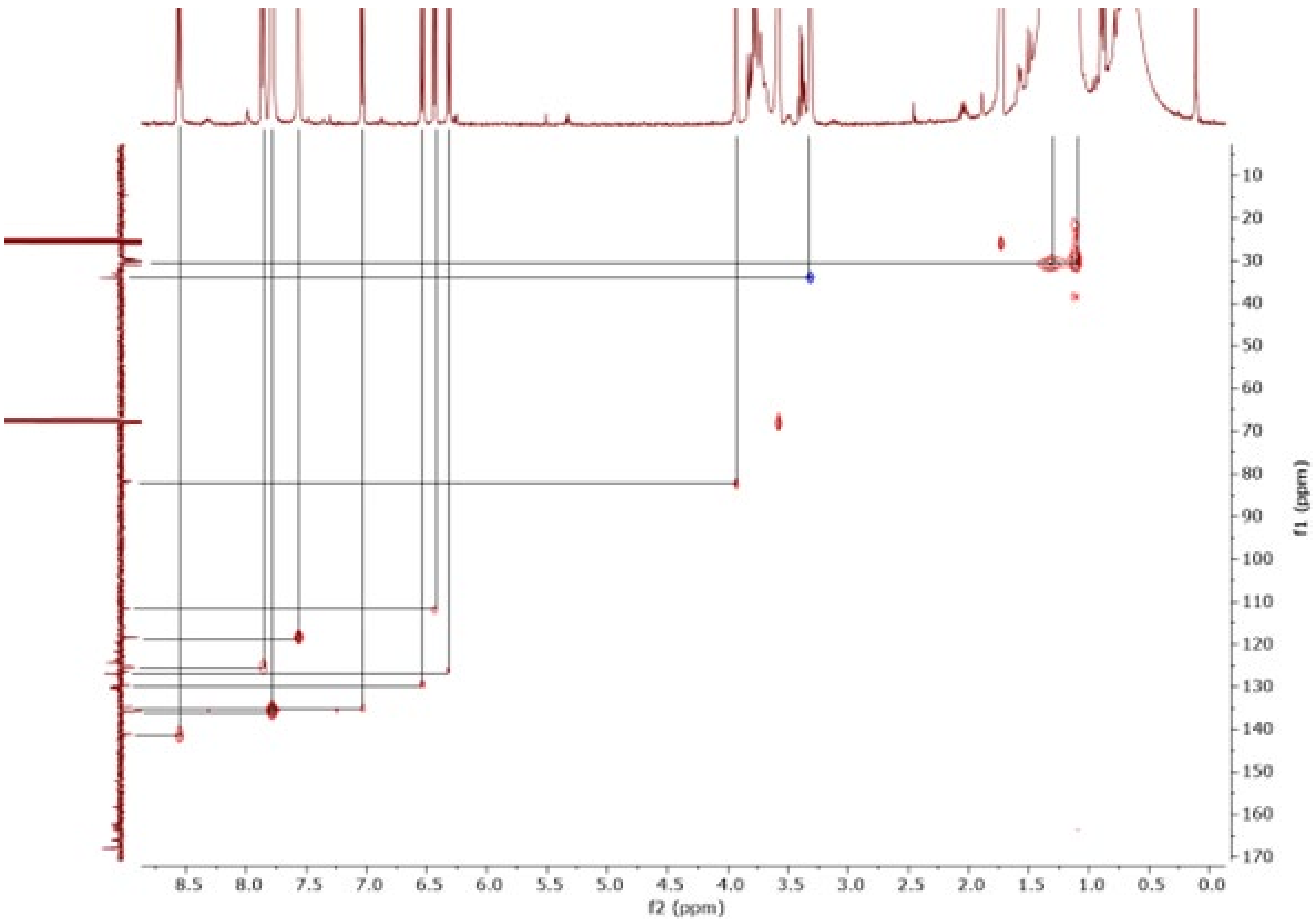

Figure S5. ^{1}H-^{13}C HSQC NMR spectrum (THF-d$_8$, 298 K) of complex **3.**

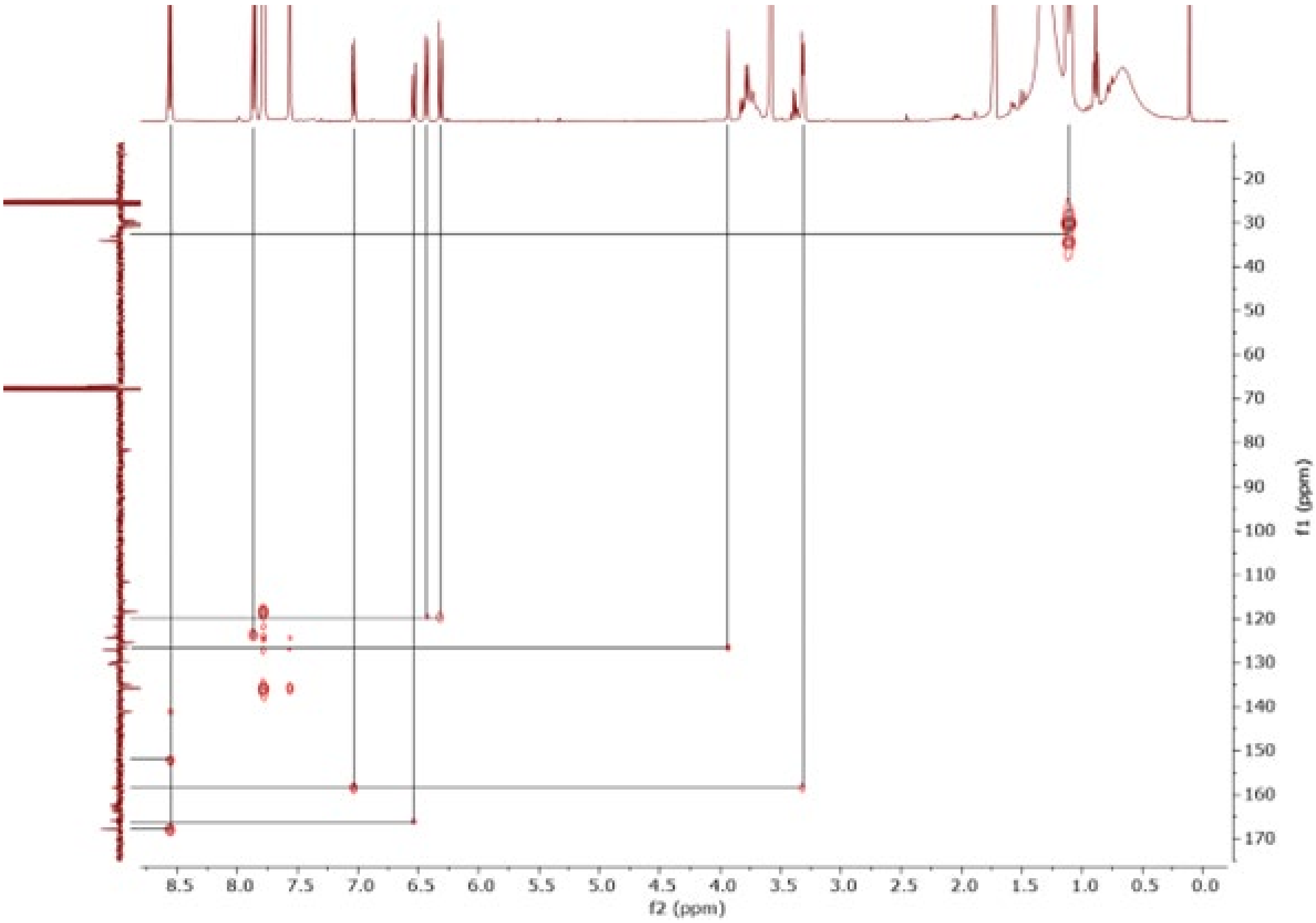

Figure S6. ^{1}H-^{13}C HMBC NMR spectrum (THF-d$_8$, 298 K) of complex **3.**

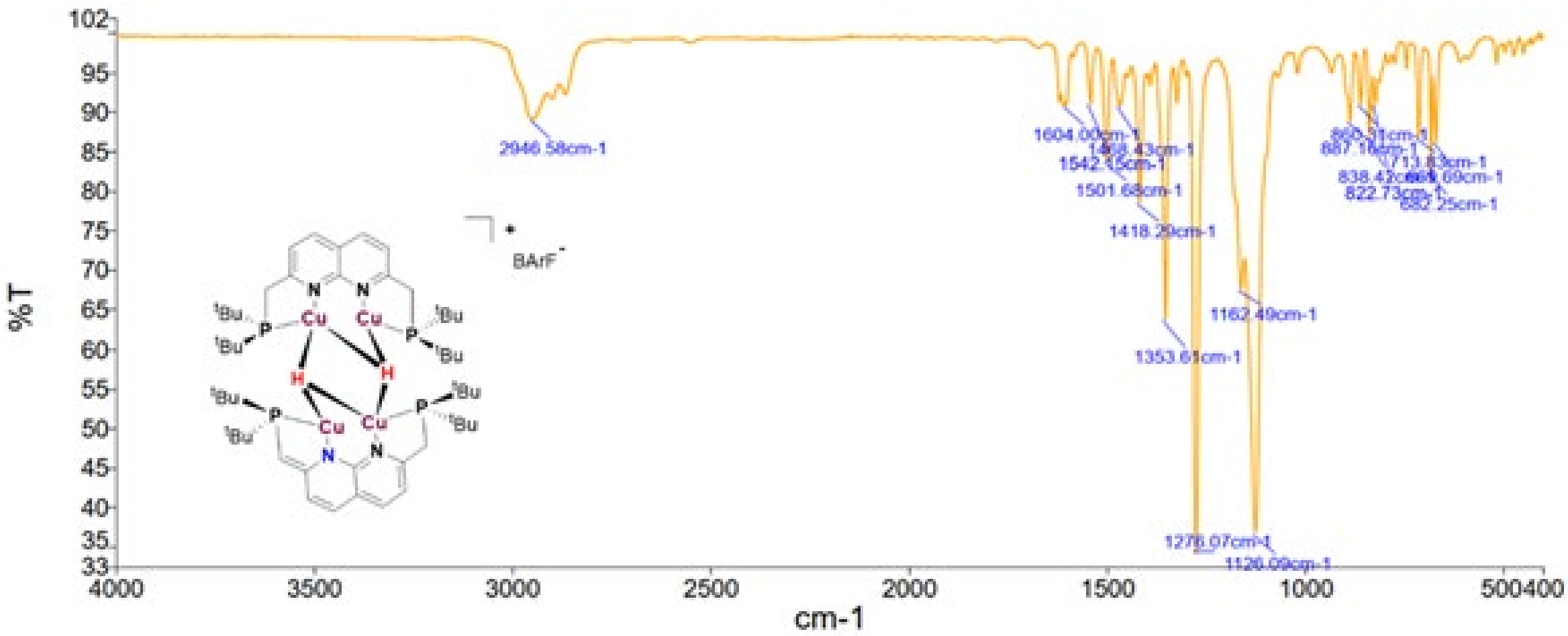

Figure S7. The ATR-IR spectrum of complex **3**.

1.3 Synthesis of complex 4

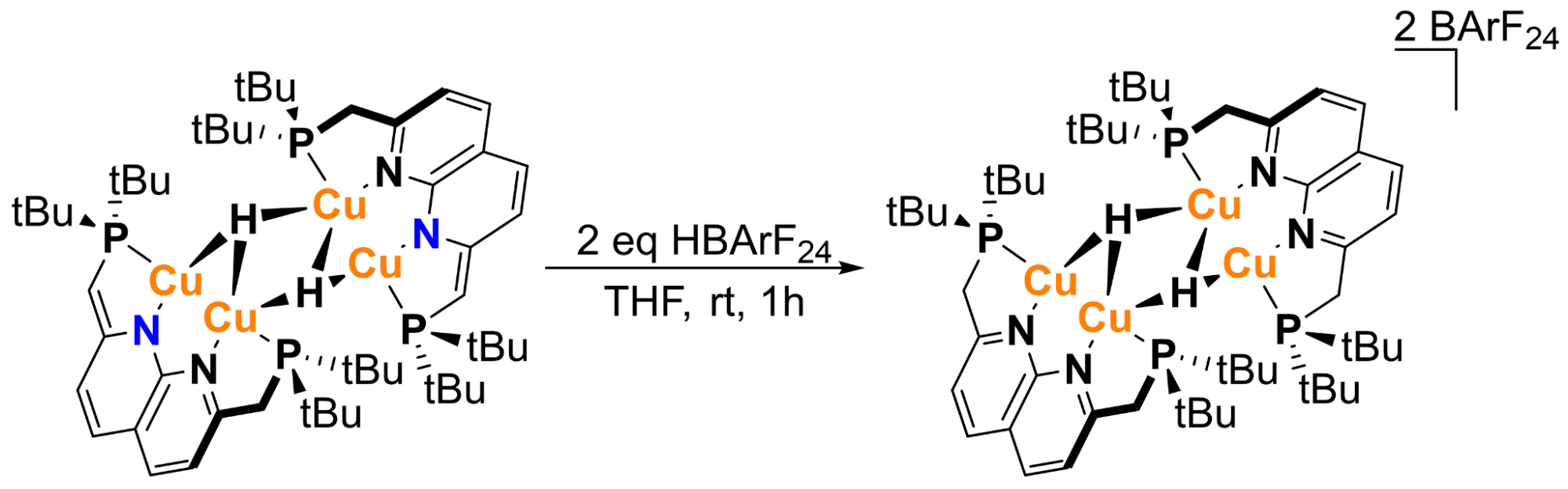

Complex **2** (40.0 mg, 34.86 µmol, 1 eq) was dissolved in THF (4 mL) and to this, a freshly prepared solution of $HBArF_{24}$ (70.84 mg, 71.7 µmol, 2 eq) in THF (4ml) was added dropwise. The reaction mixture was let to stir for one hour, after which the solvent was evaporated yielding a black solid. This solid was washed with pentane (2 mL) yielding complex **4** as a black solid (104.5 mg, 33.7 µmol, 97 %).

Crystals suitable for single crystal X-ray determination were grown from a THF solution of **3** (0.5 ml) and pentane (2.5 ml) as anti-solvent.

^{1}H NMR (400 MHz, THF-*d*₈, 298 K): δ 8.67 (d, $^3J_{H,H}$ = 8.3 Hz, 4H), 7.95 (d, $^3J_{H,H}$ = 8.3 Hz, 4H), 7.78 (s, 16H), 7.56 (s, 8H), 3.77 (d, $^2J_{H,P}$ = 6.5 Hz, 4H), 1.06 (d, $^3J_{H,P}$ = 13.3 Hz, 72H) ppm.

^{31}P{^{1}H} NMR (162 MHz, THF-d_8, 298 K): δ 37.43 (bs) ppm.

^{19}F NMR (376 MHz ,THF-d_8, 298 K): δ -63.76 (s) ppm.

^{13}C{^{1}H} APT (101 MHz, THF-d_8, 298 K): δ 168.4 (d, J = 4.6 Hz), 162.9 (q, J = 49.5 Hz), 151.8, 142.1, 135.7, 130.1 (m), 125.7 (d, J = 2.3 Hz), 125.6 (d, J = 272.5 Hz), 118.3 (quint, J = 3.8 Hz), 33.9 (d, $^1J_{C,P}$ = 12.9 Hz), 33.5 (d, $^1J_{C,P}$ = 5.5 Hz), 29.8 (d, $^2J_{C,P}$ = 7.7 Hz) ppm.

IR-ATR (cm^{-1}): 2959 (m), 1604 (m), 1509, 1353 (s), 1275 (s), 1123 (S), 887 (w), 862 (w), 839 (m), 713 (m), 682 (m), 670 (m).

Anal. Calc. for $C_{116}H_{114}B_2Cu_4F_{48}N_4P_4$: C, 48.45; H, 4.00; N, 1.95. Found C, 49.70; H, 4.39; N, 2.05. We were unable to obtain a satisfactory elemental analysis for **4**, likely due to the inclusion of solvent (THF and pentane) in the final product. However, due to the broad peaks from traces of **3** around 0.8-1.5 ppm in ^{1}H NMR, we cannot accurately assess if and how much pentane is present.

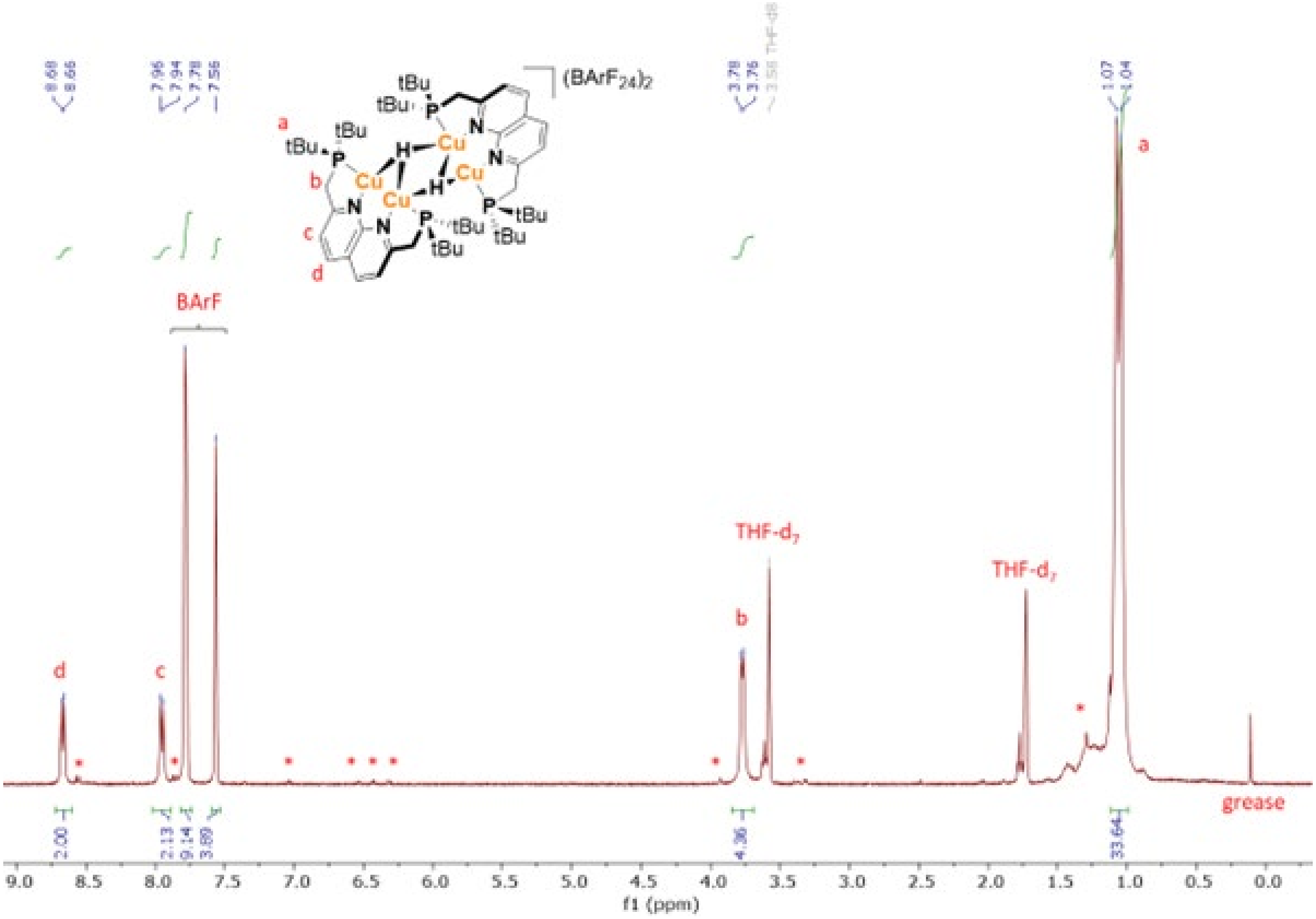

Figure S8. ^{1}H NMR spectrum (THF-d_8, 298 K) of complex **4.** (* denotes traces of complex **3**)

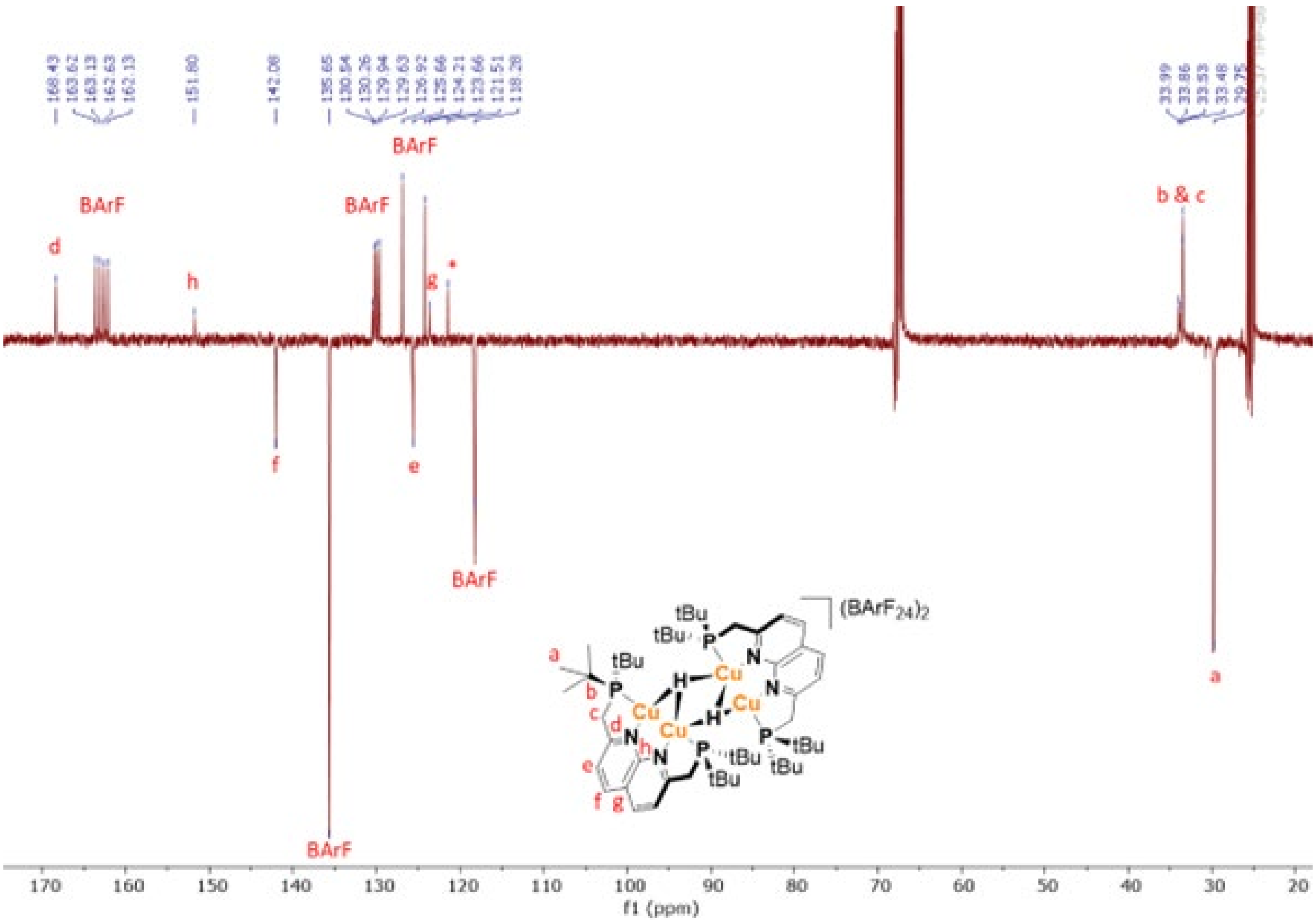

Figure S9. $^{13}C\{^{1}H\}$ APT NMR spectrum (THF-d_8, 298 K) of complex **4.** (* denotes an unassigned peak).

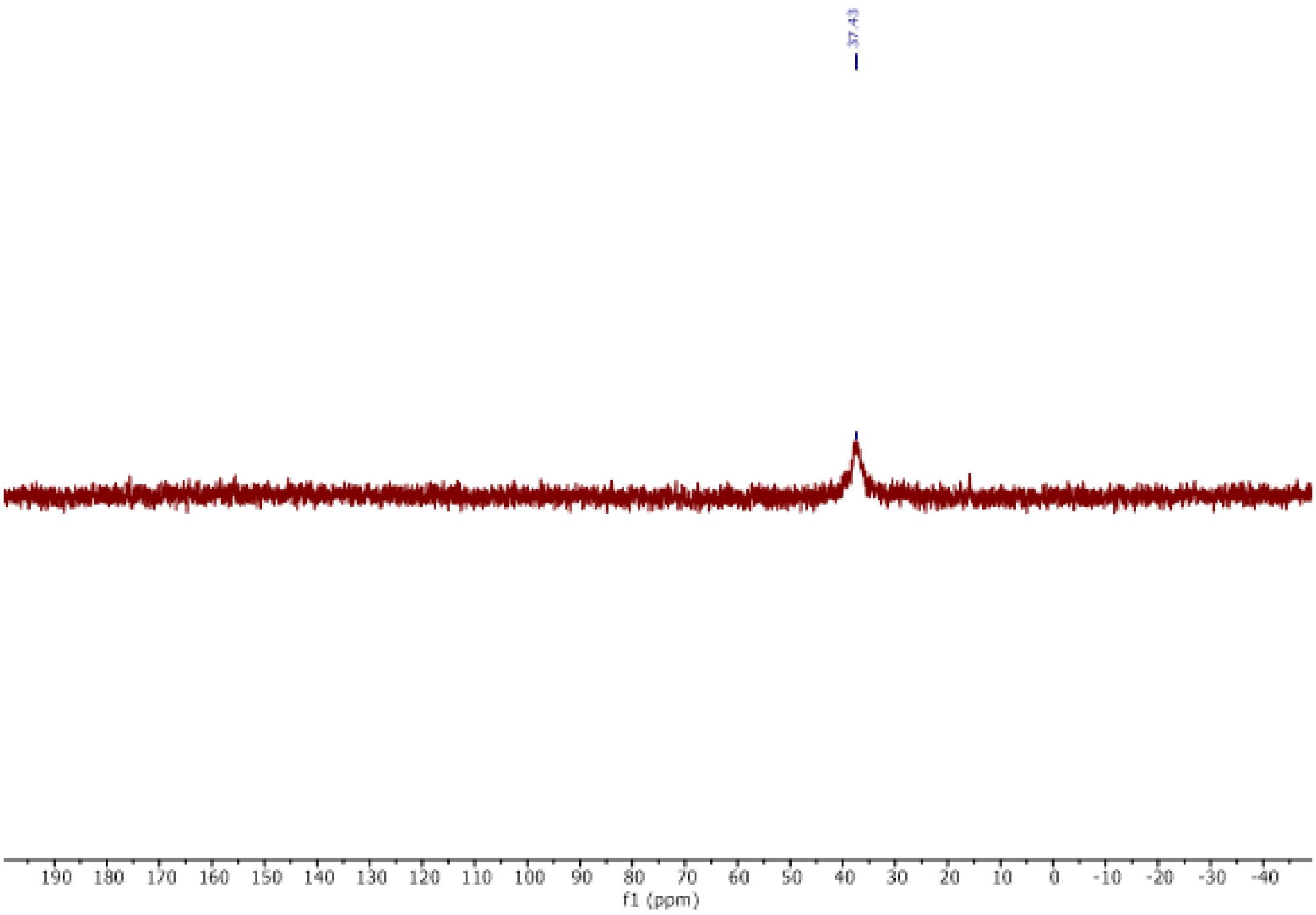

Figure S10. $^{31}P\{^{1}H\}$ NMR spectrum (THF-d_8, 298 K) of complex **4.**

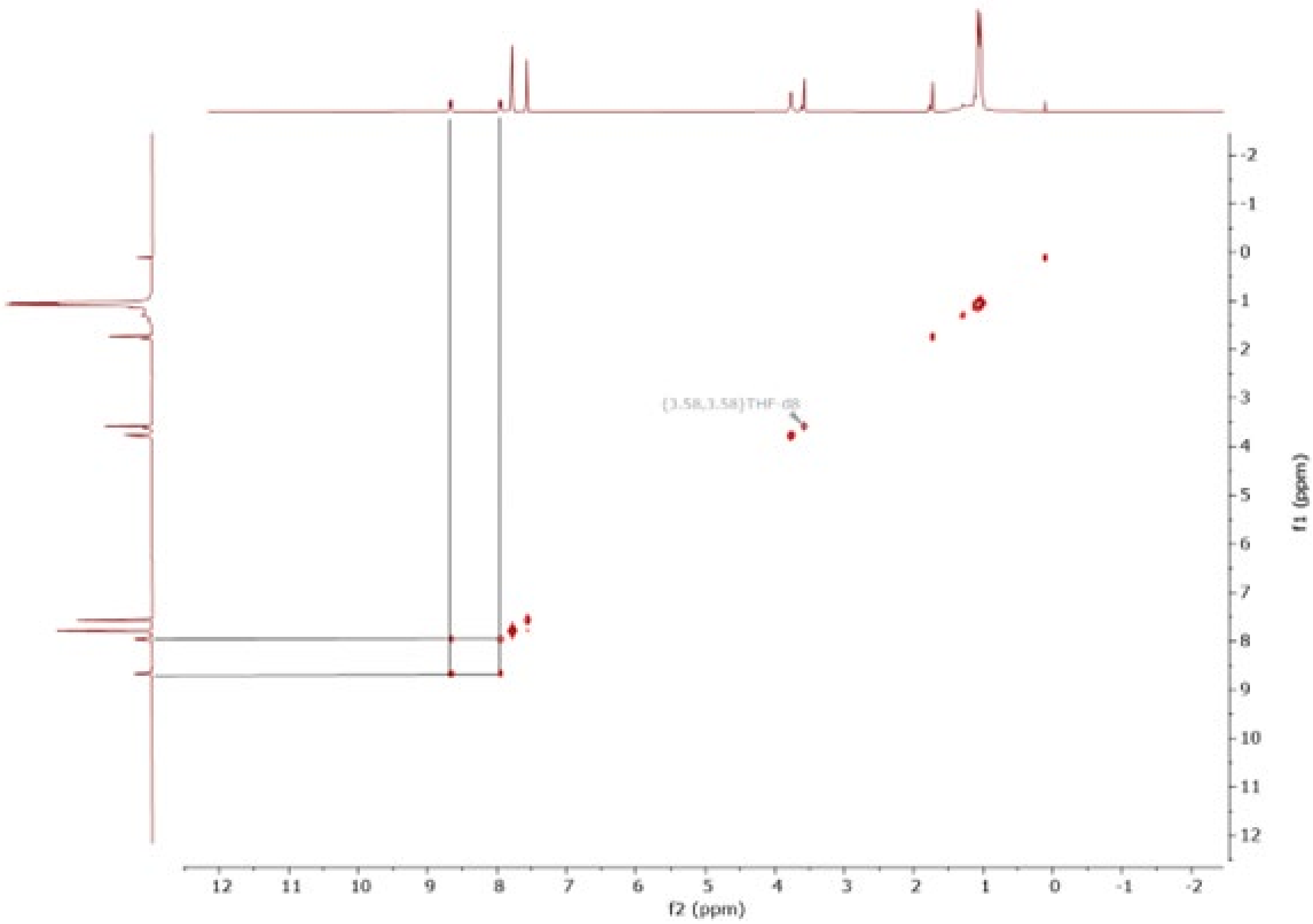

Figure S11. ^{1}H-^{1}H COSY NMR spectrum (THF-d_8, 298 K) of complex **4.**

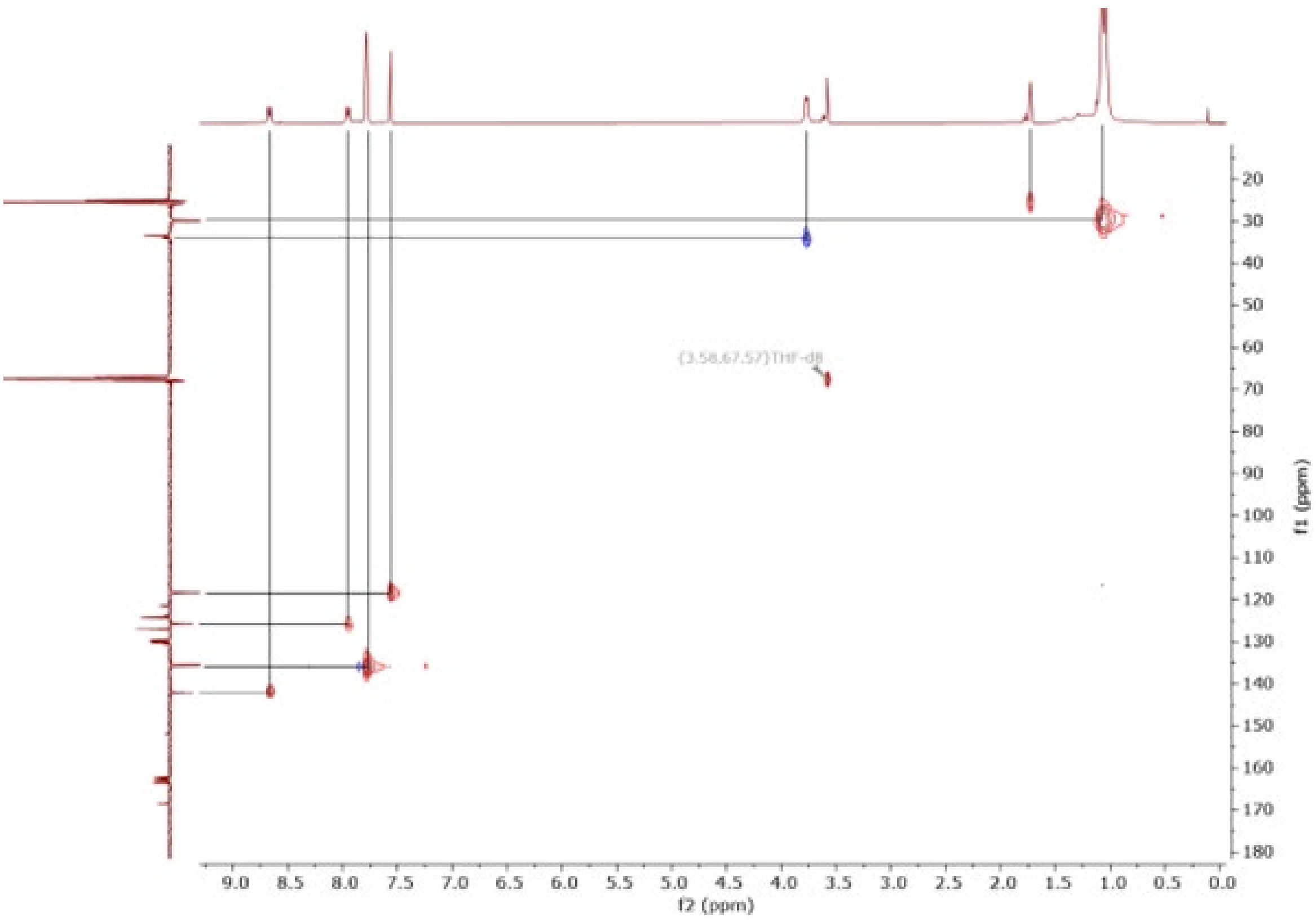

Figure S12. ^{1}H-^{13}C HSQC NMR spectrum (THF-d_8, 298 K) of complex **4.**

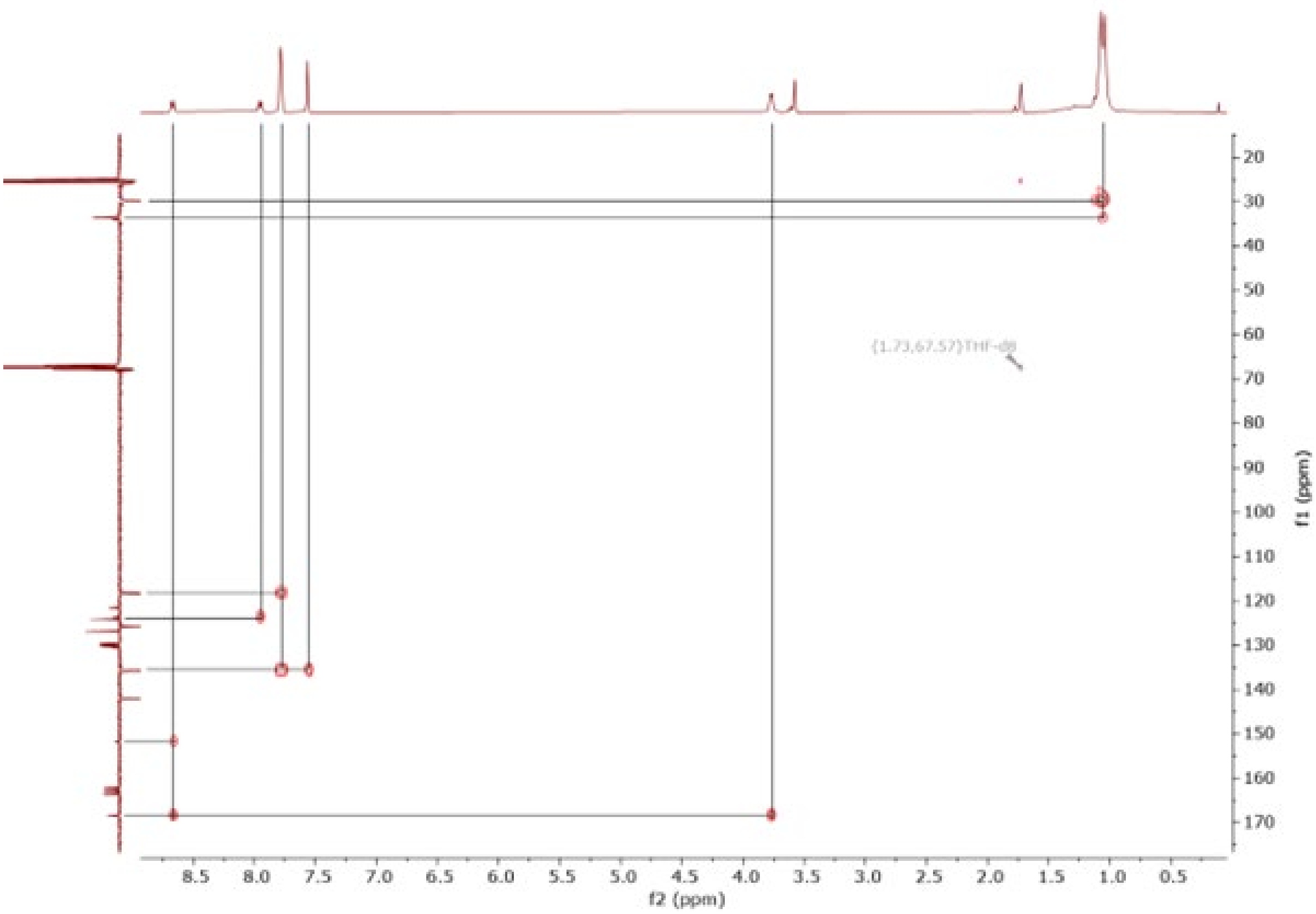

Figure S13. ^{1}H-^{13}C HMBC NMR spectrum (THF-d_8, 298 K) of complex **4**.

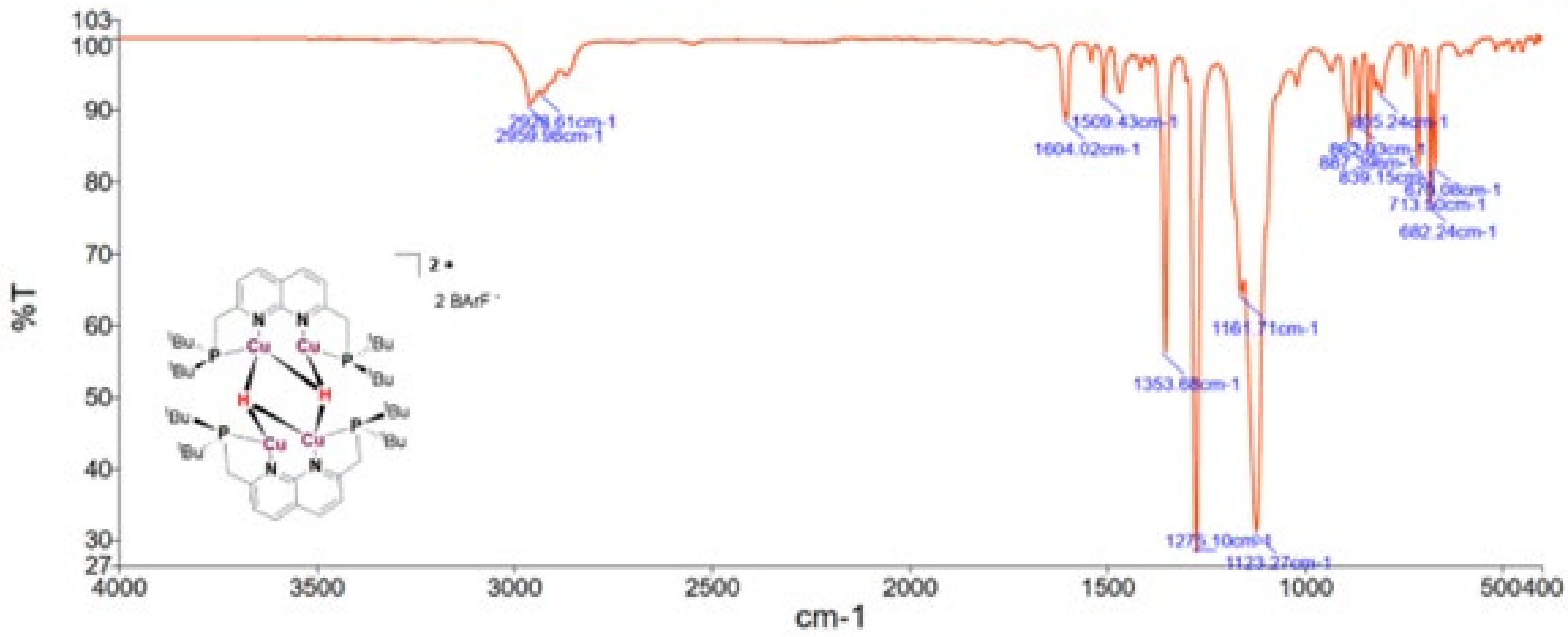

Figure S14. The ATR-IR spectrum of complex **4**.

1.4 Synthesis of the deuterated analogue of complex 3 (3D) *in situ*

The deuterated analogue of complex **2** (**2D**) was prepared according to literature procedure[1] and without isolating the product, HBArF (24.7 mg, 24.4 µmol, 1.05 eq) freshly dissolved in THF (0.25 mL) was added to the solution of **2D** (23.2 µmol, 1 eq) in THF (1 mL). This mixture was analyzed without purification using ^{2}H NMR.

^{2}H NMR (61 MHz, THF-h_8): δ 1.27 (s) ppm.

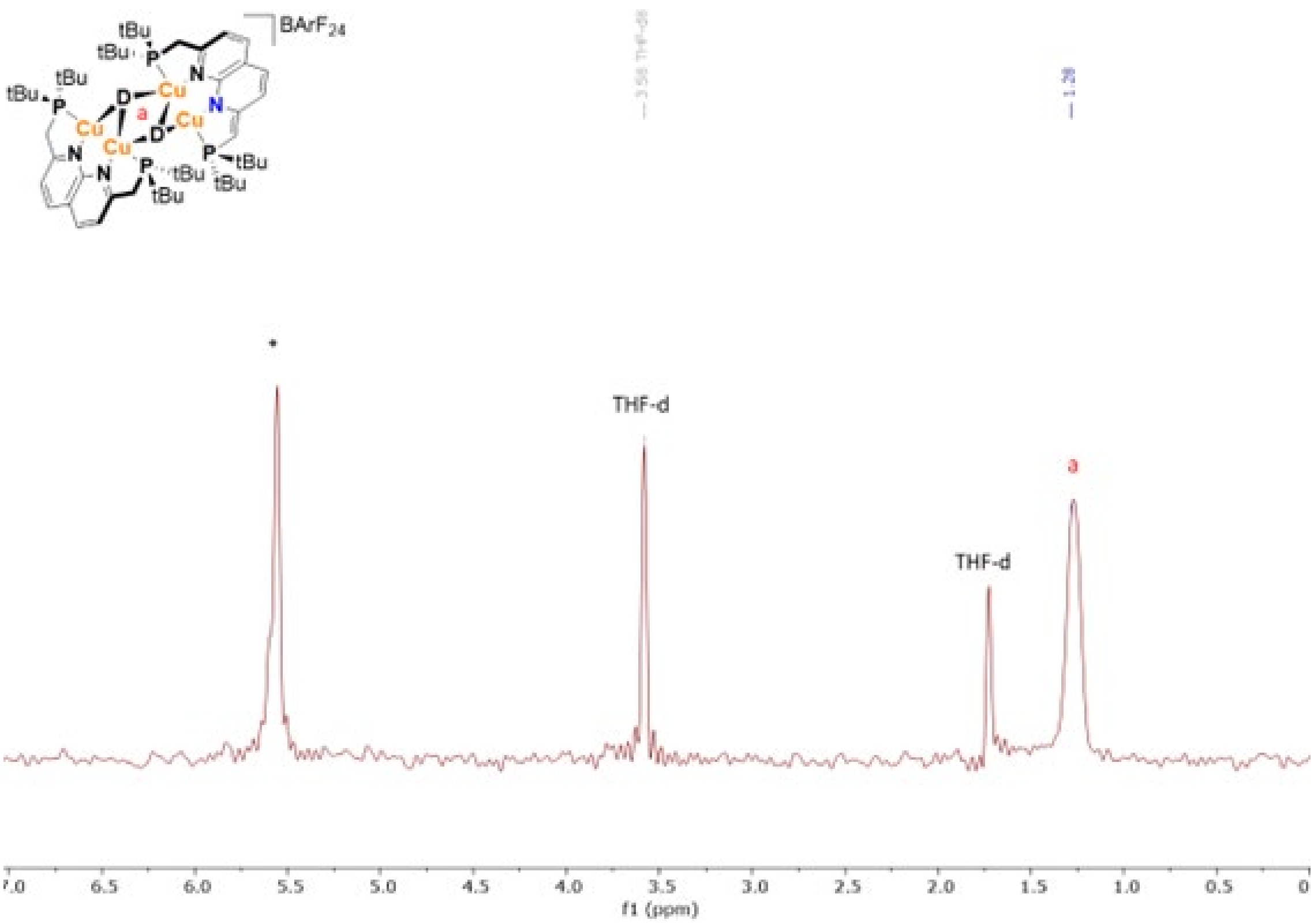

Figure S15. The ^{2}H NMR spectrum (THF-h_8, 298 K) of the deuterated analogue of **3** measured in situ. (* denotes the $DSiPh_2OtBu$ byproduct generated in the synthesis of **2D**)

1.5 Synthesis of the deuterated analogue of complex 4 (4D) *in situ*

To a solution of **3D** (11.1 µmol) in THF (0.6 mL) prepared as described above, HBArF (11.6 mg, 11.5 µmol, 1.03 eq) freshly dissolved in THF (0.1 mL) was added. This mixture was analyzed without purification using ^{2}H NMR.

^{2}H NMR (61 MHz, THF-h_8): δ 1.47 (s) ppm.

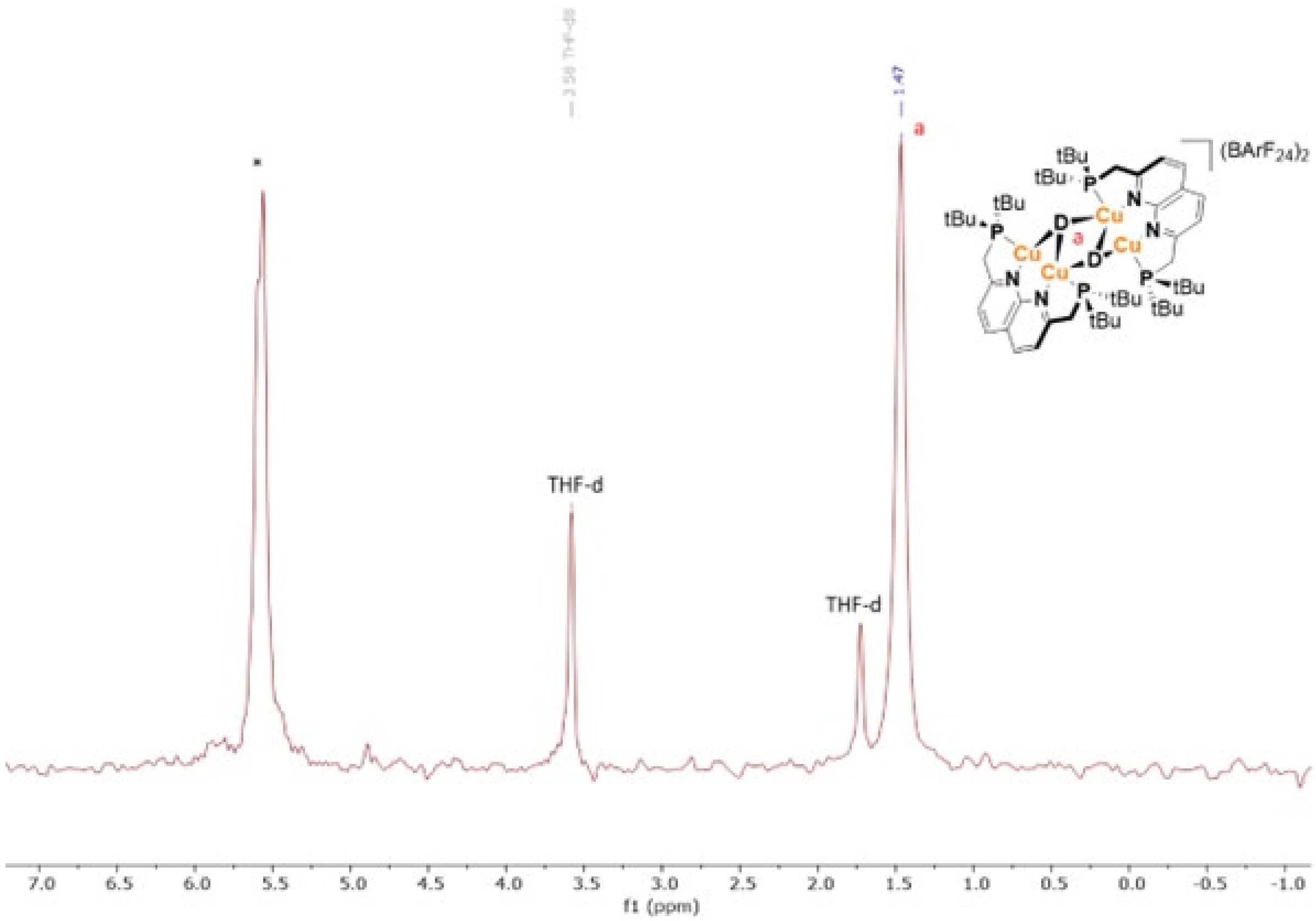

Figure S16. The ^{2}H NMR spectrum (THF-h_8, 298 K) of the deuterated analogue of **4** measured in situ. (* denotes the $DSiPh_2OtBu$ byproduct generated in the synthesis of **2D**)

1.6 DOSY NMR

In the DOSY NMR spectra, we observe that **3** has a larger diffusion constant than **4**, which we hypothesize is due to the co-diffusion of the $BArF_{24}$ anions, however, the DOSY peak fitting procedure is not able to distinguish two diffusion constants for these anions because they overlap and are hence fitted as one peak. It is, however, important to establish that in **3**, the $BArF_{24}$ anions also co-diffuse in order to establish the monomeric or dimeric state of **4** in solution. To verify that this is the case, DOSY was also measured on a mixture of **2** and **3**, in which case the same trend was observed (i.e. **2** has a larger diffusion constant than **3**). Since the symmetry of the ^{1}H NMR spectra of **2** and **3** show that they are both dimers, this difference shows that also in the case of **3**, the anions co-diffuse. Therefore, we can conclude that **4** is a dimer in solution.

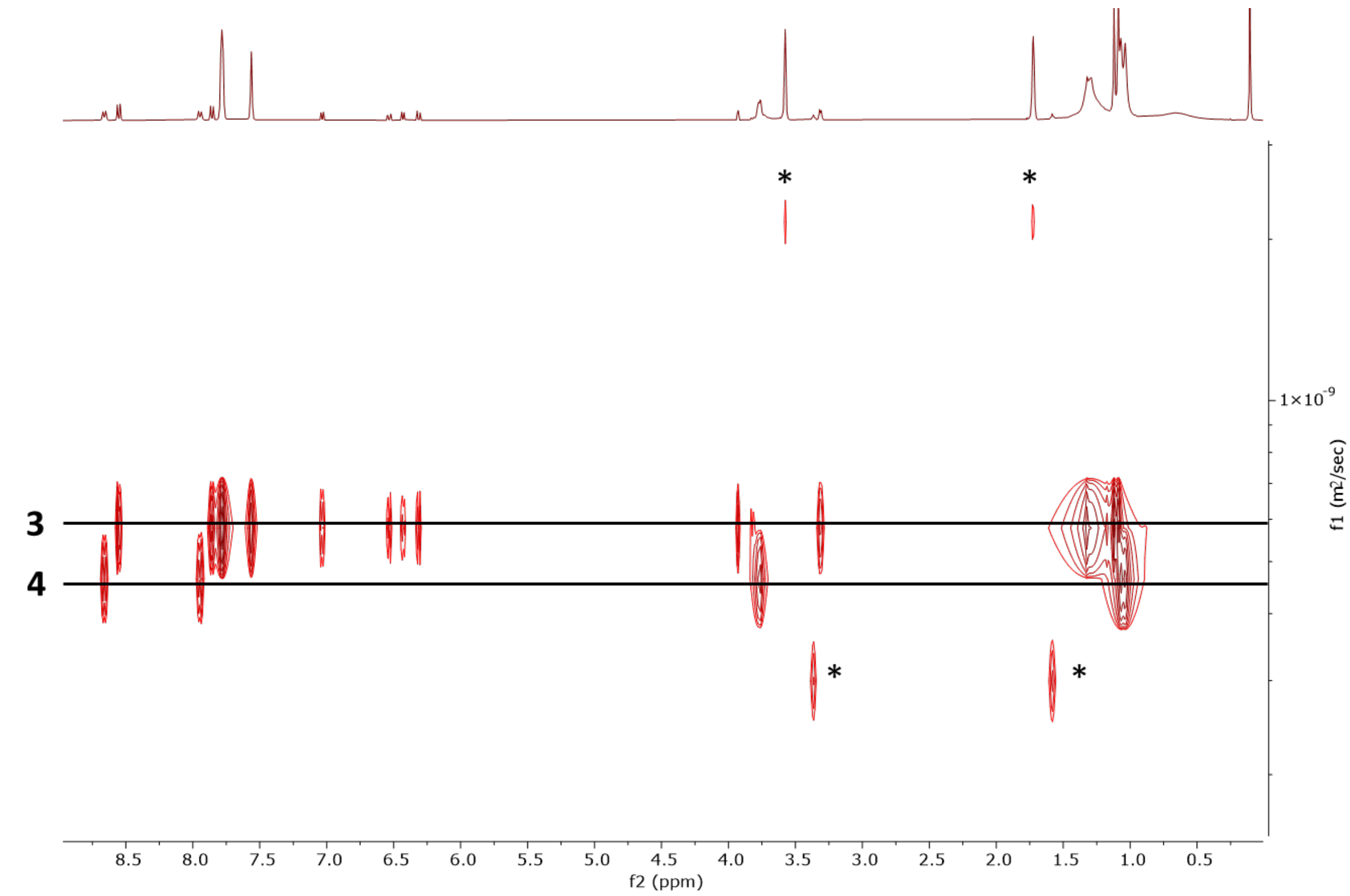

Figure S17. The ^{1}H DOSY NMR spectrum (THF-d_8, 298 K) of a mixture of **3** and **4**. *denotes THF-d_7 and traces of poly-THF.

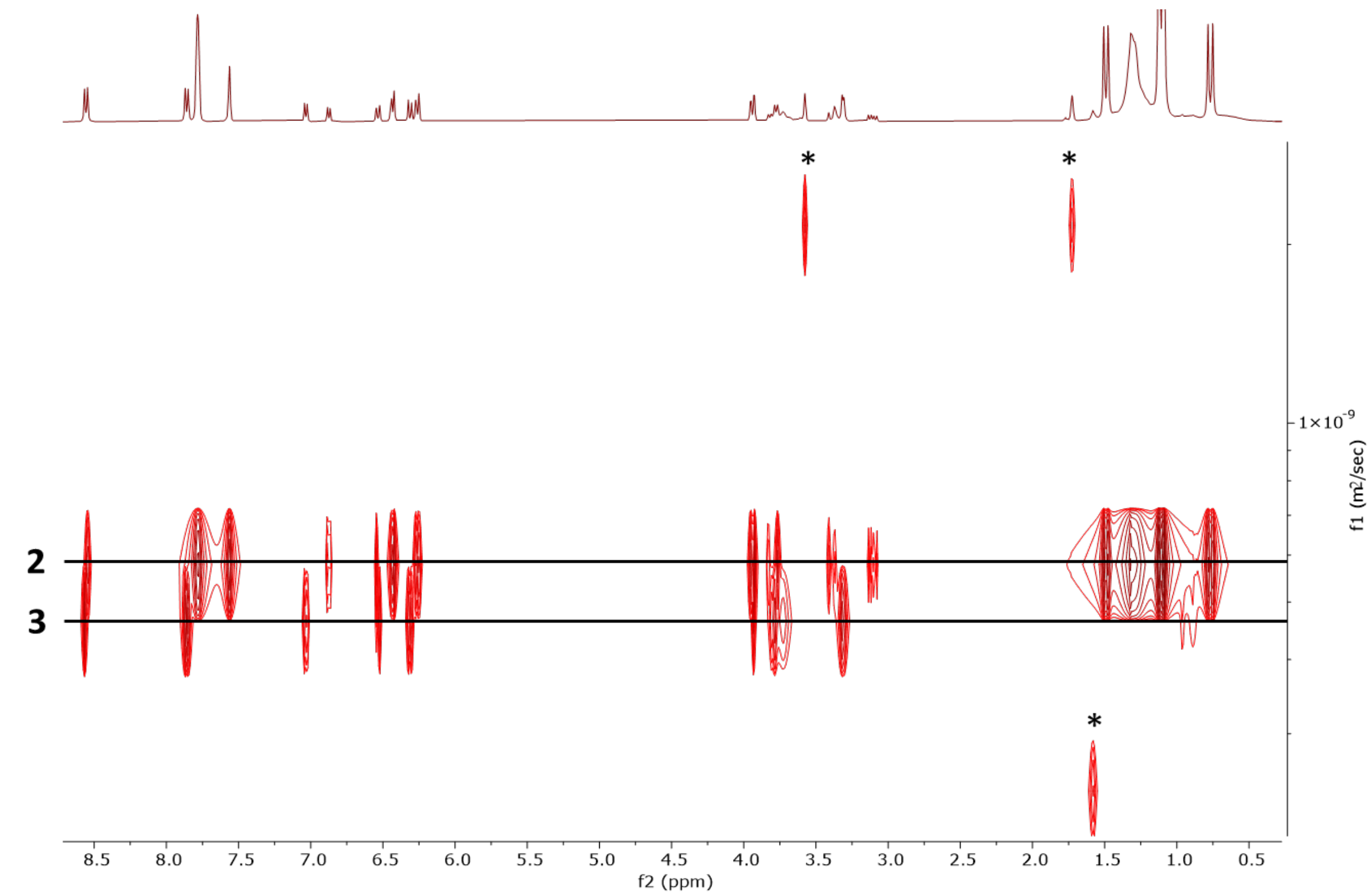

Figure S18. The ^{1}H DOSY NMR spectrum (THF-d_8, 298 K) of a mixture of **2** and **3**. *denotes THF-d_7 and traces of poly-THF.

1.7 X-ray crystal structure determination of 3

$[C_{52}H_{89}Cu_4N_4P_4](C_{32}H_{12}BF_{24})$, Fw = 2011.53, red plate, 0.43 × 0.32 × 0.07 mm^3, monoclinic, $P2_1/n$ (no. 14), a = 21.1039(3), b = 20.0450(3), c = 21.7343(4) Å, β = 91.370(1) °, V = 9191.6(3) $Å^3$, Z = 4, D_x = 1.454 g/cm^3, μ = 1.08 mm^{-1}. The diffraction experiment was performed on a Bruker Kappa ApexII diffractometer with sealed tube and Triumph monochromator (λ = 0.71073 Å) at a temperature of 150(2) K up to a resolution of $(\sin θ/λ)_{max}$ = 0.65 $Å^{-1}$. The Eval15 software[4] was used for the intensity integration. A multi-scan absorption correction and scaling was performed with SADABS[5] (correction range 0.67–0.75). A total of 186368 reflections was measured, 21122 reflections were unique (R_{int} = 0.039), 17317 reflections were observed [I>2σ(I)]. The structure was solved with Patterson superposition methods using SHELXT.[6] Structure refinement was performed with SHELXL-2018[7] on F^2 of all reflections. Non-hydrogen atoms were refined freely with anisotropic displacement parameters. Four of the CF_3 groups were refined with disorder models. The disorder could not be fully resolved. The metal-coordinated hydride atoms were located in difference Fourier maps and refined freely with isotropic displacement parameters. All other hydrogen atoms were introduced in calculated positions and refined with a riding model. 1234 Parameters were refined with 2700 restraints (distances and angles of the CF_3 groups, and displacement parameters of the whole BARF anion). R1/wR2 [I >2σ(I)]: 0.0345 / 0.0875. R1/wR2 [all refl.]: 0.0454 / 0.0924. S = 1.031. Residual electron density between -0.58 and 0.91 e/$Å^3$. Geometry calculations and checking for higher symmetry was performed with the PLATON program.[8]

1.8 X-ray crystal structure determination of 4

$[C_{52}H_{90}Cu_4N_4P_4](C_{32}H_{12}BF_{24})_2$, Fw = 2875.77, red plate, 0.34 × 0.20 × 0.06 mm^3, triclinic, $P\bar{1}$ (no. 2), a = 15.8777(6), b = 20.8543(9), c = 22.3701(8) Å, α = 93.061(1), β = 110.063(1), γ = 109.979(1) °, V = 6415.6(4) $Å^3$, Z = 2, D_x = 1.489 g/cm^3, μ = 0.82 mm^{-1}. The diffraction experiment was performed on a Bruker Kappa ApexII diffractometer with sealed tube and Triumph monochromator (λ = 0.71073 Å) at a temperature of 150(2) K up to a resolution of $(\sin θ/λ)_{max}$= 0.60 $Å^{-1}$. The Eval15 software[4] was used for the intensity integration. A multi-scan absorption correction and scaling was performed with SADABS[5] (correction range 0.62–0.75). A total of 78224 reflections was measured, 23167 reflections were unique (R_{int} = 0.121), 11342 reflections were observed [I>2σ(I)]. The structure was solved with Patterson superposition methods using SHELXT.[6] Structure refinement was performed with SHELXL-2018[7] on F^2 of all reflections. Non-hydrogen atoms were refined freely with anisotropic displacement parameters. Five of the CF_3 groups were refined with disorder models. The disorder could not be fully resolved. The metal-coordinated hydride atoms were located in difference Fourier maps and kept fixed. All other hydrogen atoms were introduced in calculated positions and refined with a riding model. 1762 Parameters were refined with 8529 restraints (distances and angles of the CF_3 groups, and displacement parameters of all atoms except Cu). R1/wR2 [I > 2σ(I)]: 0.0710 / 0.1521. R1/wR2 [all refl.]: 0.1697 / 0.1889. S = 1.023. Residual electron density between -0.47 and 0.80 e/$Å^3$. Geometry calculations and checking for higher symmetry was performed with the PLATON program.[8]

CCDC 2337836 (compound **3**) and 2337837 (compound **4**) contain the supplementary crystallographic data for this paper. These data can be obtained free of charge from The Cambridge Crystallographic Data Centre via www.ccdc.cam.ac.uk/data_request/cif.

2. Computational Details

2.1 Computational methods

All calculations were conducted using DFT[9] implemented in the ORCA 4.2.0 program package[10] with Becke's three-parameter exchange functional B3LYP including Grimme's D3 dispersion correction levels of theory.[11–17] All calculations were conducted using the def2/J auxiliary basis set and RIJCOSX approximation.[18,19] Geometry optimizations were proceeded using the def2-SVP basis set for all atoms except Cu atoms and def2-TZVP for Cu atoms.[20] The energies of the optimized structures were reevaluated by additional single-point calculations on each optimized geometry using the same functional and def2-TZVP basis set. Analytical vibrational frequencies within the harmonic approximation were calculated using the def2-SVP/def2-TZVP(Cu) mixed basis to confirm the proper convergence to well-defined minima or saddle points on the potential energy surface. Solvation energies were calculated using the CPCM model[21] and were performed with the def2-SVP/def2-TZVP(Cu) mixed basis at the optimized gas phase geometry with tetrahydrofuran (THF) as the solvent. The Gibbs free energies in solution phase G(sol) were computed with the following protocol.

$$G(sol) = G(gas) + G^{solv} \quad (1)$$

$$G(gas) = H(gas) – TS(gas) \quad (2)$$

$$H(gas) = E(SCF) + ZPE \quad (3)$$

$$\Delta E(SCF) = \Sigma E(SCF) \text{ for products} – \Sigma E(SCF) \text{ for reactants} \quad (4)$$

$$\Delta G(sol) = \Sigma G(sol) \text{ for products} – \Sigma G(sol) \text{ for reactants} \quad (5)$$

G(gas) is the free energy in gas phase; G^{solv} is the free energy of solvation; H(gas) is the enthalpy in gas phase; T is the temperature (298.15K); S(gas) is the entropy in gas phase; E(SCF) is "raw" electronic energy computed from the SCF (self-consistent field) procedure; ZPE is the zero-point energy. The entropy we refer to is specifically the vibrational/rotational/translational entropy of the solute(s), and the entropy of the solvent is implicitly comprised in the continuum solvation model.

Distortion-Interaction and Energy Decomposition analyses[22,23] were conducted as implemented in Amsterdam Density Functional (ADF) 2019 suite of *ab initio* quantum chemistry programs.[24] Calculations were conducted using B3LYP functional including Grimme's D3 dispersion correction and TZ2P basis set with no frozen core in the gas phase on structures that were optimized in ORCA. NMR spin-spin coupling constants were calculated using B3LYP-D3 functional with IGLO-III/def2-TZVP(Cu) mixed basis set and CPCM(THF) implicit solvation model.[25] Structures were visualized by Chemcraft[26] and diagrams were prepared using Matplotlib code[27].

In the Energy Decomposition Analysis, the interaction energy is quantitatively divided into the electrostatic interaction, Pauli repulsion, and orbital interactions and dispersion corrected interaction:

$$\Delta E_{int} = \Delta V_{elstat} + \Delta E_{Pauli} + \Delta E_{oi} + \Delta E_{disp}$$

2.2 Calculation of ^{2}J Coupling Constants

The calculated coupling constants for **1-mono** (4.0 Hz) match the experimentally observed value well. For complex **2-dimer**, the calculated ^{2}J coupling constants between the bridging deuteride and the phosphines on the aromatic side of the **PNNP*** ligand are small (0.2 and 0.7 Hz); however, the calculated coupling constant with the phosphine on the dearomatized side is larger (5.6 Hz). Since the latter coupling is experimentally not observed[2], we hypothesize that this is either an overestimation of the calculation or that a fluxional process decreases this coupling in solution since these constants are very sensitive to the geometry. For complex **4**, we calculated the coupling constants for both the monomeric (**4-mono**) and the dimeric structure (**4-dimer**). For **4-mono**, these coupling constants (6.3 and 5.1 Hz) are in line with what is expected for such a structure based on the observations for **1**. For **4-dimer**, the ^{2}J coupling constants (–0.4, 0.7, and 7.1 Hz) are in line with what was calculated for **2**. These calculations support that the ^{2}H-^{31}P coupling is sensitive to the aggregation state; however, the calculations consistently overestimate one of the coupling constants in the dimers.

2.3 Molecular Electrostatic Potential (MEP) Analysis

To further examine the influence of solvation on aggregation, we performed MEP calculations for both monomeric and dimeric species. The MEP surfaces of **4-mono** and **4-dimer** exhibit an overall blue shift (i.e., more positive potential) upon dimerization, indicating increased exposure of positive regions for interaction with the polar THF solvent. This trend is consistent with the stronger solvation stabilization observed for the dimerization of **4**. In contrast, the MEP surfaces of **1-mono** and **2-mono** show either a red shift or minimal change upon dimerization, reflecting their relatively weaker solvation contributions. These results qualitatively corroborate our proposed interpretation of solvation effects in governing the aggregation behavior.

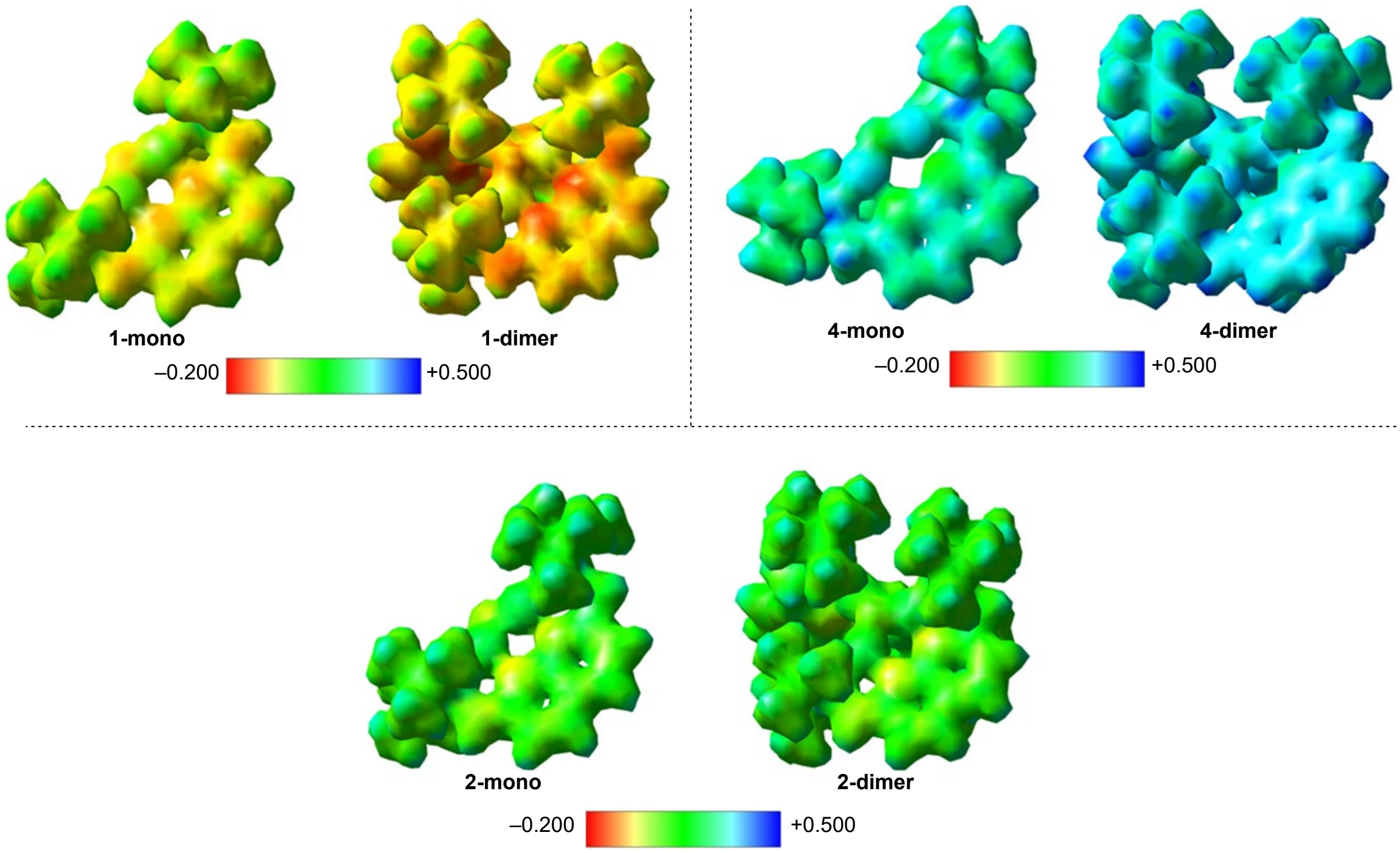

Figure S19. Molecular electrostatic potential (MEP) surfaces of **1-mono**, **1-dimer**, **2-mono**, **2-dimer**, **4-mono**, and **4-dimer**. Blue regions indicate more positive electrostatic potential, while red regions indicate more negative potential.

2.4 Hirshfeld Analysis

To support the interpretation that protonation induces a redistribution of electron density, we performed Hirshfeld charge analyses. The results indicate an increase in partial charge at the bridging hydride, both copper atoms, and across the conjugated π-system, including the binaphthyl and methylene moieties, upon protonation of **1-mono** to **2-mono**. This effect becomes more pronounced upon double protonation to form **4-mono**, consistent with a progressive redistribution of electron density across the series of monomers.

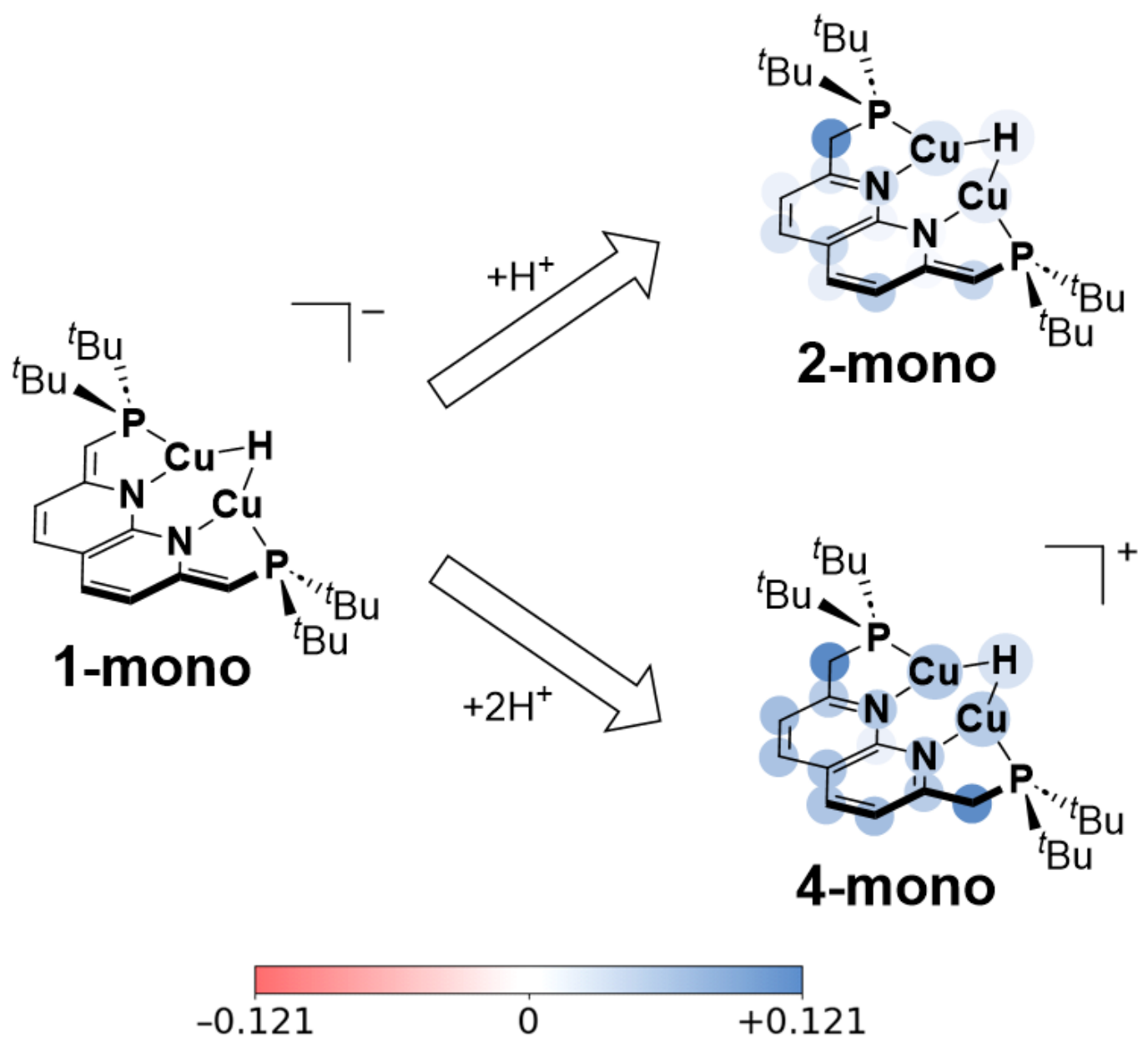

Figure S20. Changes in Hirshfeld charges of **2-mono** and **4-mono** relative to **1-mono**. The color scale represents the magnitude of charge variation, with red indicating a decrease and blue indicating an increase.

2.5 DFT-energy components of all optimized geometries

Supplementary Table S1. Computed energy components for optimized structures.

	**E(SCF) /(eV)**	**ZPE /(kcal/mol)**	**S(gas) /(cal/mol·K)**	**Gsolv /(kcal/mol)**
	**B3LYP-D3 /def2-TZVP**	**B3LYP-D3 /def2-SVP, def2-TZVP(Cu)**	**B3LYP-D3 /def2-SVP, def2-TZVP(Cu)**	**B3LYP-D3 /def2-SVP, def2-TZVP(Cu)**
**1-mono**	-138496.257	396.38	215.60	-34.95
**1-dimer**	-276992.277	794.37	369.11	-93.21
**2-mono**	-138511.470	405.85	215.76	-1.97
**2-dimer**	-277024.946	813.81	371.22	-0.81
**4-mono**	-138523.460	414.69	216.40	-26.42
**4-dimer**	-277047.130	831.22	373.70	-86.19

2.6 Cartesian coordinates for DFT-optimized structures

Supplementary Table S2. Cartesian coordinates of the optimized geometries.

==============================

1-mono

==============================

C	0.483950	-4.635340	-4.015619
C	-0.128749	-3.478064	-3.216566
C	-1.361982	-2.892630	-3.929707
C	-0.581904	-3.995502	-1.834302
P	1.107812	-2.068417	-2.791782
C	2.053951	-1.453876	-4.340380
C	1.101148	-1.093929	-5.489919
C	2.738320	-0.162039	-3.842468
C	3.136292	-2.424767	-4.841231
C	2.331950	-2.740395	-1.703088
C	2.344909	-2.323267	-0.356822
C	3.274159	-2.907695	0.585680
C	3.258221	-2.511028	1.898200
C	2.347120	-1.519797	2.349201
C	2.244732	-1.053155	3.685644
C	1.341093	-0.078759	4.024266
C	1.452569	-0.948163	1.377317
N	1.467816	-1.358038	0.084853
Cu	0.127470	-0.579836	-1.383260
H	-1.076751	0.528305	-1.258803
Cu	-0.822341	0.839760	0.332494
N	0.563528	0.015631	1.724173
C	0.480515	0.493379	3.012204
C	-0.422897	1.525328	3.334789
P	-1.455609	2.179898	2.054130
C	-0.932933	4.014066	1.819975
C	-1.907891	4.759910	0.900430
C	-0.737269	4.778598	3.137338
C	0.432764	3.896592	1.109541
C	-3.272233	2.036954	2.651244
C	-4.230410	2.267554	1.469126
C	-3.412907	0.566440	3.094294
C	-3.631875	2.961006	3.822941
H	-0.231432	-5.476697	-4.087519
H	1.396896	-5.014344	-3.526892
H	0.742784	-4.336783	-5.044349
H	-2.156643	-3.658536	-4.003866
H	-1.142180	-2.546169	-4.950199
H	-1.760163	-2.035705	-3.362016
H	-1.370127	-4.760233	-1.960550
H	-0.993098	-3.177640	-1.218594
H	0.254933	-4.436586	-1.273618
H	0.276986	-0.449673	-5.143547
H	0.666568	-1.987455	-5.964383
H	1.654198	-0.543001	-6.273565
H	1.991383	0.578049	-3.513797
H	3.341956	0.286126	-4.653261
H	3.399508	-0.372693	-2.987934
H	2.710646	-3.355896	-5.245225
H	3.837019	-2.688688	-4.034906
H	3.719049	-1.947474	-5.651649
H	3.022942	-3.532240	-2.005861
H	3.973228	-3.668877	0.230568
H	3.950555	-2.958389	2.621733
H	2.905943	-1.487375	4.445820
H	1.258191	0.289697	5.050079
H	-0.463484	1.880443	4.368882
H	-2.859329	4.989870	1.406260
H	-2.127775	4.173419	-0.006688
H	-1.465668	5.722171	0.579885
H	-1.686246	4.958911	3.663752
H	-0.274936	5.764127	2.937096
H	-0.067612	4.224453	3.812858
H	0.335742	3.387489	0.136108
H	1.143121	3.315759	1.717781
H	0.857864	4.902577	0.934751
H	-3.949324	1.633959	0.612773
H	-4.236642	3.310537	1.120687
H	-5.263719	2.011204	1.770016
H	-3.144836	-0.120128	2.274115
H	-4.459196	0.362800	3.388984
H	-2.751408	0.336111	3.941672
H	-3.647422	4.019375	3.517761
H	-2.919300	2.852981	4.656552
H	-4.638346	2.714358	4.211632

==============================

1-dimer

==============================

Cu	5.794115	9.061970	5.111135
Cu	7.541277	8.365731	6.822735
H	7.689910	6.601670	6.337624
H	7.417370	8.819814	5.104849
P	4.110265	9.782938	3.693881
P	9.280812	8.570741	8.368560
N	4.804028	10.111527	6.630462
N	6.413500	9.527925	8.210885
C	3.254459	10.781994	4.887746
H	2.375348	11.377913	4.625618
C	3.680499	10.805800	6.225785
C	2.941689	11.575406	7.204910
H	2.047699	12.113951	6.880092
C	3.368355	11.615720	8.505077
H	2.813958	12.190660	9.257774
C	4.542967	10.929587	8.906591
C	5.260435	10.179318	7.906059
C	5.073796	10.957254	10.220715
H	4.523685	11.498786	11.000724
C	6.265854	10.348522	10.503894
H	6.707604	10.401019	11.502574
C	6.999185	9.672409	9.454270
C	8.299395	9.207650	9.704519
H	8.703846	9.361471	10.710464
C	4.891947	10.982307	2.400805

C	5.774250	10.184347	1.421987
H	6.464993	10.865935	0.890047
H	5.185590	9.657506	0.659538
H	6.378834	9.426357	1.945311
C	3.881728	11.850035	1.639136
H	3.223694	12.399831	2.332990
H	3.250475	11.252029	0.963273
H	4.413696	12.595646	1.015945
C	5.800974	11.903171	3.241358
H	6.344278	12.599639	2.575064
H	6.547399	11.320577	3.805147
H	5.215795	12.485569	3.969590
C	2.729691	8.735315	2.863997
C	3.201893	8.104107	1.548322
H	4.191635	7.638302	1.642111
H	3.237670	8.841418	0.730738
H	2.498245	7.308672	1.243733
C	1.422294	9.512025	2.617461
H	1.550106	10.342231	1.906402
H	1.013540	9.921816	3.554102
H	0.664833	8.826898	2.190682
C	2.451138	7.622957	3.892874
H	1.661728	6.946328	3.518046
H	2.114943	8.053681	4.847747
H	3.344081	7.016019	4.095695
C	10.737329	9.835411	8.221772
C	11.859613	9.295571	7.323319
H	11.459265	8.906344	6.373357
H	12.425541	8.487247	7.811842
H	12.579560	10.099958	7.080102
C	11.316367	10.296004	9.570969
H	12.055024	11.105441	9.409890
H	11.830041	9.488078	10.110542
H	10.522997	10.692975	10.222728
C	10.075962	11.050351	7.539316
H	9.235804	11.426424	8.143829
H	9.673820	10.784589	6.549058
H	10.812874	11.866673	7.417127
C	9.980134	6.873059	8.946090
C	10.554759	6.124103	7.728800
H	11.456507	6.605184	7.322407
H	9.804537	6.067848	6.923956
H	10.834637	5.092614	8.016514
C	11.022213	6.945337	10.070934
H	10.646464	7.521005	10.932869
H	11.965922	7.402079	9.734159
H	11.263286	5.925664	10.429829
C	8.752214	6.097751	9.456673
H	9.043888	5.064554	9.724479
H	7.968440	6.049519	8.683736
H	8.316556	6.583120	10.342488
Cu	6.211844	5.927239	6.424626
Cu	7.570823	7.135604	4.644405
P	4.651924	5.019454	7.848849
P	9.160038	7.157375	2.943654
N	5.049931	5.106088	4.864776
N	6.370400	6.105465	3.233804
C	3.731805	4.103345	6.633633
H	2.869808	3.482215	6.895499
C	4.012867	4.289316	5.270286
C	3.211042	3.624929	4.263765
H	2.396328	2.974117	4.591584
C	3.473109	3.829146	2.934863
H	2.861201	3.340440	2.166088
C	4.534073	4.676767	2.521788
C	5.328086	5.302605	3.550542
C	4.867395	4.955762	1.172173
H	4.252118	4.513667	0.378152
C	5.934732	5.759386	0.864021
H	6.209845	5.976374	-0.171557
C	6.760110	6.294464	1.925270
C	7.969492	6.946574	1.633346
H	8.262174	7.013196	0.580933
C	5.190982	3.713935	9.157470
C	5.640288	4.366707	10.475350
H	6.168609	3.621186	11.099678
H	4.785448	4.737759	11.060483
H	6.325023	5.209739	10.305830
C	4.117290	2.652290	9.452373
H	4.525714	1.885925	10.139723
H	3.793774	2.141457	8.532566
H	3.226662	3.085338	9.933354
C	6.403142	3.025219	8.496880
H	7.218778	3.743086	8.324199
H	6.122286	2.595295	7.521982
H	6.783913	2.210965	9.142827
C	3.442218	6.299861	8.601305
C	4.172192	7.227711	9.585171
H	3.546186	8.110540	9.804056
H	5.109561	7.604483	9.150324
H	4.402323	6.740732	10.542851
C	2.203109	5.665439	9.250275
H	2.455573	5.111686	10.169350
H	1.695679	4.975321	8.555006
H	1.479174	6.453517	9.531564
C	3.010207	7.140686	7.382551
H	2.357241	7.968905	7.709047
H	2.469009	6.528742	6.647925
H	3.874159	7.597069	6.872786
C	10.335929	5.614865	2.744758
C	11.373904	5.565102	3.878126
H	10.898082	5.756156	4.853240
H	12.187495	6.290443	3.744998
H	11.839231	4.561616	3.922018
C	11.015912	5.507337	1.373017
H	11.554526	4.543158	1.281851
H	11.754148	6.307423	1.203258
H	10.269607	5.547544	0.562725
C	9.391465	4.406579	2.920087
H	9.983047	3.471758	2.890665
H	8.635635	4.361594	2.122998
H	8.852508	4.455430	3.880127
C	10.132581	8.760158	2.488851
C	11.553524	8.758977	3.073175
H	11.998163	9.765963	2.965647
H	12.218377	8.052395	2.554345
H	11.553547	8.511724	4.145716
C	10.211792	9.003729	0.971914
H	9.209833	9.147184	0.541079
H	10.697350	8.170711	0.440488

H	10.804336	9.916931	0.768683
C	9.354130	9.921573	3.137284
H	9.825796	10.884277	2.862933
H	9.330234	9.832899	4.232673
H	8.308315	9.942411	2.804593

==============================

2-mono

==============================

C	0.067830	-6.763328	-1.400075
C	-0.717208	-5.442737	-1.463013
C	-1.866612	-5.539069	-2.478570
C	-1.301370	-5.155642	-0.063015
P	0.362026	-3.912238	-1.831060
C	0.991614	-3.878263	-3.628697
C	-0.148437	-3.372887	-4.536563
C	2.105365	-2.811084	-3.627480
C	1.548168	-5.214421	-4.140010
C	1.785761	-4.061333	-0.749424
C	1.949116	-3.138176	0.274636
C	3.061140	-3.257753	1.213101
C	3.185599	-2.408262	2.264554
C	2.213808	-1.363461	2.468181
C	2.213810	-0.480433	3.548783
C	1.207320	0.483188	3.671643
C	1.164040	-1.253347	1.484442
N	1.055673	-2.092928	0.446152
Cu	-0.460324	-1.945711	-1.060341
H	-0.468184	2.546611	2.314758
Cu	-1.416919	-0.100402	0.145958
N	0.218536	-0.269010	1.610665
C	0.228507	0.554011	2.669482
C	-0.862471	1.612690	2.750268
P	-2.369429	1.188705	1.729806
C	-3.121902	2.890441	1.330837
C	-4.528984	2.687061	0.738880
C	-3.189458	3.862306	2.521915
C	-2.210736	3.481462	0.231925
C	-3.473454	0.123210	2.859886
C	-4.559969	-0.525496	1.976469
C	-2.565961	-1.005200	3.395458
C	-4.111560	0.870954	4.037597
H	-0.601164	-7.574168	-1.059115
H	0.899817	-6.697133	-0.682702
H	0.480032	-7.060865	-2.375037
H	-2.591465	-6.305096	-2.152205
H	-1.511144	-5.832573	-3.478081
H	-2.405759	-4.582410	-2.570976
H	-1.988524	-5.968621	0.227847
H	-1.863883	-4.207582	-0.047507
H	-0.505356	-5.089767	0.694520
H	-0.589397	-2.447838	-4.130724
H	-0.958090	-4.106362	-4.654635
H	0.247397	-3.151325	-5.543758
H	1.731033	-1.850741	-3.233666
H	2.458219	-2.640580	-4.659705
H	2.964055	-3.113268	-3.010673
H	0.759217	-5.974288	-4.243446
H	2.327933	-5.616028	-3.473316
H	2.003738	-5.079697	-5.137885
H	2.518684	-4.866864	-0.848167
H	3.783644	-4.062342	1.053964
H	4.012281	-2.511580	2.974497
H	3.002411	-0.557574	4.304414
H	1.181525	1.169682	4.519754
H	-1.108079	1.837111	3.800386
H	-5.256683	2.374971	1.502345
H	-4.527305	1.938674	-0.069375
H	-4.888948	3.639071	0.311958
H	-3.829000	3.488225	3.332350
H	-3.615614	4.823914	2.184969
H	-2.195730	4.078214	2.943953
H	-1.193049	3.691521	0.599136
H	-2.634268	4.436719	-0.123745
H	-2.124761	2.799693	-0.628926
H	-4.107605	-1.061512	1.126747
H	-5.272087	0.205864	1.571843
H	-5.134793	-1.251919	2.576709
H	-2.054679	-1.536554	2.578011
H	-3.185382	-1.735160	3.943454
H	-1.796173	-0.636097	4.089879
H	-4.848554	1.616925	3.704641
H	-3.358886	1.379885	4.660842
H	-4.643621	0.153390	4.686477
H	-1.614057	-0.814986	-1.304145

==============================

2-dimer

==============================

Cu	5.979218	9.194798	4.956344
Cu	7.574210	8.195592	6.715961
H	7.539449	6.488720	6.260316
H	7.537001	8.750006	5.053260
P	4.333790	10.009958	3.590913
P	9.313504	8.325674	8.206273
N	4.975666	10.097126	6.563348
N	6.436672	9.241372	8.162841
C	3.481467	10.954259	4.858199
H	2.617732	11.582849	4.626227
C	3.892964	10.877105	6.180648
C	3.181424	11.629823	7.207348
H	2.334322	12.245551	6.896791
C	3.559537	11.567403	8.507875
H	3.026828	12.132956	9.278204
C	4.680792	10.750240	8.895778
C	5.358227	10.025965	7.849779
C	5.150580	10.641249	10.203897
H	4.639224	11.186399	11.003414
C	6.274840	9.860904	10.482031
H	6.669730	9.774716	11.495417
C	6.899910	9.189462	9.423615
C	8.148798	8.367056	9.678482
H	7.848138	7.314867	9.813601
H	8.635385	8.688575	10.611477
C	4.896185	11.311744	2.306317
C	5.522286	10.628629	1.078129
H	6.083094	11.370798	0.483002
H	4.762308	10.186378	0.418974
H	6.223923	9.831954	1.371211
C	3.792966	12.282743	1.856946
H	3.319434	12.787923	2.712414
H	3.006917	11.781498	1.274811

H	4.230066	13.065622	1.211128
C	5.990584	12.105909	3.049429
H	6.409418	12.882338	2.385156
H	6.813076	11.447465	3.370601
H	5.583009	12.597645	3.946030
C	3.034839	8.794068	2.900259
C	3.651197	7.922034	1.794948
H	4.629652	7.526565	2.098297
H	3.772376	8.465069	0.847918
H	2.996625	7.056778	1.593980
C	1.746193	9.461883	2.397387
H	1.912427	10.054021	1.485278
H	1.300371	10.116326	3.162960
H	1.002244	8.683997	2.148679
C	2.707156	7.898191	4.112073
H	2.006204	7.101325	3.809766
H	2.240642	8.478999	4.919871
H	3.607820	7.412443	4.519773
C	10.280421	9.973565	8.294833
C	11.122730	10.101358	7.008796
H	10.502416	9.919995	6.116619
H	11.977570	9.414007	6.978603
H	11.527140	11.125921	6.936196
C	11.156982	10.156753	9.541292
H	11.584783	11.175143	9.551671
H	12.000793	9.452096	9.569633
H	10.575514	10.040346	10.469881
C	9.215498	11.091613	8.251598
H	8.629049	11.152601	9.180413
H	8.513375	10.946177	7.415704
H	9.723855	12.061366	8.111680
C	10.445554	6.836316	8.628568
C	11.741761	6.917738	7.800911
H	12.415794	7.709726	8.156474
H	11.536023	7.084804	6.731603
H	12.285844	5.961631	7.885714
C	10.794865	6.712731	10.124287
H	9.906531	6.502957	10.738855
H	11.283744	7.611336	10.524770
H	11.493137	5.869623	10.269265
C	9.682789	5.569604	8.185065
H	10.293506	4.679942	8.417687
H	9.466241	5.584376	7.108556
H	8.716943	5.451808	8.695319
Cu	5.960476	6.114242	6.445808
Cu	7.474034	7.039663	4.578990
P	4.408667	5.291473	7.905670
P	9.122985	6.970029	2.985372
N	4.908745	5.114000	4.916213
N	6.293397	5.918761	3.224395
C	3.514861	4.287769	6.718463
H	2.691303	3.635274	7.018987
C	3.865141	4.323159	5.376858
C	3.133044	3.506348	4.415930
H	2.309053	2.892923	4.787399
C	3.469976	3.503686	3.102779
H	2.926474	2.886669	2.381001
C	4.562285	4.318772	2.636457
C	5.248648	5.122799	3.616917
C	4.995603	4.353988	1.312279

H	4.483244	3.741555	0.563863
C	6.082443	5.153105	0.951550
H	6.446149	5.182913	-0.076625
C	6.706092	5.919370	1.943771
C	7.881993	6.807963	1.587694
H	7.505893	7.834670	1.440278
H	8.334127	6.488806	0.636594
C	5.184469	4.059540	9.146554
C	6.108441	4.819886	10.115033
H	6.717082	4.104011	10.695665
H	5.556640	5.442375	10.831733
H	6.792566	5.474517	9.553864
C	4.170558	3.213922	9.928091
H	4.702742	2.455370	10.530140
H	3.479690	2.682260	9.254560
H	3.574525	3.823800	10.623586
C	6.062471	3.135282	8.274544
H	6.786512	3.716932	7.678294
H	5.455370	2.539792	7.576219
H	6.630754	2.444942	8.922919
C	3.078407	6.402002	8.708287
C	3.649965	7.147439	9.923419
H	2.963534	7.959388	10.219120
H	4.623719	7.608563	9.701277
H	3.763656	6.485237	10.794843
C	1.787451	5.662923	9.102313
H	1.941938	4.931747	9.908535
H	1.341681	5.140855	8.241795
H	1.046227	6.397215	9.465007
C	2.747071	7.414448	7.595054
H	2.015713	8.155512	7.961421
H	2.311431	6.908387	6.721420
H	3.640032	7.958465	7.255070
C	10.206670	5.398946	2.858349
C	11.119773	5.354722	4.100567
H	10.534734	5.500774	5.022182
H	11.915533	6.110519	4.074155
H	11.609009	4.367418	4.163300
C	11.037107	5.259720	1.573942
H	11.532270	4.272515	1.556197
H	11.829178	6.019766	1.500504
H	10.410329	5.323916	0.670335
C	9.229684	4.206987	2.960045
H	9.812489	3.276838	3.075058
H	8.604307	4.098967	2.061615
H	8.560875	4.305929	3.829655
C	10.101930	8.540669	2.485053
C	11.396261	8.612670	3.315829
H	11.850202	9.612697	3.205660
H	12.144256	7.876932	2.988374
H	11.198433	8.456917	4.388281
C	10.435376	8.630452	0.984117
H	9.528666	8.732066	0.368371
H	11.003792	7.766023	0.617343
H	11.051851	9.528309	0.801074
C	9.226750	9.752234	2.867017
H	9.741054	10.679101	2.558496
H	9.027832	9.790255	3.945647
H	8.247491	9.738756	2.368982

==============================

4-mono

H	6.927240	7.623648	5.844581
Cu	5.760085	6.579743	6.229741
Cu	7.366497	7.057459	4.396778
P	4.305665	5.465059	7.555936
P	9.075362	6.721257	2.927713
N	4.549065	5.373375	4.552069
N	6.237468	5.672027	2.990746
C	2.927881	5.096423	6.349665
H	2.315981	6.013577	6.317141
C	3.417865	4.807461	4.949961
C	2.664643	3.943524	4.105441
H	1.734025	3.507067	4.473032
C	3.131393	3.662745	2.842482
H	2.581115	2.997608	2.171475
C	4.357355	4.233349	2.414365
C	5.041138	5.105287	3.315975
C	4.962666	3.966695	1.159370
H	4.460768	3.309707	0.444136
C	6.183357	4.529612	0.871425
H	6.682389	4.330427	-0.078883
C	6.803562	5.389201	1.823540
C	8.133211	6.022036	1.472743
H	7.921976	6.879022	0.810947
H	8.739104	5.324205	0.874608
C	4.967421	3.759302	8.082421
C	6.294365	3.979646	8.839360
H	6.742361	3.001096	9.081363
H	6.164119	4.522841	9.784305
H	7.014166	4.539293	8.220634
C	3.983092	2.945157	8.933046
H	4.404185	1.943134	9.123137
H	3.015368	2.801899	8.425281
H	3.796127	3.409341	9.911673
C	5.290539	2.983482	6.786681
H	5.918848	3.575139	6.102816
H	4.386318	2.670931	6.242345
H	5.845643	2.067275	7.047489
C	3.397301	6.389744	8.946018
C	4.289807	6.427195	10.200721
H	3.842348	7.110408	10.941179
H	5.302097	6.799052	9.974742
H	4.377555	5.442142	10.680888
C	2.016921	5.802560	9.299383
H	2.073041	4.769023	9.667434
H	1.316692	5.828930	8.451121
H	1.566259	6.409270	10.102442
C	3.223198	7.831237	8.417519
H	2.725778	8.445347	9.186168
H	2.597654	7.878905	7.511169
H	4.193982	8.295428	8.182478
C	10.128662	5.279208	3.592162
C	10.700936	5.713337	4.959448
H	9.904206	6.071974	5.631003
H	11.453562	6.508010	4.871916
H	11.191526	4.849330	5.438686
C	11.258074	4.817240	2.660793
H	11.755739	3.938268	3.104505
H	12.026765	5.589833	2.519498
H	10.885665	4.514428	1.668914
C	9.150329	4.108609	3.830911
H	9.688390	3.286530	4.331386
H	8.736427	3.704931	2.893392
H	8.310091	4.403085	4.480656
C	10.105299	8.096376	2.108620
C	11.238303	8.537064	3.052675
H	11.719154	9.437043	2.634727
H	12.019170	7.769957	3.155483
H	10.864244	8.794628	4.056353
C	10.696681	7.700562	0.741106
H	9.923342	7.453618	-0.001548
H	11.389551	6.851120	0.808097
H	11.264915	8.555107	0.336968
C	9.127971	9.278222	1.926598
H	9.656687	10.118798	1.446810
H	8.731068	9.630069	2.891788
H	8.271767	9.026557	1.279750
H	2.267205	4.291081	6.702780

4-dimer

H	-0.431706	0.864871	-1.083847
Cu	-0.723942	1.156702	0.482715
Cu	1.262100	0.378489	-1.072830
P	-2.152152	2.022948	2.043507
P	2.668990	0.846464	-2.831338
N	0.884415	2.246640	1.682001
N	2.511486	1.886948	0.044029
C	-0.895325	2.490875	3.343304
H	-0.736558	1.578315	3.934334
C	0.438491	2.891869	2.761055
C	1.182863	3.924051	3.391856
H	0.775594	4.423364	4.272440
C	2.401392	4.288383	2.868887
H	2.993486	5.089988	3.318562
C	2.890027	3.618568	1.720444
C	2.097762	2.570677	1.152405
C	4.112178	3.959007	1.087821
H	4.735390	4.755007	1.503447
C	4.475163	3.291940	-0.055347
H	5.395408	3.546049	-0.584116
C	3.642960	2.252651	-0.559646
C	4.076792	1.544397	-1.820331
H	4.683914	0.672391	-1.524354
H	4.739850	2.194616	-2.409851
C	-2.832506	3.707840	1.440058
C	-4.194589	3.503053	0.752918
H	-4.467301	4.422465	0.209175
H	-4.995606	3.305700	1.478654
H	-4.172984	2.682293	0.020546
C	-2.972899	4.758007	2.555508
H	-3.419766	5.672358	2.130718
H	-2.000862	5.048035	2.982922
H	-3.626852	4.426315	3.373292
C	-1.819888	4.220673	0.389820
H	-1.699340	3.514997	-0.448344
H	-0.825717	4.406174	0.825085
H	-2.182432	5.176046	-0.024622
C	-3.430320	1.001267	3.022800

C	-4.494690	0.460932	2.049207
H	-5.137706	-0.257349	2.583744
H	-4.020941	-0.069173	1.213381
H	-5.146874	1.241153	1.642800
C	-4.100124	1.781390	4.164660
H	-4.742605	2.590307	3.787943
H	-3.368798	2.214071	4.864742
H	-4.744232	1.101019	4.747311
C	-2.670662	-0.217968	3.586725
H	-3.393154	-0.917145	4.038015
H	-1.954215	0.049075	4.375761
H	-2.128753	-0.757008	2.794238
C	2.055823	2.284489	-3.913985
C	0.730392	1.819983	-4.548845
H	0.058781	1.402816	-3.783959
H	0.876036	1.059165	-5.326791
H	0.223270	2.677390	-5.021248
C	3.025489	2.773258	-4.997837
H	2.586784	3.647556	-5.507780
H	3.211608	2.009208	-5.765318
H	3.995071	3.090318	-4.582170
C	1.739174	3.439400	-2.940534
H	1.222955	4.243690	-3.489866
H	2.648422	3.878972	-2.501846
H	1.079077	3.109725	-2.121780
C	3.556759	-0.514592	-3.830455
C	2.651240	-0.989948	-4.981277
H	3.099494	-1.885690	-5.442149
H	2.551811	-0.234973	-5.774193
H	1.644711	-1.265665	-4.627255
C	4.925965	-0.083764	-4.392128
H	5.638379	0.198503	-3.602275
H	4.851892	0.751232	-5.100898
H	5.373513	-0.932998	-4.935512
C	3.754685	-1.688852	-2.847313
H	4.253180	-2.521992	-3.369919
H	2.796252	-2.049928	-2.446259
H	4.395349	-1.421348	-1.992631
H	-1.265214	3.255032	4.038405
H	0.804963	-1.377738	-1.235339
Cu	0.980698	-1.696907	0.340790
Cu	-0.889262	-0.843374	-1.246740
P	2.363184	-2.172306	2.095574
P	-2.683381	-1.003720	-2.676823
N	-0.419648	-2.998840	1.340559
N	-2.206887	-2.141003	0.123488
C	1.586796	-3.846145	2.421705
H	1.888809	-4.298681	3.374793
C	0.084122	-3.801457	2.278065
C	-0.742371	-4.622490	3.089743
H	-0.288391	-5.253774	3.855617
C	-2.103021	-4.622799	2.876157
H	-2.765836	-5.254245	3.473635
C	-2.649513	-3.800624	1.857392
C	-1.759892	-2.971319	1.107768
C	-4.027623	-3.764898	1.522723
H	-4.736369	-4.388827	2.073528
C	-4.443570	-2.958486	0.490334
H	-5.493069	-2.929625	0.192481
C	-3.496332	-2.151572	-0.203014

C	-3.965076	-1.267881	-1.333432
H	-4.158928	-0.262132	-0.925223
H	-4.921679	-1.633933	-1.733033
C	4.166976	-2.548694	1.651099
C	4.804056	-1.236881	1.137914
H	5.768666	-1.466137	0.656066
H	5.008390	-0.515844	1.939601
H	4.151617	-0.750972	0.394626
C	4.981256	-3.149301	2.806691
H	5.974540	-3.460026	2.441783
H	4.497339	-4.042235	3.233749
H	5.144686	-2.423552	3.616003
C	4.112124	-3.536451	0.465427
H	3.420115	-3.189736	-0.320255
H	3.818170	-4.551302	0.772934
H	5.114143	-3.618135	0.014905
C	2.168641	-1.254118	3.752950
C	3.112889	-0.037707	3.773840
H	2.831403	0.628103	4.606507
H	3.049627	0.546663	2.843450
H	4.159810	-0.334589	3.931537
C	2.392895	-2.119499	5.005113
H	3.395008	-2.567009	5.049812
H	1.651921	-2.928506	5.091610
H	2.270594	-1.486045	5.900287
C	0.713654	-0.752949	3.756269
H	0.547805	-0.144370	4.660917
H	-0.010948	-1.578208	3.782391
H	0.498167	-0.136733	2.869566
C	-2.807405	-2.566392	-3.758599
C	-1.632400	-2.527525	-4.756650
H	-0.675443	-2.366959	-4.232357
H	-1.745265	-1.746433	-5.519305
H	-1.570987	-3.492347	-5.286310
C	-4.145719	-2.743749	-4.493094
H	-4.143682	-3.707862	-5.028882
H	-4.326668	-1.959487	-5.241018
H	-5.001408	-2.764910	-3.799794
C	-2.585124	-3.769629	-2.816299
H	-2.492093	-4.683615	-3.425277
H	-3.429032	-3.929772	-2.127930
H	-1.663513	-3.659468	-2.222385
C	-3.364846	0.557106	-3.554361
C	-2.748158	0.683149	-4.960137
H	-2.969482	1.686294	-5.360491
H	-3.169833	-0.046471	-5.665232
H	-1.655578	0.566484	-4.950450
C	-4.900502	0.583477	-3.668478
H	-5.393648	0.666241	-2.688251
H	-5.304871	-0.294996	-4.187721
H	-5.200034	1.469923	-4.252000
C	-2.907047	1.759576	-2.702969
H	-3.349295	2.683500	-3.111149
H	-1.814826	1.865257	-2.694859
H	-3.226471	1.674251	-1.652899
H	1.967282	-4.503088	1.623948

2.7 Vibrational frequencies for DFT-optimized structures

Supplementary Table S3. Vibrational frequencies (in cm^{-1}) of the optimized geometries.

==============================

1-mono

==============================

14.35 20.99 31.43 39.35 45.78 50.72 52.92 57.42
71.51 83.76 100.18 104.33 115.53 116.90 128.13 129.15
140.15 164.50 182.89 193.32 220.73 225.33 231.86
233.99 235.92 238.62 242.41 250.43 252.83 257.70
260.74 262.26 264.51 272.57 278.31 288.76 299.96
304.31 307.98 312.56 319.29 321.24 325.03 327.54
331.55 333.28 364.75 379.01 386.38 390.73 397.43
402.93 413.29 414.81 432.82 436.58 437.30 454.14
457.92 467.47 475.00 476.68 501.32 507.56 527.63
528.40 585.41 588.32 604.33 605.32 617.38 620.66
648.46 685.75 710.34 724.74 792.01 795.76 803.68
813.61 817.66 819.13 819.87 821.81 832.37 874.52
903.89 940.53 941.45 942.35 943.58 944.96 949.36
949.62 950.53 952.03 961.44 961.77 962.25 962.63
963.36 979.79 1023.70 1025.06 1033.14 1034.14 1034.76
1035.52 1035.53 1037.37 1068.30 1132.19 1156.38 1159.56
1179.25 1197.15 1198.74 1200.50 1201.40 1210.71 1212.69
1223.00 1224.05 1227.74 1229.38 1231.01 1232.78 1256.43
1278.99 1295.94 1332.21 1350.61 1369.69 1374.81 1376.05
1377.98 1378.07 1378.65 1380.25 1381.07 1381.73 1403.78
1404.30 1404.93 1408.62 1409.83 1423.14 1433.34 1441.27
1442.58 1446.03 1447.24 1455.99 1456.55 1458.96 1459.25
1461.31 1461.58 1464.82 1465.15 1469.67 1469.94 1470.68
1471.87 1476.97 1478.31 1481.96 1482.12 1488.09 1491.00
1491.10 1497.28 1498.06 1511.93 1543.24 1638.16 1654.54
2989.40 2990.35 2991.25 2991.90 2994.14 2994.72 2995.09
2995.76 2998.73 3001.32 3001.82 3003.81 3065.73 3068.68
3069.82 3073.72 3075.52 3077.35 3079.81 3081.72 3082.26
3083.39 3085.56 3085.76 3100.75 3104.59 3107.93 3110.65
3112.12 3115.48 3121.64 3122.52 3123.54 3123.74 3124.09
3124.29 3125.31 3128.48 3146.05 3150.86 3170.19 3173.75

==============================

1-dimer

==============================

14.98 21.82 25.96 29.17 32.91 36.83 39.14 42.37
46.75 48.25 50.29 52.18 55.58 60.73 65.09 66.25 69.09
75.88 77.18 80.53 83.59 85.09 86.98 87.68 95.13 96.83
102.41 107.17 112.30 115.09 117.79 122.30 125.90
127.62 130.37 143.59 147.31 151.70 154.73 157.12
163.20 167.43 180.67 189.49 195.20 203.40 211.86
216.00 221.07 221.40 225.09 226.42 229.74 231.13
233.56 235.07 236.94 238.56 243.61 244.71 247.53
253.61 255.75 257.36 260.18 261.18 263.07 263.88
267.43 267.92 271.59 274.42 276.89 279.61 280.65
285.24 289.16 292.03 298.25 300.32 301.63 308.10
308.89 309.77 311.55 315.18 316.73 318.68 320.92
323.18 323.88 328.03 328.88 333.80 335.48 338.60
340.58 342.85 365.47 367.91 377.06 379.34 383.98
386.39 395.36 396.71 399.85 402.16 402.55 408.02
410.02 413.39 415.78 417.76 429.44 432.66 436.46
437.04 438.12 441.61 449.67 452.94 456.70 459.91
474.22 475.66 477.22 478.62 486.36 487.30 504.20
506.74 527.92 528.60 528.66 529.64 565.88 576.46
582.03 585.10 596.86 597.39 600.17 602.84 630.10
631.45 635.73 645.62 649.89 650.39 682.67 685.21
701.07 704.72 706.68 724.26 726.61 787.43 788.83
794.34 794.72 796.80 799.43 808.66 811.94 812.56
813.11 814.12 818.35 818.75 823.40 824.28 824.70
826.14 827.05 864.86 866.95 873.14 906.62 908.03
927.55 939.68 940.33 940.85 941.35 941.60 942.01
942.34 943.83 944.17 945.46 946.60 947.19 947.35
949.21 950.49 950.90 952.96 953.29 954.26 955.29
959.57 960.23 960.42 962.18 963.31 966.78 970.44
972.05 974.01 974.55 1024.35 1025.32 1025.76 1026.51
1031.10 1031.68 1032.97 1033.21 1033.52 1035.59 1036.07
1037.22 1037.84 1038.73 1039.44 1042.69 1064.98 1066.19
1127.56 1130.11 1131.00 1152.97 1154.50 1163.66 1165.47
1184.31 1188.31 1191.75 1194.92 1195.63 1196.94 1199.02
1199.77 1201.77 1202.26 1208.88 1209.45 1212.12 1212.26
1220.66 1221.44 1222.49 1223.67 1224.11 1226.62 1227.31
1228.28 1228.85 1231.81 1233.25 1234.16 1257.56 1258.27
1296.98 1297.20 1328.25 1331.21 1332.53 1363.41 1368.56
1372.49 1372.91 1373.19 1373.32 1374.94 1376.58 1376.95
1377.82 1379.08 1379.31 1379.79 1380.70 1384.08 1385.23
1387.64 1390.79 1396.54 1401.42 1402.44 1402.51 1404.33
1404.76 1406.36 1410.43 1412.63 1413.09 1416.06 1425.14
1427.94 1437.68 1438.50 1440.89 1443.10 1443.67 1444.16
1444.87 1446.38 1447.57 1449.23 1454.18 1457.08 1457.55
1458.21 1459.02 1460.21 1460.75 1461.19 1461.48 1462.38
1462.96 1464.86 1466.58 1467.37 1468.87 1469.15 1469.19
1470.10 1470.74 1470.91 1471.84 1473.12 1473.76 1475.84
1477.66 1477.89 1479.54 1480.71 1482.37 1483.00 1483.58
1484.78 1486.23 1487.83 1492.02 1493.47 1494.66 1496.48
1498.97 1500.75 1502.62 1504.08 1511.00 1513.51 1545.08
1547.84 1637.05 1641.93 1652.94 1658.44 2975.64 2978.28
2981.25 2983.26 2984.01 2985.45 2985.58 2986.16 2987.13
2987.79 2988.33 2989.36 2991.50 2991.97 2992.37 2994.51
2995.17 2995.92 2997.75 3002.04 3006.85 3012.06 3013.62
3022.70 3059.46 3059.80 3061.54 3062.27 3071.85 3076.36
3076.75 3077.67 3077.71 3078.85 3079.60 3079.79 3080.30
3080.90 3081.15 3081.35 3084.06 3086.10 3086.51 3088.31
3091.74 3094.64 3100.99 3102.43 3102.89 3103.85 3104.07
3107.83 3111.91 3112.14 3113.57 3114.40 3116.19 3116.53
3118.12 3118.66 3118.82 3119.55 3121.98 3122.19 3123.56
3123.86 3126.04 3128.86 3132.42 3133.67 3135.30 3138.67
3139.72 3140.75 3141.62 3143.47 3143.69 3149.27 3151.77
3153.67 3164.71 3166.16 3167.45 3167.88

==============================

2-mono

==============================

7.71 22.68 32.40 43.76 50.16 52.39 62.19 70.46 76.29
87.82 95.57 99.98 110.15 114.14 126.52 142.54 144.30
162.01 187.06 188.30 216.36 225.04 227.07 229.65
234.69 240.36 241.33 248.62 255.67 257.63 259.77
262.01 265.10 271.75 276.17 285.37 299.98 300.67
306.76 310.68 315.83 319.93 323.99 326.66 335.71
337.69 365.56 377.26 383.25 389.92 398.56 403.46
412.73 414.56 425.44 430.50 434.90 441.40 453.13
461.25 478.86 480.96 504.79 507.99 519.57 526.56
583.26 595.48 610.82 614.29 628.01 644.36 680.28
703.94 716.10 755.42 783.18 792.72 815.81 819.03
820.26 820.49 820.94 833.82 841.01 852.60 872.18
941.25 941.33 942.68 943.05 948.90 949.75 950.26
951.09 951.74 963.42 964.60 964.85 967.20 968.70
1006.31 1024.13 1025.93 1032.45 1034.69 1035.46 1037.02
1037.50 1040.11 1061.10 1135.39 1152.75 1159.94 1170.33
1182.02 1198.97 1199.62 1201.35 1203.12 1211.13 1212.36
1222.78 1222.96 1226.16 1229.64 1233.86 1235.11 1241.39
1262.56 1318.23 1324.27 1356.60 1370.82 1380.63 1383.90
1385.54 1385.77 1387.14 1388.58 1390.90 1392.02 1394.05
1410.06 1410.44 1415.87 1417.09 1420.59 1424.04 1446.40
1449.27 1451.58 1452.61 1457.62 1458.43 1458.95 1460.31
1461.88 1462.13 1464.13 1466.21 1471.67 1473.23 1474.42
1479.61 1480.35 1480.68 1483.47 1484.42 1486.18 1491.47
1492.79 1497.18 1497.98 1500.32 1537.60 1571.16 1635.25
1671.20 3007.69 3009.32 3009.82 3012.50 3013.36 3015.10
3016.08 3018.13 3018.99 3020.14 3023.66 3025.30 3038.93
3082.62 3084.40 3087.36 3089.14 3089.88 3090.04 3091.59
3092.64 3095.62 3095.67 3098.09 3098.88 3102.41 3110.83
3113.48 3115.05 3119.07 3119.27 3122.21 3123.51 3125.19
3132.83 3134.44 3142.79 3145.37 3158.69 3160.75 3162.72
3185.50 3195.66

==============================

2-dimer

==============================

8.06 17.14 25.54 26.36 27.50 33.03 35.77 38.85 42.59
46.83 47.79 51.46 54.49 55.30 59.99 60.81 62.67 67.06
70.77 73.12 74.99 80.95 84.04 88.15 94.75 97.03 102.06
112.20 113.61 115.00 119.35 120.88 124.62 129.77
132.47 143.41 148.36 150.45 150.94 157.47 166.71
167.74 184.05 192.61 196.30 203.46 210.01 215.83
220.78 223.00 223.81 226.86 228.88 230.21 230.44
232.06 232.55 233.47 237.61 239.01 241.73 244.80
250.23 253.03 255.06 257.45 258.65 259.42 265.13
269.47 270.51 271.95 272.23 273.35 279.77 280.33
288.94 290.17 290.78 300.74 301.27 302.16 305.60
306.01 307.48 310.42 316.86 319.33 324.23 324.66
332.43 334.38 336.01 336.88 338.40 339.89 343.35
346.85 368.68 371.41 377.09 379.79 380.82 385.42
390.04 392.74 398.72 401.88 403.34 405.54 412.53
412.94 413.03 417.04 427.09 427.81 431.26 432.67
433.61 434.62 450.99 452.04 452.73 453.87 478.30
479.87 481.65 482.47 503.40 503.64 504.46 505.60
517.58 517.82 529.27 529.51 578.29 578.86 591.85
592.15 602.70 603.19 609.88 610.73 629.58 630.28
649.63 650.40 679.88 680.37 702.94 703.67 719.70
721.13 749.48 749.98 783.49 784.16 792.24 797.30
806.62 815.09 815.61 818.54 819.14 819.79 820.00
820.35 820.75 822.31 825.38 832.88 834.70 841.43
841.98 853.21 853.86 871.90 878.10 878.90 940.74
941.42 942.27 942.70 943.42 943.62 944.17 945.26
948.70 948.96 949.48 950.17 950.74 951.20 951.84
952.83 953.43 954.24 963.59 963.97 964.91 966.21
966.78 967.14 967.28 967.70 969.53 974.82 980.96
1005.14 1005.58 1025.23 1026.75 1028.56 1029.04 1032.40
1033.19 1033.42 1033.65 1034.37 1034.85 1035.84 1036.20
1038.24 1040.74 1041.17 1041.55 1062.73 1062.98 1139.22
1139.39 1151.56 1154.23 1160.93 1161.17 1167.16 1176.30
1177.26 1186.12 1186.44 1196.41 1196.99 1197.68 1199.17
1199.78 1200.46 1201.02 1202.63 1208.05 1208.60 1212.05
1212.40 1220.80 1221.96 1222.53 1223.36 1225.62 1228.31
1230.55 1231.75 1232.89 1234.17 1234.29 1235.85 1244.08
1244.66 1265.22 1265.46 1316.63 1318.32 1353.68 1358.37
1358.39 1370.70 1371.39 1378.79 1380.28 1382.16 1382.94
1384.60 1385.98 1386.35 1387.40 1387.52 1387.85 1388.52
1389.39 1390.95 1391.11 1395.07 1395.30 1406.65 1409.44
1410.45 1413.86 1413.97 1416.04 1417.18 1417.68 1418.31
1422.40 1422.97 1423.38 1424.22 1445.50 1446.73 1450.45
1450.52 1451.55 1451.85 1452.92 1453.37 1454.42 1456.10
1457.95 1458.59 1459.12 1459.55 1460.31 1461.80 1462.01
1464.55 1465.22 1466.07 1467.10 1467.86 1470.12 1470.56
1471.06 1471.30 1472.00 1473.26 1474.30 1475.59 1475.99
1476.76 1479.71 1480.55 1481.63 1482.43 1483.13 1483.31
1484.41 1486.10 1486.42 1487.51 1489.82 1491.94 1492.64
1494.50 1495.03 1496.35 1499.93 1500.29 1503.43 1505.38
1540.32 1542.43 1578.63 1579.07 1637.30 1637.69 1675.25
1676.17 3002.70 3008.39 3008.98 3011.92 3012.06 3012.55
3014.05 3014.86 3015.69 3016.38 3016.90 3017.97 3018.73
3019.24 3019.69 3020.20 3020.42 3020.81 3023.21 3024.44
3025.18 3025.73 3027.54 3033.23 3037.40 3043.51 3078.31
3085.15 3085.46 3085.65 3087.24 3090.44 3093.05 3093.20
3094.06 3094.23 3095.22 3095.57 3095.71 3095.76 3096.45
3098.52 3098.69 3098.99 3099.26 3099.40 3101.55 3101.97
3107.84 3112.06 3113.50 3114.91 3115.99 3117.21 3118.27
3119.59 3120.66 3121.74 3123.51 3124.10 3128.59 3129.63
3129.91 3132.47 3132.88 3133.81 3135.61 3139.99 3141.88
3142.39 3146.00 3147.82 3148.51 3149.49 3149.99 3158.98
3161.35 3161.84 3162.78 3164.00 3165.15 3166.89 3190.30
3190.54 3198.02 3198.03

==============================

4-mono

==============================

14.86 18.46 36.96 38.91 47.03 49.25 60.22 72.72
78.92 80.69 87.80 96.85 100.39 114.32 130.85 140.06
149.69 152.58 178.10 179.47 210.41 211.31 221.81
225.50 230.23 232.81 240.62 241.86 245.36 255.43
259.12 261.69 270.99 272.56 275.17 283.45 286.34
298.62 303.10 315.69 318.68 326.08 329.63 330.49
336.62 348.69 364.53 379.43 382.61 386.55 396.53
400.10 408.18 414.83 416.78 423.43 432.04 432.88
434.97 454.42 480.82 483.65 504.34 511.20 516.97
526.58 573.66 586.69 603.81 612.32 614.90 641.98
672.36 708.83 757.49 765.09 796.91 801.38 817.29
818.38 818.71 818.93 820.17 839.94 849.96 858.21
887.32 939.49 939.57 941.31 942.15 949.39 949.82
950.51 951.08 965.46 965.68 966.93 967.27 972.50
1009.71 1024.05 1024.40 1024.99 1032.02 1033.74 1034.84
1036.02 1038.13 1038.62 1049.87 1150.36 1155.67 1166.52
1171.87 1179.77 1183.94 1195.13 1195.87 1199.37 1200.71
1210.79 1210.93 1222.71 1223.04 1224.22 1224.61 1234.55
1235.03 1251.66 1258.71 1304.73 1348.25 1354.62 1358.64
1388.44 1389.03 1390.20 1390.94 1394.35 1394.95 1395.48
1395.68 1405.54 1409.82 1415.31 1420.04 1421.13 1426.20
1426.99 1451.98 1452.51 1453.43 1454.10 1456.92 1459.12
1459.64 1460.00 1460.15 1461.70 1463.20 1466.39 1467.14
1467.55 1475.14 1476.01 1477.65 1481.98 1482.36 1483.90
1484.69 1487.18 1488.05 1493.96 1495.10 1498.86 1499.28
1547.99 1594.77 1659.22 1661.44 3018.05 3020.69 3020.72
3022.57 3024.32 3024.59 3027.25 3027.94 3029.22 3029.95
3037.56 3038.64 3047.71 3049.65 3094.85 3096.05 3097.39
3098.29 3099.59 3100.72 3100.80 3103.03 3104.51 3104.90
3105.00 3109.38 3109.85 3110.05 3111.08 3111.86 3113.65
3119.36 3128.33 3129.01 3132.79 3133.76 3146.66 3146.71
3148.27 3150.50 3185.49 3187.30 3205.67 3205.85

==============================

4-dimer

==============================

9.24 12.42 21.89 23.91 27.21 32.74 33.73 39.19 41.97
43.69 44.89 48.36 53.22 54.58 59.42 60.34 61.84 65.22
67.40 70.12 73.89 78.52 79.50 86.19 89.02 96.44 98.48
102.50 104.50 110.22 111.34 115.36 121.38 124.63
130.94 136.68 143.96 150.99 152.72 155.11 157.80
160.47 166.26 178.85 190.30 192.89 204.87 205.12
212.39 218.85 221.06 222.32 224.35 227.22 230.04
231.16 232.69 236.12 238.06 239.55 243.79 248.91
249.14 251.39 252.33 257.31 257.73 262.06 262.79
267.60 268.00 271.47 274.11 274.63 276.87 281.65
283.78 292.15 296.78 297.09 302.88 304.93 308.17
309.53 313.20 315.05 316.23 324.74 327.03 327.36
328.09 331.47 338.16 339.36 344.50 346.48 348.02
353.76 364.36 367.83 377.77 379.21 383.79 384.68
385.81 387.24 401.46 402.46 405.21 411.22 412.21
413.40 414.56 416.57 424.95 425.90 429.83 430.21
432.31 432.89 434.68 436.58 451.34 452.56 480.92
481.78 484.64 486.73 501.97 506.87 509.28 513.62
515.56 516.51 523.48 526.30 574.02 576.40 583.53
588.23 602.38 604.61 609.61 612.70 616.18 617.96
640.74 644.61 660.42 671.75 712.78 721.58 730.00
746.74 759.01 759.48 765.75 799.16 799.32 801.03
802.27 816.60 816.65 816.76 817.85 819.12 820.30
820.65 822.29 822.36 829.00 841.86 844.14 847.03
847.90 856.07 858.22 859.75 888.39 888.96 937.18
939.56 939.79 940.34 941.19 941.79 943.74 944.59
949.72 949.94 950.36 950.56 951.52 951.80 951.89
954.53 954.76 965.96 966.49 967.44 967.67 969.94
970.09 970.65 971.36 974.77 975.80 1011.40 1011.88
1025.27 1026.16 1026.50 1026.75 1027.28 1028.84 1031.28
1032.57 1032.77 1033.54 1034.31 1035.66 1036.82 1037.52
1038.48 1039.06 1040.60 1047.90 1048.47 1050.17 1128.04
1142.20 1147.99 1153.71 1155.24 1165.49 1165.74 1169.04
1169.79 1182.37 1183.89 1185.78 1187.14 1192.48 1193.51
1194.91 1196.26 1197.20 1197.76 1200.62 1204.53 1210.00
1210.07 1211.00 1211.07 1222.71 1222.85 1223.39 1224.03
1224.25 1225.98 1228.68 1233.96 1234.12 1237.12 1237.87
1239.99 1250.78 1251.05 1254.99 1256.98 1300.28 1303.55
1343.85 1345.94 1351.49 1351.53 1379.63 1387.50 1387.79
1388.16 1389.19 1390.29 1390.53 1390.86 1391.52 1393.02
1393.37 1394.40 1394.86 1395.80 1396.02 1396.64 1398.04
1398.56 1406.00 1411.83 1413.79 1417.20 1417.76 1419.53
1420.12 1423.28 1424.40 1426.33 1426.61 1426.95 1428.14
1430.11 1449.17 1450.71 1451.88 1452.83 1453.68 1454.00
1454.72 1455.43 1456.49 1456.94 1457.34 1458.43 1459.29
1459.86 1460.95 1461.28 1461.89 1462.13 1462.63 1463.18

1464.80 1465.31 1465.96 1466.37 1468.62 1469.16 1470.62
1472.63 1475.63 1477.03 1478.80 1479.15 1480.84 1482.15
1482.29 1482.71 1483.13 1485.33 1485.89 1486.65 1487.37
1487.90 1488.30 1488.66 1492.88 1494.85 1495.14 1496.96
1500.02 1501.36 1502.75 1503.87 1548.09 1549.95 1583.22
1588.45 1652.96 1655.62 1657.30 1659.94 3018.48 3018.71
3019.14 3021.43 3023.13 3025.02 3026.42 3027.16 3027.30
3027.65 3028.07 3028.72 3029.22 3030.94 3031.32 3033.37
3033.69 3034.09 3034.42 3036.74 3036.91 3037.59 3040.45
3048.49 3048.73 3050.60 3069.81 3089.44 3097.29 3098.16
3099.23 3099.59 3100.70 3100.82 3101.69 3102.00 3102.60
3102.81 3103.20 3103.40 3103.95 3104.78 3106.08 3106.32
3107.61 3108.09 3108.75 3109.03 3110.65 3111.75 3112.01
3112.68 3113.03 3113.94 3116.50 3117.75 3118.09 3126.40
3129.46 3130.07 3131.64 3133.93 3134.82 3135.05 3135.59
3135.68 3136.95 3138.14 3140.59 3143.56 3145.64 3146.75
3147.05 3147.29 3149.61 3150.77 3150.98 3153.30 3153.61
3183.37 3188.24 3188.74 3190.35 3190.53 3209.00 3209.48
3209.96 3210.66

3. References

[1] R. L. M. Bienenmann, A. J. Schanz, P. L. Ooms, M. Lutz, D. L. J. Broere, *Angew. Chem. Int. Ed.* **2022**, *61*, e202202318.
[2] E. Kounalis, M. Lutz, D. L. J. Broere, *Chem. Eur. J.* **2019**, *25*, 13280–13284.
[3] M. Brookhart, B. Grant, A. F. Volpe Jr, *Organometallics* **1992**, *11*, 3920–3922.
[4] A. M. M. Schreurs, X. Xian, L. M. J. Kroon-Batenburg, *J. Appl. Crystallogr.* **2009**, *43*, 70–82.
[5] G. M. Sheldrick, *Göttingen, Germany* **2014**.
[6] G. M. Sheldrick, *Acta Crystallogr. A Found. Adv.* **2015**, *71*, 3–8.
[7] G. M. Sheldrick, *Acta Crystallogr. B* **2015**, *71*, 3–8.
[8] A. L. Spek, *Acta Crystallogr. D Biol. Crystallogr.* **2009**, *65*, 148–155.
[9] R. G. Parr, Y. Weitao, *Density-Functional Theory of Atoms and Molecules*, Oxford University Press, **1994**.
[10] F. Neese, F. Wennmohs, U. Becker, C. Riplinger, *J. Chem. Phys.* **2020**, *152*, 224108.
[11] J. C. Slater, *The Self-Consistent Field for Molecules and Solids*, McGraw-Hill, **1974**.
[12] S. H. Vosko, L. Wilk, M. Nusair, *Can. J. Phys.* **1980**, *58*, 1200–1211.
[13] A. D. Becke, *Phys. Rev. A Gen. Phys.* **1988**, *38*, 3098–3100.
[14] C. Lee, W. Yang, R. G. Parr, *Phys. Rev. B Condens. Matter* **1988**, *37*, 785–789.
[15] A. D. Becke, *J. Chem. Phys.* **1992**.
[16] A. D. Becke, *J. Chem. Phys.* **1993**, *98*, 5648–5652.
[17] S. Grimme, S. Ehrlich, L. Goerigk, *J. Comput. Chem.* **2011**, *32*, 1456–1465.
[18] F. Weigend, *Phys. Chem. Chem. Phys.* **2006**, *8*, 1057–1065.
[19] F. Neese, F. Wennmohs, A. Hansen, U. Becker, *Chem. Phys.* **2009**, *356*, 98–109.
[20] F. Weigend, R. Ahlrichs, *Phys. Chem. Chem. Phys.* **2005**, *7*, 3297–3305.
[21] V. Barone, M. Cossi, *J. Phys. Chem. A* **1998**, *102*, 1995–2001.
[22] F. M. Bickelhaupt, E. J. Baerends, *Rev. Comput. Chem.* **2000**, 1–86.
[23] F. M. Bickelhaupt, K. N. Houk, *Angew. Chem. Int. Ed.* **2017**, *56*, 10070–10086.
[24] G. Te Velde, F. M. Bickelhaupt, E. J. Baerends, C. Fonseca Guerra, S. J. A. van Gisbergen, J. G. Snijders, T. Ziegler, *J. Comput. Chem.* **2001**, *22*, 931–967.
[25] W. Kutzelnigg, U. Fleischer, M. Schindler, in *Deuterium and Shift Calculation* (Eds.: U. Fleischer, W. Kutzelnigg, H.-H. Limbach, G.J. Martin, M.L. Martin, M. Schindler), Springer Berlin Heidelberg, **1991**, pp. 165–262.
[26] G. A. Andrienko, *Chemcraft - Graphical Software for Visualization of Quantum Chemistry Computations*
[27] J. D. Hunter, *Comput. Sci. Eng.* **2007**, *9*, 90–95.
