# Supplementary material for: Protonation‐Induced Electron Density Redistribution Facilitates Aggregation in PNNP Dicopper Hydride Complexes
Source: Chemistry. 2025 Dec 16;32(30):e02507. doi: 10.1002/chem.202502507 (PMC13472354; doi:10.1002/chem.202502507)

checkCIF/PLATON report

Structure factors have been supplied for datablock(s) l1144a, l1148a

THIS REPORT IS FOR GUIDANCE ONLY. IF USED AS PART OF A REVIEW PROCEDURE FOR PUBLICATION, IT SHOULD NOT REPLACE THE EXPERTISE OF AN EXPERIENCED CRYSTALLOGRAPHIC REFEREE.

No syntax errors found. CIF dictionary Interpreting this report

Datablock: l1144a

Bond precision:	C-C = 0.0033 A		Wavelength=0.71073
Cell:	a=21.1039(3)	b=20.0450(3)	c=21.7343(4)
	alpha=90	beta=91.370(1)	gamma=90
Temperature:	150 K		

	Calculated	Reported
Volume	9191.6(3)	9191.6(3)
Space group	P 21/n	P 21/n
Hall group	-P 2yn	-P 2yn
Moiety formula	C32 H12 B F24, C52 H89 Cu4 N4 P4	C52 H89 Cu4 N4 P4, C32 H12 B F24
Sum formula	C84 H101 B Cu4 F24 N4 P4	C84 H101 B Cu4 F24 N4 P4
Mr	2011.58	2011.53
Dx,g cm-3	1.454	1.454
Z	4	4
Mu (mm-1)	1.075	1.075
F000	4120.0	4120.0
F000'	4128.99	
h,k,lmax	27,26,28	27,26,28
Nref	21124	21122
Tmin,Tmax	0.668,0.928	0.673,0.746
Tmin'	0.624	

Correction method= # Reported T Limits: Tmin=0.673 Tmax=0.746
AbsCorr = MULTI-SCAN

Data completeness= 1.000 Theta(max)= 27.499

R(reflections)= 0.0345(17317) wR2(reflections)= 0.0924(21122)

S = 1.031 Npar= 1234

The following ALERTS were generated. Each ALERT has the format
test-name_ALERT_alert-type_alert-level.
Click on the hyperlinks for more details of the test.

Alert level C

```
PLAT042_ALERT_1_C Calc. and Reported MoietyFormula Strings  Differ     Please Check
              Calc: C32 H12 B F24, C52 H89 Cu4 N4 P4
              Rep.: C52 H89 Cu4 N4 P4, C32 H12 B F24
PLAT213_ALERT_2_C Atom F4B              has ADP max/min Ratio .....        3.2 prolat
PLAT213_ALERT_2_C Atom F5B              has ADP max/min Ratio .....        3.9 prolat
PLAT213_ALERT_2_C Atom F6B              has ADP max/min Ratio .....        3.1 prolat
PLAT213_ALERT_2_C Atom F19B             has ADP max/min Ratio .....        3.2 prolat
PLAT213_ALERT_2_C Atom F20B             has ADP max/min Ratio .....        4.0 prolat
PLAT213_ALERT_2_C Atom F21B             has ADP max/min Ratio .....        3.3 prolat
PLAT220_ALERT_2_C NonSolvent   Resd 2  C   Ueq(max)/Ueq(min) Range         4.1 Ratio
PLAT222_ALERT_3_C NonSolvent Resd 2  H   Uiso(max)/Uiso(min) Range         4.2 Ratio
PLAT242_ALERT_2_C Low    'MainMol' Ueq as Compared to Neighbors of        C152 Check
```

Alert level G

```
PLAT002_ALERT_2_G Number of Distance or Angle Restraints on AtSite          52 Note
PLAT003_ALERT_2_G Number of Uiso or Uij Restrained non-H Atoms ...          36 Report
PLAT083_ALERT_2_G SHELXL Second Parameter in WGHT  Unusually Large        6.48 Why ?
PLAT176_ALERT_4_G The CIF-Embedded .res File Contains SADI Records           3 Report
PLAT186_ALERT_4_G The CIF-Embedded .res File Contains ISOR Records           1 Report
PLAT187_ALERT_4_G The CIF-Embedded .res File Contains RIGU Records           1 Report
PLAT191_ALERT_3_G A Non-default SADI Restraint Value has been used      0.0100 Report
PLAT230_ALERT_2_G Hirshfeld Test Diff for    F2A      --C73      .         7.5 s.u.
PLAT230_ALERT_2_G Hirshfeld Test Diff for    F6A      --C83      .         9.7 s.u.
PLAT242_ALERT_2_G Low    'MainMol' Ueq as Compared to Neighbors of         C73 Check
PLAT242_ALERT_2_G Low    'MainMol' Ueq as Compared to Neighbors of         C83 Check
PLAT242_ALERT_2_G Low    'MainMol' Ueq as Compared to Neighbors of        C153 Check
PLAT242_ALERT_2_G Low    'MainMol' Ueq as Compared to Neighbors of        C163 Check
PLAT242_ALERT_2_G Low    'MainMol' Ueq as Compared to Neighbors of        C233 Check
PLAT242_ALERT_2_G Low    'MainMol' Ueq as Compared to Neighbors of        C243 Check
PLAT242_ALERT_2_G Low    'MainMol' Ueq as Compared to Neighbors of        C313 Check
PLAT242_ALERT_2_G Low    'MainMol' Ueq as Compared to Neighbors of        C323 Check
PLAT301_ALERT_3_G Main Residue  Disorder ..............(Resd    1)         21% Note
PLAT303_ALERT_2_G Full Occupancy Atom H1        with # Connections        3.00 Check
PLAT303_ALERT_2_G Full Occupancy Atom H2        with # Connections        3.00 Check
PLAT434_ALERT_2_G Short Inter HL..HL Contact F93      ..F4B      .        2.81 Ang.
                                      1/2-x,-1/2+y,3/2-z  =      2_546 Check
PLAT434_ALERT_2_G Short Inter HL..HL Contact F163     ..F3B      .        2.77 Ang.
                                      1/2-x,-1/2+y,3/2-z  =      2_546 Check
PLAT434_ALERT_2_G Short Inter HL..HL Contact F183     ..F3B      .        2.84 Ang.
                                      1/2-x,-1/2+y,3/2-z  =      2_546 Check
PLAT434_ALERT_2_G Short Inter HL..HL Contact F223     ..F14A     .        2.78 Ang.
                                      1/2-x,-1/2+y,3/2-z  =      2_546 Check
PLAT794_ALERT_5_G Tentative Bond Valency for Cu11      (I)       .        0.52 Info
PLAT794_ALERT_5_G Tentative Bond Valency for Cu12      (I)       .        0.48 Info
PLAT860_ALERT_3_G Number of Least-Squares Restraints ............         2700 Note
PLAT899_ALERT_4_G SHELXL2018 is Deprecated and Succeeded by SHELXL      2019/3 Note
PLAT912_ALERT_4_G Missing # of FCF Reflections Above STh/L=  0.600           1 Note
PLAT965_ALERT_2_G The SHELXL WEIGHT Optimisation has not Converged      Please Check
PLAT969_ALERT_5_G The 'Henn et al.' R-Factor-gap value ..........         3.88 Note
```

```
             Predicted wR2: Based on SigI**2  2.38 or SHELX Weight  9.24
PLAT978_ALERT_2_G Number C-C Bonds with Positive Residual Density.         2 Info
```

```
   0 ALERT level A = Most likely a serious problem - resolve or explain
   0 ALERT level B = A potentially serious problem, consider carefully
  10 ALERT level C = Check. Ensure it is not caused by an omission or oversight
  32 ALERT level G = General information/check it is not something unexpected

   1 ALERT type 1 CIF construction/syntax error, inconsistent or missing data
  29 ALERT type 2 Indicator that the structure model may be wrong or deficient
   4 ALERT type 3 Indicator that the structure quality may be low
   5 ALERT type 4 Improvement, methodology, query or suggestion
   3 ALERT type 5 Informative message, check
```

Datablock: l1148a

Bond precision: C-C = 0.0106 A Wavelength=0.71073

Cell:	a=15.8777(6)	b=20.8543(9)	c=22.3701(8)
	alpha=93.061(1)	beta=110.063(1)	gamma=109.979(1)
Temperature:	150 K		

	Calculated	Reported
Volume	6415.7(4)	6415.6(4)
Space group	P -1	P -1
Hall group	-P 1	-P 1
Moiety formula	2(C32 H12 B F24), C52 H90 Cu4 N4 P4	C52 H90 Cu4 N4 P4, 2(C32 H12 B F24)
Sum formula	C116 H114 B2 Cu4 F48 N4 P4	C116 H114 B2 Cu4 F48 N4 P4
Mr	2875.82	2875.77
Dx,g cm-3	1.489	1.489
Z	2	2
Mu (mm-1)	0.821	0.821
F000	2912.0	2912.0
F000’	2917.44	
h,k,lmax	19,25,26	19,25,26
Nref	23171	23167
Tmin,Tmax	0.821,0.952	0.621,0.745
Tmin’	0.756	

Correction method= # Reported T Limits: Tmin=0.621 Tmax=0.745
AbsCorr = MULTI-SCAN

Data completeness= 1.000 Theta(max)= 25.239

R(reflections)= 0.0710(11342)	wR2(reflections)= 0.1889(23167)
S = 1.023 Npar= 1762	

The following ALERTS were generated. Each ALERT has the format
test-name_ALERT_alert-type_alert-level.
Click on the hyperlinks for more details of the test.

Alert level B

```
PLAT934_ALERT_3_B Number of (Iobs-Icalc)/Sigma(W) > 10 Outliers ..          2 Check
               -3   1   0,   -3   2   1,
```

Author Response: In these outliers, the observed structure factors are larger than the calculated structure factors. Small additional crystal fragments were present but were not taken into account during indexing and intensity determination. The interfering intensities of the non-indexed fragments are causing the outliers.

Alert level C

```
RINTA01_ALERT_3_C  The value of Rint is greater than 0.12
            Rint given   0.121
PLAT020_ALERT_3_C The Value of Rint is Greater Than 0.12 .........      0.121 Report
PLAT026_ALERT_3_C Ratio Observed / Unique Reflections (too) Low ..        49% Check
PLAT042_ALERT_1_C Calc. and Reported MoietyFormula Strings  Differ     Please Check
              Calc: 2(C32 H12 B F24), C52 H90 Cu4 N4 P4
              Rep.: C52 H90 Cu4 N4 P4, 2(C32 H12 B F24)
PLAT213_ALERT_2_C Atom C242            has ADP max/min Ratio .....        3.5 oblate
PLAT213_ALERT_2_C Atom F53             has ADP max/min Ratio .....        3.1 prolat
PLAT213_ALERT_2_C Atom F2B             has ADP max/min Ratio .....        3.4 prolat
PLAT220_ALERT_2_C NonSolvent   Resd 2  C   Ueq(max)/Ueq(min) Range        3.9 Ratio
PLAT241_ALERT_2_C High   'MainMol' Ueq as Compared to Neighbors of        C11 Check
PLAT242_ALERT_2_C Low    'MainMol' Ueq as Compared to Neighbors of       C111 Check
PLAT242_ALERT_2_C Low    'MainMol' Ueq as Compared to Neighbors of       C151 Check
PLAT242_ALERT_2_C Low    'MainMol' Ueq as Compared to Neighbors of       C152 Check
PLAT341_ALERT_3_C Low Bond Precision on  C-C Bonds ...............    0.01063 Ang.
PLAT601_ALERT_2_C Unit Cell Contains Solvent Accessible VOIDS of .         42 Ang**3
PLAT911_ALERT_3_C Missing FCF Refl Between Thmin & STh/L=    0.600          2 Report
                1   3   3,   -9  -9  24,
```

Alert level G

```
PLAT002_ALERT_2_G Number of Distance or Angle Restraints on AtSite         95 Note
PLAT003_ALERT_2_G Number of Uiso or Uij Restrained non-H Atoms ...        179 Report
PLAT007_ALERT_5_G Number of Unrefined Donor-H Atoms .............           2 Report
              H1    H2
PLAT083_ALERT_2_G SHELXL Second Parameter in WGHT  Unusually Large       8.38 Why ?
PLAT154_ALERT_1_G The s.u.'s on the Cell Angles are Equal ..(Note)      0.001 Degree
PLAT169_ALERT_4_G The CIF-Embedded .res File Contains AFIX 1 Recds          1 Report
PLAT176_ALERT_4_G The CIF-Embedded .res File Contains SADI Records          3 Report
```

```
PLAT186_ALERT_4_G The CIF-Embedded .res File Contains ISOR Records          2 Report
PLAT187_ALERT_4_G The CIF-Embedded .res File Contains RIGU Records          1 Report
PLAT191_ALERT_3_G A Non-default SADI Restraint Value has been used     0.0100 Report
PLAT242_ALERT_2_G Low    'MainMol' Ueq as Compared to Neighbors of        C74 Check
PLAT242_ALERT_2_G Low    'MainMol' Ueq as Compared to Neighbors of        C84 Check
PLAT242_ALERT_2_G Low    'MainMol' Ueq as Compared to Neighbors of       C154 Check
PLAT242_ALERT_2_G Low    'MainMol' Ueq as Compared to Neighbors of       C164 Check
PLAT242_ALERT_2_G Low    'MainMol' Ueq as Compared to Neighbors of       C234 Check
PLAT242_ALERT_2_G Low    'MainMol' Ueq as Compared to Neighbors of       C244 Check
PLAT242_ALERT_2_G Low    'MainMol' Ueq as Compared to Neighbors of       C324 Check
PLAT242_ALERT_2_G Low    'MainMol' Ueq as Compared to Neighbors of        C73 Check
PLAT242_ALERT_2_G Low    'MainMol' Ueq as Compared to Neighbors of        C83 Check
PLAT242_ALERT_2_G Low    'MainMol' Ueq as Compared to Neighbors of       C153 Check
PLAT242_ALERT_2_G Low    'MainMol' Ueq as Compared to Neighbors of       C163 Check
PLAT242_ALERT_2_G Low    'MainMol' Ueq as Compared to Neighbors of       C233 Check
PLAT242_ALERT_2_G Low    'MainMol' Ueq as Compared to Neighbors of       C243 Check
PLAT242_ALERT_2_G Low    'MainMol' Ueq as Compared to Neighbors of       C313 Check
PLAT242_ALERT_2_G Low    'MainMol' Ueq as Compared to Neighbors of       C323 Check
PLAT300_ALERT_4_G Atom Site Occupancy of F13C       Constrained at      0.502 Check
PLAT300_ALERT_4_G Atom Site Occupancy of F14C       Constrained at      0.502 Check
PLAT300_ALERT_4_G Atom Site Occupancy of F15C       Constrained at      0.502 Check
PLAT300_ALERT_4_G Atom Site Occupancy of F19C       Constrained at       0.52 Check
PLAT300_ALERT_4_G Atom Site Occupancy of F20C       Constrained at       0.52 Check
PLAT300_ALERT_4_G Atom Site Occupancy of F21C       Constrained at       0.52 Check
PLAT300_ALERT_4_G Atom Site Occupancy of F22C       Constrained at       0.52 Check
PLAT300_ALERT_4_G Atom Site Occupancy of F23C       Constrained at       0.52 Check
PLAT300_ALERT_4_G Atom Site Occupancy of F24C       Constrained at       0.52 Check
PLAT300_ALERT_4_G Atom Site Occupancy of F13D       Constrained at      0.498 Check
PLAT300_ALERT_4_G Atom Site Occupancy of F14D       Constrained at      0.498 Check
PLAT300_ALERT_4_G Atom Site Occupancy of F15D       Constrained at      0.498 Check
PLAT300_ALERT_4_G Atom Site Occupancy of F19D       Constrained at       0.48 Check
PLAT300_ALERT_4_G Atom Site Occupancy of F20D       Constrained at       0.48 Check
PLAT300_ALERT_4_G Atom Site Occupancy of F21D       Constrained at       0.48 Check
PLAT300_ALERT_4_G Atom Site Occupancy of F22D       Constrained at       0.48 Check
PLAT300_ALERT_4_G Atom Site Occupancy of F23D       Constrained at       0.48 Check
PLAT300_ALERT_4_G Atom Site Occupancy of F24D       Constrained at       0.48 Check
PLAT300_ALERT_4_G Atom Site Occupancy of F1A        Constrained at       0.56 Check
PLAT300_ALERT_4_G Atom Site Occupancy of F2A        Constrained at       0.56 Check
PLAT300_ALERT_4_G Atom Site Occupancy of F3A        Constrained at       0.56 Check
PLAT300_ALERT_4_G Atom Site Occupancy of F10A       Constrained at       0.63 Check
PLAT300_ALERT_4_G Atom Site Occupancy of F11A       Constrained at       0.63 Check
PLAT300_ALERT_4_G Atom Site Occupancy of F12A       Constrained at       0.63 Check
PLAT300_ALERT_4_G Atom Site Occupancy of F1B        Constrained at       0.44 Check
PLAT300_ALERT_4_G Atom Site Occupancy of F2B        Constrained at       0.44 Check
PLAT300_ALERT_4_G Atom Site Occupancy of F3B        Constrained at       0.44 Check
PLAT300_ALERT_4_G Atom Site Occupancy of F10B       Constrained at       0.37 Check
PLAT300_ALERT_4_G Atom Site Occupancy of F11B       Constrained at       0.37 Check
PLAT300_ALERT_4_G Atom Site Occupancy of F12B       Constrained at       0.37 Check
PLAT301_ALERT_3_G Main Residue  Disorder .............(Resd    1)        16% Note
PLAT301_ALERT_3_G Main Residue  Disorder .............(Resd    3)        11% Note
PLAT303_ALERT_2_G Full Occupancy Atom H1        with # Connections       3.00 Check
PLAT303_ALERT_2_G Full Occupancy Atom H2        with # Connections       3.00 Check
PLAT432_ALERT_2_G Short Inter X...Y Contact  F34      ..C103     .       2.94 Ang.
                                              1+x,y,z  =      1_655 Check
PLAT794_ALERT_5_G Tentative Bond Valency for Cu11      (I)       .       0.48 Info
PLAT794_ALERT_5_G Tentative Bond Valency for Cu21      (I)       .       0.44 Info
PLAT794_ALERT_5_G Tentative Bond Valency for Cu22      (I)       .       0.45 Info
```

```
PLAT860_ALERT_3_G Number of Least-Squares Restraints ............       8529 Note
PLAT899_ALERT_4_G SHELXL2018 is Deprecated and Succeeded by SHELXL     2019/3 Note
PLAT910_ALERT_3_G Missing # of FCF Reflection(s) Below Theta(Min).         2 Note
                0  1  0,    0  0  1,
PLAT941_ALERT_3_G Average HKL Measurement Multiplicity ..........        3.4 Low
PLAT965_ALERT_2_G The SHELXL WEIGHT Optimisation has not Converged     Please Check
PLAT969_ALERT_5_G The 'Henn et al.' R-Factor-gap value ..........       2.19 Note
              Predicted wR2: Based on SigI**2  8.62 or SHELX Weight 19.22
PLAT978_ALERT_2_G Number C-C Bonds with Positive Residual Density.         0 Info
```

```
   0 ALERT level A = Most likely a serious problem - resolve or explain
   1 ALERT level B = A potentially serious problem, consider carefully
  15 ALERT level C = Check. Ensure it is not caused by an omission or oversight
  70 ALERT level G = General information/check it is not something unexpected

   2 ALERT type 1 CIF construction/syntax error, inconsistent or missing data
  32 ALERT type 2 Indicator that the structure model may be wrong or deficient
  12 ALERT type 3 Indicator that the structure quality may be low
  35 ALERT type 4 Improvement, methodology, query or suggestion
   5 ALERT type 5 Informative message, check
```

It is advisable to attempt to resolve as many as possible of the alerts in all categories. Often the minor alerts point to easily fixed oversights, errors and omissions in your CIF or refinement strategy, so attention to these fine details can be worthwhile. In order to resolve some of the more serious problems it may be necessary to carry out additional measurements or structure refinements. However, the purpose of your study may justify the reported deviations and the more serious of these should normally be commented upon in the discussion or experimental section of a paper or in the "special_details" fields of the CIF. checkCIF was carefully designed to identify outliers and unusual parameters, but every test has its limitations and alerts that are not important in a particular case may appear. Conversely, the absence of alerts does not guarantee there are no aspects of the results needing attention. It is up to the individual to critically assess their own results and, if necessary, seek expert advice.

Publication of your CIF in IUCr journals

A basic structural check has been run on your CIF. These basic checks will be run on all CIFs submitted for publication in IUCr journals (*Acta Crystallographica*, *Journal of Applied Crystallography*, *Journal of Synchrotron Radiation*); however, if you intend to submit to *Acta Crystallographica Section C* or *E* or *IUCrData*, you should make sure that full publication checks are run on the final version of your CIF prior to submission.

Publication of your CIF in other journals

Please refer to the *Notes for Authors* of the relevant journal for any special instructions relating to CIF submission.

PLATON version of 06/01/2024; check.def file version of 05/01/2024

Datablock l1144a - ellipsoid plot

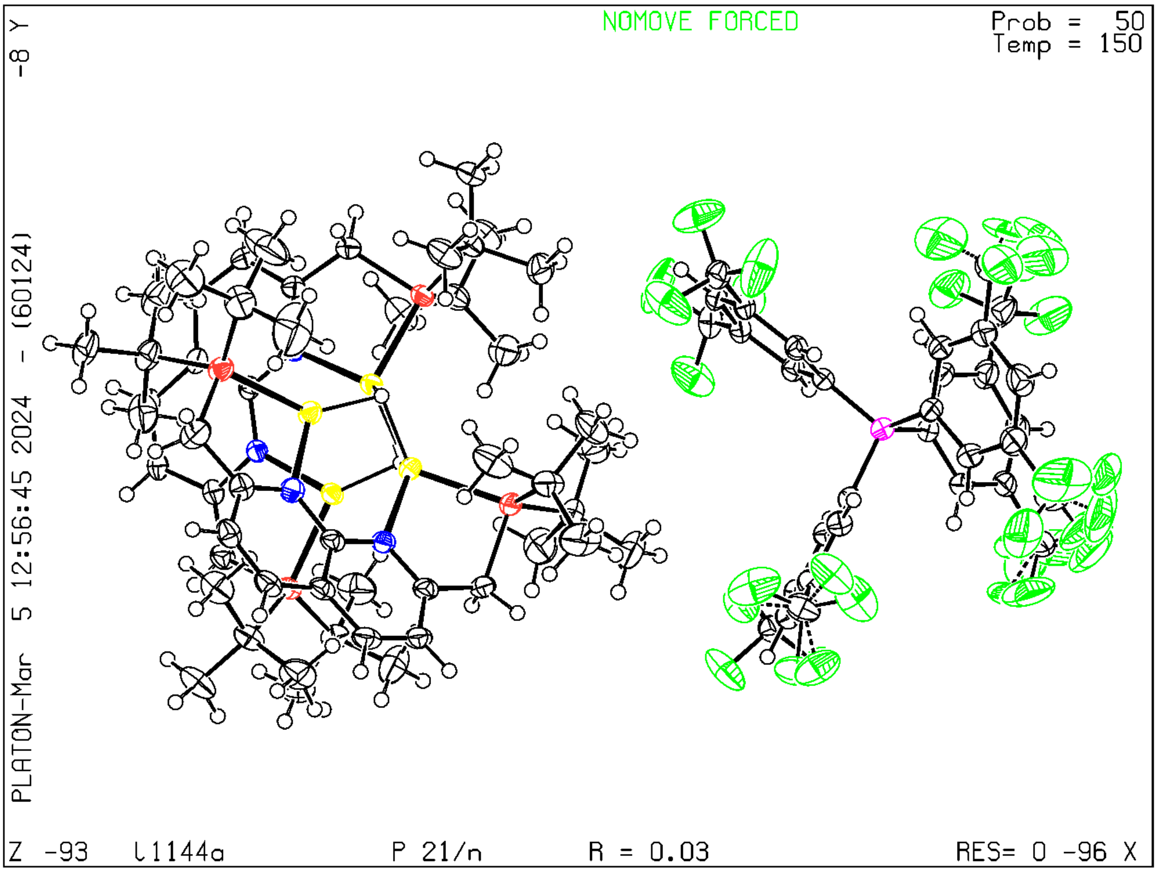

Datablock l1148a - ellipsoid plot

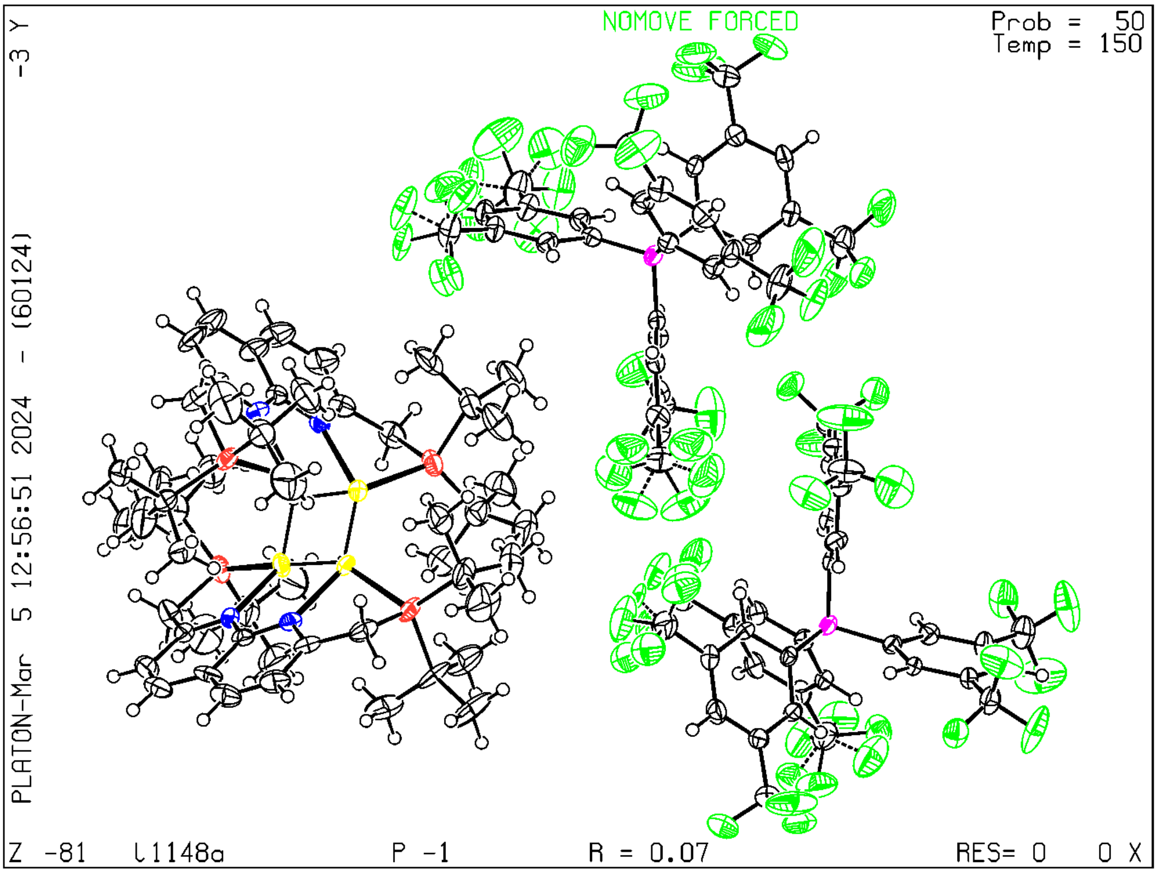

Supplement: Supplementary file 2 [file CHEM-32-e02507-s001.zip › checkcif.pdf]
